# From global to local: Developing a context-specific BeSD-HPV tool through cultural and linguistic adaptation in Pakistan

**DOI:** 10.1371/journal.pone.0350162

**Published:** 2026-06-15

**Authors:** Khola Noreen, Mehreen Noor, Saba Maryam, Mukhtiar Baig

**Affiliations:** 1 Department of Community Medicine and Public Health, Rawalpindi Medical University, Rawalpindi, Pakistan; 2 Department of Public Health, Health Services Academy, Islamabad, Pakistan; 3 Department of Clinical Biochemistry, Faculty of Medicine, King Abdulaziz University, Rabigh, Saudi Arabia; Federal University Otuoke, NIGERIA

## Abstract

**Objectives:**

A key challenge in preventing cervical cancer is the low uptake of the Human Papillomavirus (HPV) vaccine in low and middle-income countries (LMICs). Evidence indicates that, in addition to logistical and structural issues, this is often influenced by sociocultural factors. The objective of this study was to develop a culturally adapted Behavioral and Social Drivers (BeSD) of HPV vaccination framework that is linguistically appropriate, contextually grounded, and culturally sensitive, to evaluate the social, behavioral, and cultural factors influencing HPV vaccination in Pakistan.

**Methods:**

This descriptive qualitative survey was conducted across several districts in Punjab, Pakistan, from February to August 2025. Study participants included adolescent girls, parents/guardians, healthcare providers, community workers, teachers, school administrators, and religious scholars.

**Results:**

Data were analyzed using Braun and Clarke’s six-step thematic analysis with deductive and inductive coding. Deductive codes aligned with existing WHO BeSD domains, while inductive analysis revealed a new domain: *Cultural Integration.* Themes and subthemes were mapped to specific BeSD constructs, illustrated with participant quotes and rationales. The tool was subsequently translated into Urdu by a bilingual expert to ensure linguistic appropriateness. Table cell colors correspond to the WHO BeSD domains (Thinking and Feeling, Social Processes, Motivation, Practical Issues) and the emergent theme, Cultural Integration.

**Conclusion:**

The culturally adapted BeSD-HPV tool provides a methodological framework for contextualizing global health models. It underscores the need for culturally informed, community-driven strategies to ensure the successful rollout of HPV vaccination in Pakistan and other LMICs.

## Introduction

The World Health Organization’s (WHO) vision to eliminate cervical cancer by 2030 will remain out of reach for Pakistan unless Human Papillomavirus (HPV) vaccination strategies are designed to reflect the nation’s unique cultural, social, and health system realities. The issue of HPV vaccine introduction in Pakistan cannot be addressed only in terms of logistics; rather, it is a behavioral issue that requires cultural understanding, trust-building, and specific interventions based on the Behavioral and Social Drivers (BeSD) framework. Cervical cancer is among the few cancers that can be successfully prevented by vaccination and early screening, but this disease continues to be a leading cause of morbidity and mortality in low- and middle-income countries (LMICs). In 2022, approximately 660,000 women were diagnosed, and 350,000 died of cervical cancer worldwide, almost 90 percent of which were based in LMICs [1]. It is also the sixth most common cancer in women in the WHO Eastern Mediterranean Region and kills over 47,500 each year [2]. Cervical cancer is the second most common cancer amongst women aged 15–44 and the third most common cancer in general in Pakistan, with 5,008 new cases and 3,197 deaths per year. High-risk HPV 16 and 18 are linked to almost 88 percent of invasive cervical cancers in Pakistan [3].

The WHO Global Strategy for the Elimination of Cervical Cancer has the following ambitious targets: 90% HPV vaccination coverage among girls by age 15, 70% screening coverage, and 90% treatment coverage for cervical disease by 2030 [4]. Negative attitudes toward HPV vaccination are widely reported in studies from Pakistan: less than 3 percent of women have ever been vaccinated, and most present at advanced stages of the disease with low survival rates [5,6]. Misinformation, myths about fertility, and mistrust of health authorities, which have been enhanced by the previous opposition to polio and COVID-19 vaccination, further complicate efforts [7]. The global policy context highlights the urgency of action. Since 2016, Gavi, the Vaccine Alliance, has accelerated support for HPV vaccine introduction in eligible countries, enabling many South Asian countries to incorporate it into national immunization programs [8].

Effective introduction, however, requires more than vaccine procurement; it demands strategies that address the behavioral and social drivers of uptake in the Pakistani context. Despite growing global interest in addressing vaccine hesitancy, standardized tools remain scarce [9]. The WHO’s BeSD of vaccination framework and measurement tools includes both qualitative interview guides and a quantitative survey with a set of priority questions spanning cognitive, social, motivational, and practical domains that were rigorously developed through expert consultation, field-testing, cognitive interviews, and psychometric validation across multiple low- and middle-income countries (LMICs) [10]. BeSD tools have already been applied in contextually diverse settings of LMICs, like a mixed-methods study in Nigeria used BeSD to identify drivers of COVID-19 vaccination, finding that knowledge of access points, social network influence, and vaccine confidence were key determinants of uptake [11]. Regionally, the BeSD framework has also been used in Bangladesh, revealing how community beliefs, household costs, travel constraints, and health system factors influenced vaccine acceptance among women and zero-dose children [12]. However, this framework has not yet been systematically adapted or validated in Pakistan, a country with distinct cultural, religious, and social dynamics, particularly in relation to sexual and reproductive health and vaccine-related stigma.

Sociocultural, religious, and gender-related factors affecting vaccine uptake in Pakistan cannot be adequately described using traditional BeSD conceptions for several reasons [13,14]. First, religious and moral sensitivities regarding sexual and reproductive health directly influence perceptions of the HPV vaccination. Second, family structures are predominantly male-dominated; as a result, fathers and other elder males in the family usually have the final say on the health issues of women and children. Third, it remains a mistrust of immunization campaigns that have existed for a long time, such as polio and COVID-19, and is escalated by misinformation, conspiracy theories, fears of infertility, and foreign intrigues. Fourth, socially constructed sex-based norms limit information dissemination of reproductive health, especially to adolescents and girls. Recent evidence supports the emergence of context-specific cultural constructs during cross- cultural adaptation of the BeSD tool in the sociocultural context of Pakistan [15]. Moreover, national evidence during vaccine rollout has strongly highlighted the influence of gendered norms, normative beliefs, patriarchal decision making and the influence of religious and community leaders on vaccine-related decision making. Therefore, the meaning and measurement of all BeSD domains were altered by culturally embedded influences [13,15].

The meaning and measurement of BeSD constructs like “Thinking and Feeling”, “Social Processes,” “Motivation,” and “Practical Issues” can be changed by the national evidence that shows gendered barriers to vaccine access and decision autonomy, the pervasive influence of religious and community leaders on vaccine acceptance, and active digital misinformation through online platforms during Pakistan’s HPV rollout. The national and regional literature also indicates the above-mentioned factors and states that vaccination behavior in Pakistan is more group-based, hierarchical, and culturally dictated, in contrast to the individualistic or autonomous trends in other countries [13].

The current situation requires a culturally modified application of the BeSD model to prevent the false categorization of major drivers and achieve interpretive validity [16]. The background evidence shows that Pakistan is not just another LMIC having structural challenges and financial issues but also possesses a rich socio-cultural environment that needs an adaptation of BeSD in context to facilitate correct assessment and effective intervention.

Despite being an effective cervical cancer prevention method, HPV vaccination is not yet optimally utilized in most low- and middle-income countries. Community acceptance of new health interventions is strongly influenced by cultural alignment as well as structural and logistical problems [17]. The existing four domains of the BeSD framework, which are Thinking and Feeling, Social Processes, Motivation, and Practical Issues, address cognitive, social, attitudinal, and practical factors that affect the choices in vaccination. Although these domains are relevant, they do not fully account for the culturally embedded drivers shaping vaccine-related behaviors, especially in Pakistan’s diverse sociocultural context. The Cultural Integration domain is theoretically different from existing BeSD domains (e.g., Social Processes, Thinking & Feeling). It refers to structural and normative cultural pressures like male-controlled decision-making, religious moral systems, gender-based standards, and cultural taboos of reproductive health. These dimensions in our society impose limits in terms of who is allowed to make decisions, what can be discussed, and the suitability and acceptance of a culture of vaccination.

The purpose of this study was to develop a culturally adapted BeSD-HPV framework that is linguistically appropriate, contextually grounded, and culturally sensitive to evaluate the social, behavioral, and cultural factors influencing HPV vaccination in Pakistan. We acknowledge international gender-neutral guidance on HPV vaccination. Nevertheless, adolescent girls aged between 9–14 years were chosen in this study as the target population since the main aim was to facilitate the introduction of HPV vaccination in Pakistan. This approach is consistent with the existing national plans that are cervical cancer prevention-focused and girls-oriented. This is reflective of the gradual implementation model in many LMICs, whereby, at the initiation of the program, female-only vaccination is prioritized owing to cost, supply, and delivery constraints. Notably, the boys were not excluded conceptually in the analysis. Instead, they were not the main target of vaccination at this stage.

To provide a theoretically grounded basis for cultural adaptation, this study draws upon the Theory of Planned Behavior (TPB) as its conceptual foundation. TPB posits that health behaviors are primarily predicted by behavioral intention, which is shaped by three core determinants: Attitudes toward the behavior, Subjective Norms, and Perceived Behavioral Control [18].

To ensure the adapted framework captures the entire range of behavioral, social, and cultural factors influencing vaccine acceptance, the process aimed to preserve the conceptual integrity of the original four BeSD domains while incorporating locally emergent constructs into a new domain. The key focus was to develop a culturally adapted framework to guide evidence-based plans for introducing HPV vaccination in low-resource settings where cultural sensitivity is essential to the effectiveness of public health interventions.

### Theoretical foundations of the study

At the conceptual level, the BeSD framework is inherently aligned with the TPB. The TPB is the best-known socio-cognitive model used in psycho-social research to predict health-related behaviors and intentions [19]. Evidence from recent research also supports the use of TPB, which is widely employed to understand COVID-19 vaccination behavior [20].

Behavioral and social drivers of vaccine uptake in the cultural and geographic context in Pakistan’s can be explained in conjunction with the theory of planned behavior. Despite extensive research on the behavioral drivers of vaccination, the theory of behavior change has been applied only to a limited extent in the Pakistani context. Existing theories have not been adapted to inform an in-depth exploration of the behavioral and social drivers of vaccine uptake in Pakistan. In the absence of an existing theory to explain the social drivers of vaccination uptake, researchers have been unable to identify and focus efforts on developing measures of the social locus of control that underlie latent demand for vaccination.

BeSD domains align closely with TPB’s constructs: Thinking and Feeling correspond to Attitudes, Social Processes to Subjective Norms, and Practical Issues to Perceived Behavioral Control, while Motivation reflects Intention formation, the central construct in TPB. This integration provides robust evidence for understanding why individuals intend or fail to engage in preventive behavior, and for explaining the phenomena underlying hesitancy.

According to the Theory of Planned Activity, Attitudes towards behavior are influenced by underlying assumptions about the beliefs and values assigned to expected results. The BeSD framework reflects this construct through the Thinking and Feeling domain, which assesses perceived vaccine safety, disease risk and confidence in vaccine safety and efficacy. However, in the context of Pakistan, the culturally mediated attitudes influence the Thinking and Feeling domain of BeSD; these attitudes are inadequately captured by this domain, as they are treated as individual cognitive appraisals. The theory-driven adaptation amplifies the attitudinal domain by implicitly incorporating cultural nuances that reflect how HPV vaccination uptake is determined collectively as a cultural process influenced by gendered, patriarchal and hierarchical authorities rather than personally appraised individual decision.

In TPB, the perceived social pressure from significant others refers to Subjective Norms, which corresponds to Social Processes in the BeSD framework. This domain captures the influence of family norms, peer influence on approval, and disapproval of vaccination. However, in the context of Pakistan, the sociocultural influence extends beyond perceived approval and operates through deeply rooted cultural influences that function through gendered, patriarchal and hierarchical dynamics influencing the power dynamics for vaccine decision making. Vaccine uptake is strongly influenced by voices that hold moral legitimacy and regulate compliance and social sanctions.

Within TPB, Perceived Behavioral Control refers to the ease or difficulty of performing a behavior. The BeSD framework operates through a closely related construct, namely Practical Issues, which captures logistical barriers. However, in the context of Pakistan, structural access is deeply entangled with gendered mobility restrictions, limited female autonomy, economic dependencies, permission from male guardians, and even the gender of the vaccinator also influences vaccine decision-making.

The adaptation of the WHO Behavior Social Drivers’ framework in this study is theoretically aligned with the TPB, which postulates that health-related decision making is determined by behavior intentions which are shaped by Subjective Norms, Attitudes, and Perceived Behavior Control. Therefore, Cultural Integration serves as a cross-cutting analytical domain that shapes attitude (Thinking & Feeling), regulates social processes and norms (Social Processes) and logistic issues (Practical Issues) that limit translation of motivation into vaccine uptake.

Cultural Integration clarifies what is otherwise under-specified and implicit, instead of just replicating the existing BeSD domain. It provides clear, actionable insights to provide evidence-based strategies for culturally sensitive interventions to enhance uptake of the HPV vaccine in Pakistan’s sociocultural context, where cultural legitimacy is a prerequisite for vaccine acceptance. Therefore, Cultural Integration serves as a unique meta-level construct that captures the cultural, moral, and religious conditions that shape attitudes/Thinking and Feeling, subjective norms/Social Processes, and perceived behaviors/Practical Issues that influence Motivation.

## Methods

### Study design and setting

**Fig 1:**
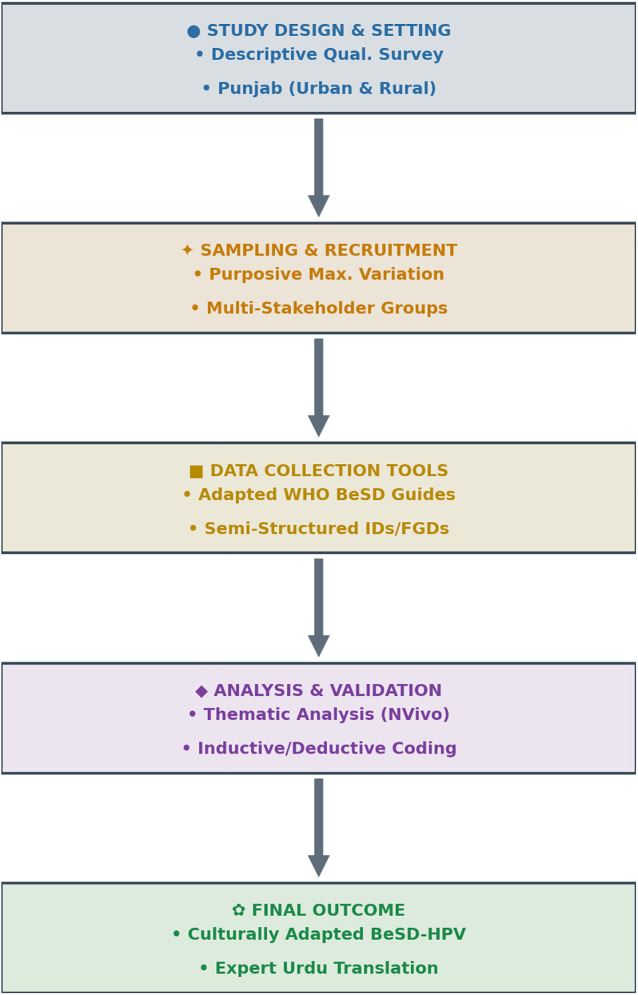
Methodological framework for a culturally adapted HPV vaccine acceptance study in Pakistan (Step 1: study design & setting, Step 2: sampling & recruitment, Step 3: data collection Tools, Step 4: analysis & validation, Step 5: final outcome).

The study employed descriptive qualitative design and was conducted from the end of February 2025 to August 2025 in District Rawalpindi and Islamabad Capital Territory in Northern Punjab and District Bahawalpur in Southern Punjab. On February 24th, 2025, the Ethical Review Board (ERB) of Health Services Academy granted ethical approval under IRB approval number (00015/HAS/PhD 2022). The study was conducted after taking informed written consent and in accordance with the policies and procedures established by the Graduate Research Management Council (GRMC) of Health Services Academy HSA Research and Ethical Committee and it adhered to the ethical guidelines outlined in the World Medical Association’s Declaration of Helsinki as revised in 2013 [21]. Detailed steps of the whole methodology are shown in Fig 1.

### Selection and description of participants

The purposive sampling technique was employed, considering that the study focuses on understanding the social and behavioral drivers using the WHO BeSD framework. This technique allows the purposeful selection of all relevant stakeholders, enabling in-depth exploration of factors influencing vaccine uptake and informing culturally relevant strategies for vaccine rollout. Study participants were recruited from District Rawalpindi and the Islamabad Capital Territory, in Northern Punjab, and from District Bahawalpur, in Southern Punjab. Prior to data collection, the participants were briefed on the study’s goals and objectives, and rapport was established. Data was collected after taking informed written consent. Participants who provided consent were recruited for the study. Participants included were adolescent girls (9–14 years), Parents/guardians of adolescent girls, Healthcare providers (vaccination staff, gynecologist, pediatrician, nurses, physicians), community workers (LHWs, LHSs & LHVs), teachers and school administrators, community leaders, and religious scholars from the Ministry of Religious Affairs. The districts were selected with a specific purpose to demonstrate differences in culture and health services in Punjab. Islamabad is the Capital Territory, and Rawalpindi comprises towns and urban areas with greater level of exposure to vaccination programs health services. Bahawalpur is a rural, more conservative place with its cultural regulations and more difficult accessibility to services. The reason behind this decision is to determine how the behavior of people, society and culture contributes to HPV vaccination in different environments, but not merely to obtain a statistically representative sample.

The selection of stakeholders was not random, as they are the ones who make HPV vaccination decisions and deliver it. The inclusion of diverse segments of society provided the option to obtain information on family, community, and health systems, which directly influence decisions about implementation and acceptance of the vaccine. Maximum variation sampling, rich contextual description, and stakeholder involvement in geographic, socioeconomic, and institutional settings were used to address transferability. These characteristics enable readers to determine the relevance of the findings in other LMICs and socio-culturally similar settings.

During the data collection period, HPV vaccination was not included in Pakistan’s national immunization program; therefore, none of the study participants (adolescent girls, parents, and community members) had been vaccinated against HPV. Thus, participants’ views are based on expected acceptance, conditional acceptance, or reluctance toward a new vaccine, instead of the experience related to the previous uptake.

### Data collection tool

Data were collected using semi-structured interview guides based on the WHO BeSD qualitative interview guides [10]. Original BeSD qualitative interview guides were used for data collection after adaptation. There are 4 qualitative interview guides for caregivers, health workers, community influencers, and program managers (attached as S1 File). The caregivers (parents/guardians) guide explored *Thinking and Feeling* (perceived safety, fertility concerns, trust), *Social Processes* (family and community influence), *Motivation* (intention to vaccinate, response to mandates), and P*ractical Issues* (access, cost, service preferences), with further probes to elicit culturally mediated decision-making and paternal authority. The healthcare provider discussed *Social Processes* (provider-community trust), *Motivation* (perceived responsibility to recommend HPV vaccination), and *Practical Issues* (delivery feasibility, staffing, outreach), and reflections on cultural and gender-related barriers experienced in practice. The community influencer and program manager guide (teachers, religious leaders, policymakers) stressed *Social Processes* and *Motivation* at the community and system levels, which include normative influence, legitimacy of vaccination, and implementation constraints, and also obtaining views on the widespread cultural norms influencing vaccine acceptance.

The core constructs of the BeSD framework were retained across all guides, and probes were tailored to stakeholder roles and local sensitivities to enable a systematic, yet flexible exploration of behavioral, social, and cultural motivations related to HPV vaccination. Interview guides were adapted to the local context following these steps:

**1. Familiarization with original BeSD**

The first step is to familiarize oneself with the original BeSD tools. This step involves detailed reviews conducted by a team of experts comprising researchers, Public Health experts, social sciences experts, behavioral scientists, anthropologists, and psychologists. During this stage, the following steps were involved.

- Each probe, item, and prompt was thoroughly examined for its conceptual intent.
- Potential areas of cultural disparity, like sensitive topics and terminology, were highlighted for modification.

**2. Contextual needs assessment**

Before making modifications, a desk review of the literature was conducted to identify potential barriers and facilitators to the HPV vaccine in the context of religious, sociocultural, gendered, and complex hierarchical dynamics.

This process helped to identify topics requiring modification in terms of rephrasing and contextual probing to enhance cultural relevance. Input from anthropologists, social scientists, and public health specialists familiar with the sociocultural and religious dynamics of the Pakistani setting guided the adaptation.

### Data collection and measurements

Data were collected through four focus group discussions and 45 in-depth interviews, each lasting 30–45 minutes, until data saturation was achieved. Interviews and Focus Group Discussions were conducted by KN, SM, and MN in the workplace. Participants were approached through personal contacts, emails, and phone calls. All interviews and focus group discussions were conducted face-to-face, except for three interviews with health system stakeholders, which were conducted virtually via Zoom. All interviews were conducted after informed consent was taken. There were no refusals or drop-outs. Anonymity and confidentiality were maintained throughout the process.

### Focus group discussions (FGDs)

- **Number and composition:** A total of four focus group discussion sessions were conducted. The first session was with mothers, the second with adolescent girls, the third with schoolteachers and staff, and the fourth session with program managers
- **Purpose:** FGDs were conducted to unfold shared norms, beliefs, identify behavioral and social factors, and examine culturally embedded perceptions influencing the vaccine uptake.
- **Moderation:** Each session was moderated by a trained facilitator with experience in qualitative research, well-versed in the local dialect, and fluent in Urdu. A scribe took detailed field notes, maintained group dynamics, and documented non-verbal cues. Each focus group session included 8–10 participants.

### In-depth interviews (IDIs)

- **Number and participants:** A total of 45 IDIs were conducted with diverse participants, including adolescent girls, their parents, LHWs, LHVs, and LHSs, healthcare providers, doctors, nurses, immunization staff, vaccinators, representatives from GAVI, WHO, and UNICEF, religious leaders, teachers, and school staff.
- **Purpose:** IDIs allowed probing of socio-culturally sensitive issues, exploration of personal beliefs, gendered norms, patriarchal influences, and in-depth understanding of culturally embedded facilitators and barriers to vaccination.

### Data analysis

We applied Braun and Clarke’s six-step thematic analysis using both inductive and deductive coding approaches in NVivo [22]. Deductive codes were aligned to the BeSD framework; inductive coding identified new constructs, leading to an additional “Cultural Integration” domain.

Using Braun and Clarke’s six-step thematic analysis approach, transcripts were independently coded by researchers KN, SM, and MN. A preliminary coding scheme was established through team discussions. The measure of coder agreement was the presence of regular debriefing meetings, during which codes and emergent themes were compared. Differences were resolved by consensus, with reference to the initial transcripts to ensure conformity with the participants’ meanings.

For translation, verbatim English interviews were transcribed into Urdu, and then bilingual researchers translated the material back into English. To provide an accurate and conceptually equivalent back-check, a second bilingual team member was used, who read and discussed the chosen transcripts with any discrepancies identified and resolved as a group. This allowed us to retain semantic as well as contextual meaning.

All interviews were audio-taped and recorded. Field Notes were also taken during and after the interviews and the focus group discussion. During analysis, an additional theme emerged inductively from participants’ narratives, capturing the community norms, patriarchal influence, gendered norms, social values, and religious beliefs.

**The complete de-identified interview transcripts and coding tree have been uploaded as** **S2** **and** **S3 Files**.

### Thematic analysis

At the beginning, four domains of the WHO BeSD, namely Thinking and Feeling, Social Processes, Motivation, and Practical Issues, were considered as a priori analytic scaffolds. In the first coding stage, all transcripts were initially looked at deductively to find out whether participants’ narratives could be meaningfully and conceptually placed within the established BeSD constructs and aligned with the corresponding TPB constructs. Coding decisions were determined by the theoretical purpose of each domain, not just by superficial thematic similarity. The use of this methodology made it clear that new findings are analyzed against the background of the original logic of behavior of the respective theoretical construct (e.g., cognition, social influence, intention, or access), and it avoided premature domain fragmentation.

Participant descriptions that could not be accommodated conceptually within any of the four domains, without disturbing their sense or overloading the definition of the domain, were designated to be inductively analyzed. This analytical field of study ensured that the original domains were not eroded by unjustified broadening.

After completing all deductive coding, the research team conducted a cross-domain analytical review to identify patterns in the data that explain domain insufficiency. For example, themes that did not logically fit into attitudes (Thinking and Feeling), subjective norms (Social Processes), intention (Motivation), or perceived behavioral control (Practical Issues) were considered conceptually non-aligned. Constructs that co-influenced beliefs, motivation, and behavior, reflecting an organizing role, but not falling under a single domain role, were believed to be cross-domain influence. The element based on long-standing sociocultural structures (such as patriarchy, religious authority, gender norms) but not on personal perceptions or situational constraints was counted as structural embeddedness. Themes that met the three criteria above were analyzed separately by contextual modifier within the existing domains.

### Conceptual framework

#### Conceptual framework of qualitative exploration and cultural adaptation.

Qualitative themes informed the item development and cultural adaptation of the existing BeSD framework.

**1. New items generation from qualitative interviews**

For each domain, potential new items were identified through:

- Reviewing the verbatim quotations that capture concepts not covered adequately in the existing four domains
- Mapping verbatim quotation to generate new items within the existing four domains
- Mapping these emergent concepts to a specific construct
- Explaining the rationale of each item
- Finally, translating into Urdu, in which Items were translated into Urdu by two independent translators, and the final version was synthesized by a linguistic expert, keeping in consideration conceptual and semantic equivalence.

**2. Construct derivation**

For each domain, thematic clusters were converted into measurable constructs. These constructs represented the conceptual basis for questionnaire items. These constructs in alignment with the theory of planned behaviour includes:

- *Thinking and Feeling*:(Attitude) perceived safety of HPV vaccine, concerns about side effects.
- *Social Processes (Subjective Norms)*: influence of peers, family, and community leaders.
- *Motivation (Intention)*: willingness to vaccinate, anticipated regret.
- *Practical Issues (Perceived Behaviour Control)*: accessibility of vaccination sites, cost considerations.

**3. Integration of emergent cultural constructs**

During the expert review process, the new domain “Cultural Integration” emerged.

This theme includes cultural norms, religious beliefs, and patriarchal influence on vaccination uptake. A new set of items was developed to explore how culturally mediated factors influence HPV vaccine acceptance.

**4. Mapping of qualitative findings to BeSD domains in alignment with the theory of planned behavior**

The cultural adaptation of the WHO BeSD framework was guided by the theoretical underpinnings of the theory of planned behavior. According to the theory of planned behavior, subjective norms, attitudes, and perceived behavior control influence health behavior, which in turn influences behavior intention and action. This data analysis process involved thematic synthesis and was conceptually grounded in integrating the WHO’s BeSD of Vaccination with the TPB. The integration demonstrates a deductive approach aligned with the theoretical framework and an inductive approach to incorporate contextual relevance related to HPV vaccination. The adaptation process in alignment with theoretical underpinnings ensured identification, integration, and interpretation of culturally embedded behavior social determinants influencing vaccination decision, with culture integration introduced as an emergent cross-cutting construct derived from qualitative inquiry.

### Item mapping and construct derivation

For each domain, the generation of new items and their mapping into relevant constructs were performed in the following steps:

#### Thinking and feeling (attitude).

This domain is consistent with the “Attitude” construct of TPB. This step involves understanding culturally embedded attitudes toward HPV vaccination. In-depth qualitative interviews and FGDs with parents and primary caregivers explored their beliefs and attitudes toward vaccine safety, efficacy, benefits, potential side effects, and fertility concerns. These attitudes were significantly influenced by religious perspectives, cultural norms, societal narratives, myths, and misinformation related to the HPV vaccine. In order to ensure attitude-related vaccine drivers of intention were culturally relevant, the Thinking and Feeling domain was expanded by adding 18 new items generated during qualitative interviews aligned with verbatim quotations capturing concepts inadequately represented in the original BeSD tool.

#### Social process (subjective norms).

Consistent with TPB’s subjective Norms, this domain highlights how vaccine-related behavior is influenced by societal expectations and cultural norms. Findings of in-depth interviews revealed how the vaccine decision is strongly influenced by community norms, intrahousehold decision-making authority, power dynamics, and relational autonomy. The Social Process domain was expanded by adding 15 new items derived from qualitative interviews, each aligned with verbatim quotations capturing the culturally embedded social processes influencing vaccine decision- making.

#### Practical issues (perceived behavioral control).

Perceived behavior control refers to the ability to perform behavioral in opposition to logistic barriers. Qualitative data in this study highlight several logistical barriers, including transportation, cost, vaccine availability, and accessibility. Several logistical barriers were also highlighted in the cultural context, including parental permission, informed consent, gender sensitive vaccine delivery, and the need for female vaccinators. The 17 new items were mapped to the domain of Practical Issues and refined to reflect structural and gendered realities influencing perceived behavioral control.

#### Motivation (behavioral intention).

Motivation represents a proximate determinant of vaccination behavior. It captures willingness, readiness, acceptance, and readiness to uptake the vaccine. Qualitative findings revealed that motivation was not an isolated influence; rather, it served as an interpretive lens, shaping participants’ perspectives across all domains of vaccine-related decision-making. By mapping the participants’ responses to the underlying construct, 8 new items were added to the domain of motivation.

### Cultural integration as an emergent cross-cutting domain

Using the above criteria, a consistent group of items was identified that runs throughout the stakeholder groups, such as a) household decision-making authority, which is patriarchal b) moral framing and religious legitimacy of vaccination, c) cultural silence on sexuality and reproductive health, and d) gendered standards of communication, consent, and service provision.

Analytically, this construct served as a fundamental determinant, acting as a bridge explaining how the four original BeSD domains integrate with the TPB construct in the Pakistani setting. For instance, cultural norms not only regulated social processes but also defined how individuals could engage socially. They did not merely affect motivation; they also described whether the motivation was socially allowed. In line with this, enlisting these constructs within the already established domains would have made their systemic contribution and explanatory value less clear.

Therefore, a separate domain, “Cultural Integration,” was introduced. It was thus not meant as an addition but rather as a structural extension required to maintain theoretical consistency while remaining contextually valid.

During the last stage of synthesis, all domains, both original and emergent, were mutually cross-referenced with participant quotations, derived constructs and suggested survey items. This step ensured conceptual consistency of the WHO BeSD framework with TPB, definite analytic demarcations between domains, and availability of raw qualitative data to the framework elements. The resulting BeSD-HPV model is therefore a culturally specific adaptation which retains the integrity of the original BeSD model but empirically generalizes it to incorporate sociocultural determinants that are fundamentally located in Pakistan and other such situations.

The coding tree is attached as a S3 File.

### Integrative framework adaptation

**Fig 2:**
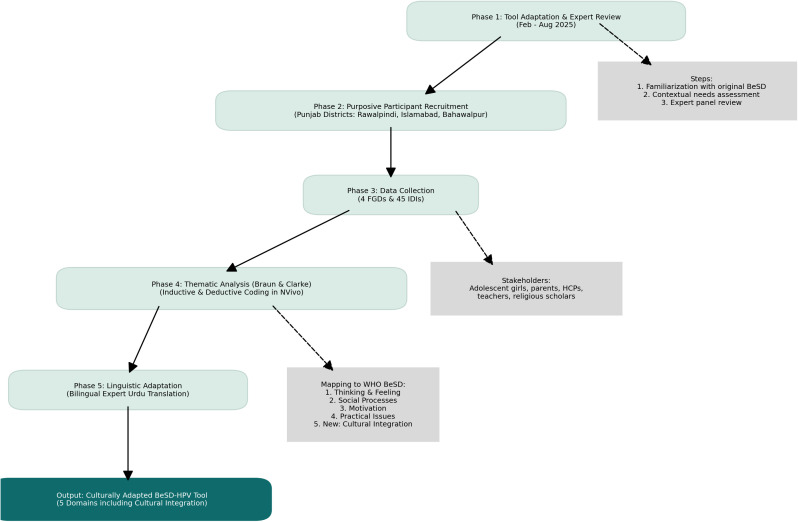
Conceptual framework for the qualitative exploration and cultural adaptation of the BeSD-HPV tool. The figure illustrates five phases: tool adaptation and expert review; purposive recruitment; qualitative data collection (FGDs and IDIs); thematic analysis and mapping to WHO BeSD domains; and linguistic adaptation. The process yielded a culturally adapted BeSD-HPV tool with five domains, including Cultural Integration.

By mapping qualitative findings onto TPB constructs and BeSD domains, the cultural adaptation process ensured conceptual coherence between empirical data and theory. In instances where culturally embedded determinants such as social authority and subjective norms were not fully captured by the original BeSD framework, behavior is shaped by collective, patriarchal, social, and religious norms. Fig 2 shows the conceptual framework for qualitative exploration and cultural adaptation

The final 70-item tool was developed through item mapping, construct derivation, and rationale; the corresponding Urdu item is attached as S4 File.

### Theoretical and methodological implications

Aligning TPB with BeSD during adaptation enhances the theoretical validity of the adapted framework by establishing that cultural determinants correspond to deeply ingrained behavioral phenomena. This integration positions the adapted BeSD framework not only as a descriptive tool but also as a theoretically grounded model for developing culturally responsive HPV vaccination interventions in Pakistan and comparable sociocultural settings.

The construction of the culturally adapted BeSD-HPV framework was not developed through a superficial exploration or de novo structure-building method, but through a theory-preserving, step-by-step analytic synthesis method. The analytic procedure was also designed to maintain the conceptual soundness of the original WHO BeSD framework and to empirically test its suitability in the Pakistani sociocultural environment.

**5. Finalization and documentation**

The final adapted preliminary version of BeSD HPV comprised:

- Items aligned to construct mapping to Original WHO BeSD domains in alignment with TPB’s construct (Thinking and Feeling (Attitudes), Social Processes (Subjective Norms), Motivation (Intention), Practical Issues (Perceived Behavior Control)
- New Items aligned to construct mapping to the newly added domain *“Cultural Integration*

To ensure transparency and reproducibility, all adaptation decisions, rationale, and justifications were documented.

Supplementary Tables (S1–S5 Tables) mapped each survey item within each BeSD domain, which were then systematically mapped to specific constructs based on subthemes that emerged during thematic analysis. Detailed item mapping for each domain is provided in Supplementary Tables (S1–S5 Tables).

**Fig 3:**
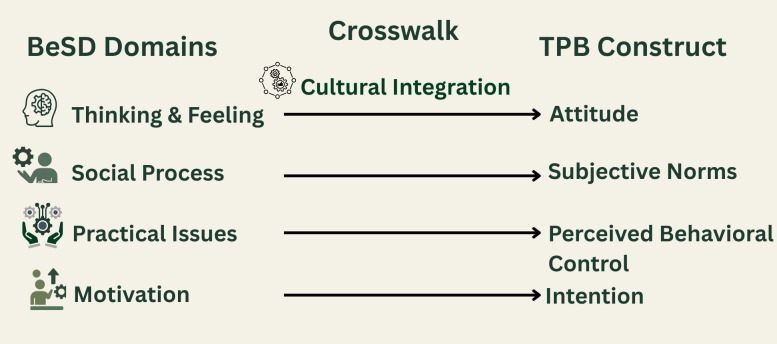
Cultural adaptation process aligning BeSD domains with the Theory of Planned Behavior (TPB). This framework illustrates how TPB constructs correspond to BeSD domains: Attitude aligns with Thinking and Feeling; Subjective Norms with Social Processes; Perceived Behavioral Control with Practical Issues; and Intention with Motivation. Cultural Integration functions as a cross-cutting domain shaping all components.

Each construct is then aligned with the survey item, linking it to key quotes from participants and providing a detailed rationale. Finally, linguistic adaptation is ensured by translating it into Urdu with the help of a bilingual language expert. able cell colors are aligned with WHO color coding and indicative of the respective domains (Thinking and Feeling, Social Processes, Motivation, Practical Issues, and emergent new theme, Cultural Integration. Fig 3 illustrates the theoretical basis for aligning BeSD domains with TPB constructs.

To make the study results rigorous and credible, the research team was actively involved in the process of reflexivity and used the Four-Dimensions Criteria of credibility, transferability, dependability, and confirmability developed by Lincoln and Guba [23].

**Credibility** was facilitated through FGDs, intensive interviews, and data triangulation. The deep insight was possible with the use of both IDIs and FGDs. During interviews, member checking was an informal process used to verify participants’ statements and clarify their meanings. Moreover, the interviewers were trained in the qualitative approach and practiced cultural sensitivity to foster an atmosphere of openness and trust.

The **transferability** was supported by the intentional selection of a diverse sample that consisted of participants with different educational backgrounds, socioeconomic statuses, and geographic locations. The participants’ responses and the settings within local health services were also described in detail and in context, which would allow readers and future researchers to assess the generalizability of the findings to other settings.

The question of **dependability** was addressed through an open, logical approach to data collection and analysis. Thematic refinement included an audit trail tracked through elements such as interview guides, transcription protocols, coding structures, and decision logs. To maintain analytic consistency, the research team held debriefing sessions to discuss new trends and address inconsistencies.

The **confirmability** was ensured by being in a reflexive position throughout the study. The authors prepared subjective reflections and subjective memorandums, which they recorded in order to bracket their own assumptions and minimize bias. Peer debriefing and team discussions also helped to suggest an impartial interpretation. The research ensured a strong relationship between the data and its interpretation by grounding the themes in rich, participant-derived quotations, and the results correlated with the BeSD framework. Collectively, these strategies enhanced the study’s methodological rigor and reinforced the authenticity, reliability, and applicability of the findings on HPV vaccine hesitancy and acceptance within the Pakistani context. This study was reported in accordance with the COREQ (Consolidated Criteria for Reporting Qualitative Research) reporting guidelines [24].

## Discussion

This study aimed to explore behavioral and social drivers of HPV vaccine hesitancy in Pakistan, and to use these insights to adapt the WHO BeSD framework into a context-specific tool. We have assessed all four domains of thinking & feeling, social issues, motivation & practical barriers mentioned in the BeSD framework in alignment with four constructs of TPB, namely Attitudes, Subjective Norms, Intention, and Perceived Behavior Control, along with an explanation of the emergent domain “Cultural Integration”. A concise overview of the principal themes that have been discerned throughout the process is:

**(i) Thinking and Feeling (Attitudes)**

In accordance with TPB’s attitudinal construct, this domain mainly addresses participants’ concerns about vaccine safety, perceived benefits, and side effects. Findings strongly demonstrate that participants’ perceptions of HPV vaccination are not limited to generic safety concerns but extend into a culturally mediated belief system.

**(ii) Social Processes (Subjective norms)**

Consistent with subjective norms of TPB, this domain highlights how vaccine behaviors are influenced by community norms and hierarchical decision-making systems. Results indicate that vaccine decision-making is not just perceived approval, but it is dependent on socially sanctioned approvals

**(iii) Motivation (Behaviour Intention)**

In line with TPB, motivation served as the proximal factor for vaccination intention. The result strongly highlights that motivation is not individually governed, but rather a socially constructed phenomenon influenced by patriarchal hierarchies.

**(iv) Practical Issues (Perceived Behaviour Control)**

In line with the perceived behavioral control of TPB, this domain emphasized that logistical constraints are also influenced by sociocultural hindrances, such as the need for gender-sensitive vaccine delivery, reluctance in getting vaccinated among male vaccinators, and parental permission. These findings explain the concept of “access” as a socially mediated phenomenon rather than just a logistic constraint.

**(v) Emergence of Cultural Integration as a Cross-Cutting Domain**

Rather than deviating from the original BeSD domains, the introduction of “Cultural Integration” serves as a cross-cutting domain that shapes how other determinants, such as cognition, social processes, practical barriers, and intention, are expressed.

These key results would make each domain more useful in comprehending HPV vaccine uptake in the Pakistani setting.

The application of the BeSD tool in a low-resource setting such as Pakistan provides a valuable opportunity to study the constraints of applying public health frameworks and to highlight the necessity of culturally competent methodologies in global health. We have interviewed all stakeholders, including policy makers, doctors, nurses, parents, and girls aged 9–14 years, to assess their perspective on it. Although the initial framework of the BeSD served as a good starting point, our findings in Pakistan indicate that the conceptualization of cultural barriers lacked a specific domain. This is most relevant in cases where health choices are not simply individual but are deeply rooted in intricate social, religious, and paternal backgrounds. The raw information gathered via qualitative interviews emphasizes that vaccine hesitancy cannot be addressed as an intellectual deficit that can be addressed by informational efforts, but is rather a complex phenomenon rooted in a historical and social culture of distrust [25]. For instance, the view of free vaccines as a built-in suspicion reflects a lack of trust in the state and in foreign forces, which was likely shaped by past experiences with health programs, social media, and the broader political landscape [26]. Similarly, the fear of the HPV vaccine being a tool for birth control, while seemingly unproven, is a strong cultural opinion that demands a delicate, context-specific response, rather than a simple refusal of the claim [27,28]. These insights explain that effective public health interventions in such settings must be built on a deep understanding & thinking of local knowledge and cultural logic, rather than on a systematized, top-down approach.

The expanded framework, which now includes the Cultural Integration domain, would offer a more comprehensive tool for public health practitioners and providers. The presumptive inclusion of items related to the role of men or fathers in family health decisions and the influence of religious leaders has helped capture key drivers of hesitancy that would otherwise remain unaddressed [29]. The data reveal that a mother’s or daughter’s readiness to vaccinate is often unanticipated on the approval of male family members, highlighting the boundaries of women-focused health campaigns. A gender-sensitive approach, therefore, must be rebuilt to include plans and policies for engaging male stakeholders, potentially by framing the HPV vaccine as a precautionary measure for the entire family’s honor and future well-being, a concept that resonates deeply with the cultural context. Moreover, concerns about the legality of vaccine components and public debates on reproductive health underscore the need for community-based participatory research methods. By creating communication materials with the help of local leaders, the messaging can be disseminated with cultural authenticity and delivered through trustworthy, community-certified channels [30].

The implications of this study extend beyond communication to the practical application and long-term sustainability of HPV vaccination programs. The culturally adapted tool recognized specific delivery challenges, such as a preference for female vaccinators and the potential for stigma associated with public, gender-specific health campaigns. These findings provide a guide for program design, indicating that vaccination services should be integrated into existing, trusted healthcare platforms where female vaccinators are already present. The hesitation & uncertainty linked to gender-specific campaigns further emphasize the need to re-evaluate how the HPV vaccine is being introduced into vaccination programs. Instead of merely focusing on cervical cancer prevention, which may be a culturally sensitive and inappropriate topic, a broader public health narrative around general adolescent health and disease prevention could be more valuable and effective.

Previous studies on the BeSD framework have mainly focused on measuring the behavioral and social drivers of vaccination across 4 predefined domains. Studies conducted in low-income countries, including Bangladesh, have shown that the BeSD framework is robust in identifying behavioral social drivers of vaccination [31]. Similarly, a study conducted in Nigeria used the BeSD framework to assess the social drivers of COVID-19 vaccination, showing that all 4 domains of the framework significantly influenced COVID-19 vaccine uptake [11]. The current study goes beyond item-level adaptation. While our study findings are consistent with prior studies, we observe that certain findings emerging from qualitative interviews do not align with the existing 4 domains without conceptual alteration. Specifically, emerging themes related to religious legitimacy, patriarchal power, moral framing of sexuality, and gender norms operate as unifying structures influencing multiple domains simultaneously. In contrast to previous studies on cultural adaptation, which place culturally embedded expressions within existing 4 domains, our rigorous analytic process treats these culturally mediated influences as a cross-cutting phenomenon influencing all existing domains [32]. This domain represents conceptual refinement that emerged through context-specific qualitative inquiry and represents context-driven extension derived through a rigorous, systematic analytical process.

Another salient feature is theoretical integration with the Theory of Planned Behavior (TPB). Previous research on BeSD focused on operationalizing 4 domains, whereas our study distinguished itself by mapping qualitative findings onto TPB constructs (Attitudes, Subjective Norms, Perceived Behavioral Control, and Intention). This dual theoretical foundation allowed us to evaluate conceptual congruence and highlight areas of theoretical insufficiency. The findings of this study strongly support that these constructs did not operate at the individual level in a patriarchal collectivist society; rather, they are influenced by cultural nuance as a cross- cutting phenomenon determining whether attitudes can be expressed, norms can be negotiated, whether behavior controls are permissible, and how motivation is socially validated. Thus, integrating TPB into the adaptation process enhances theoretical validity and emphasizes the need to account for sociocultural factors that influence vaccine decision-making. This process also ensures that the cultural adaptation process was empirically grounded rather than conceptually imposed.

We suggest that integrating these culturally sensitive adaptations into the BeSD tool may improve its effectiveness in achieving sustainable vaccination practice in similar resource-constrained and culturally complex settings. However, we should take this fact into consideration that the Cultural Integration domain is exploratory and inductively based on qualitative data and has not yet been psychometrically or predictively validated. The present study’s findings provide hypothesis-generating information and evidence of non-irrevocable policy change. The modified version of the BeSD-HPV tool and the Cultural Integration domain need to be quantitatively tested in the future, including item deletion, reliability, construct validity, and a discussion of their explanatory value relative to the current BeSD domains. We further suggest that future research should enhance the prediction of HPV vaccine uptake and clarify the intervention effect by including this domain.

In a nutshell, compared with previous research on the BeSD framework, this study 1. maintains empirical support for the original four BeSD domains 2. Highlights the need for contextual expansion in patriarchal sociocultural milieu 3. Strengthen theoretical foundation through alignment with TPB 4. Proposes a cross-cutting domain to capture culturally embedded constructs

### Strengths and limitations

The study draws strength from the inclusion of diverse stakeholder perspectives including adolescents, parents, healthcare providers, teachers, and community and religious leaders, ensuring a holistic understanding of HPV vaccine uptake. The use of the WHO BeSD framework further enhances methodological rigor and aligns the study with global standards, while the integration of cultural constructs adds contextual relevance and practical applicability of the findings.

The present study has several limitations that must be acknowledged. Firstly, HPV vaccination status was not considered in sampling or analysis because, at the start of the study, HPV vaccination was not included in the Pakistani government’s vaccination schedule. Secondly, the potential for social desirability bias during interviews, particularly when addressing sensitive cultural and religious issues. It is quite possible that respondents may have provided answers acceptable within society rather than expressing their full reservations. Thirdly, as the study was conducted in only two districts of Punjab Province, the perceptions captured may not fully represent the views of participants from other provinces of Pakistan, which have different sociocultural and ethnic backgrounds and health-system environments. Fourthly, the present study relied on purposive sampling, which is appropriate for in-depth qualitative investigation and offers an opportunity to include essential stakeholders, but it limits representativeness and does not allow generalization of the results to the broader population.

Despite these shortcomings, the research provides valuable, context-based insights that are invaluable for culturally adapting the BeSD framework and for informing HPV vaccine introduction efforts in similar settings.

## Conclusion

In conclusion, our work on the culturally adapted BeSD tool in Pakistan makes a remarkable contribution to understanding vaccine hesitancy by providing a methodological blueprint for contextualizing global health frameworks. It underscores that impactful progress in HPV vaccine introduction in low-resource settings requires a paradigm shift from a global, biomedical model to a culturally informed, community-engaged, and responsive model that addresses local social determinants of health. The incorporation of a new *“Cultural Integration”* domain offers an additional perspective that may help address the non-traditional drivers of vaccine hesitancy, paving the way for more effective, sustainable, and equitable public health interventions, particularly in LMICs. This study provides evidence for the cultural adaptation of the BeSD framework to develop a context-specific and linguistically appropriate tool for collecting baseline data on sociocultural factors influencing HPV vaccine uptake. It also informs the development of strategies for the planned introduction of the HPV vaccine in Pakistan and provides a foundation for future implementation research. However, future quantitative validation of the adapted tool is needed.

## Supporting information

S1 File Qualitative interview guides for caregivers, healthcare providers, community influencers, and program managers (adapted from WHO BeSD). (DOCX)

S2 File De-identified interview transcripts. (DOCX)

S3 File Coding tree from thematic analysis. (DOCX)

S4 File Final 70-item BeSD-HPV tool (English and Urdu versions). (DOCX)

S1 Table Thinking and Feeling Domain – item mapping, quotes, and rationale. (DOCX)

S2 Table Motivation Domain – item mapping, quotes, and rationale. (DOCX)

S3 Table Social Processes Domain – item mapping, quotes, and rationale. (DOCX)

S4 Table Practical Issues – item mapping, quotes, and rationale. (DOCX)

S5 Table Cultural Integration – item mapping, quotes, and rationale. (DOCX)
