# Supplementary material for: From global to local: Developing a context-specific BeSD-HPV tool through cultural and linguistic adaptation in Pakistan
Source: PLoS One. 2026 Jun 15;21(6):e0350162. doi: 10.1371/journal.pone.0350162 (PMC13268181; doi:10.1371/journal.pone.0350162)
Supplement: S1 File — (DOCX) [file pone.0350162.s001.docx]

**CHILDHOOD VACCINATION INTERVIEW GUIDES – FOR COMMUNITY INFLUENCERS (who promote vaccination for children under 5)**

| **Question / [Instruction]** | **Rationale** |
| --- | --- |
| *Introduction: Hello, I am [INTERVIEWER’S NAME] with [INSTITUTION OR ORGANIZATION NAME]. We are interviewing people to help improve vaccination services in [NAME OF COUNTRY].*    *The interview is expected to take __ minutes. Your participation is completely voluntary and anonymous. The answers you give will be completely confidential. If you do not want to answer a question or wish to stop the interview, just let me know. Would you be willing to take part in an interview with me? [if audio recording the interview] Would you be happy for me to record our conversation?* | - Clear introduction to ensure true informed consent for participation is obtained before proceeding |
| **Tell me a little about yourself and your role here in the community** Probe:   - To what extent does your work involve immunization? - Can you tell me more about that? - Who do you work with to do that work? | - Warm-up question - Enables understanding of the participant’s role in the community - Understanding of the breadth of the participant’s responsibilities |
| **Can you take me through the process you follow when you work in a community?**  Probe:   - [Note this probe is for participants who work with families] When you visit a family, o What do you talk about? o What information can you not leave without saying?   o Do you follow up with the families afterward? How do you do that?   - [Note: this question is for participants who work with other people and organizations, use as appropriate for the participant] o How do you help the front-line health workers in working with families? o How do you help with routine immunization? | - To understand the details of the participant’s immunization-related activities - [Note: some participants may work directly with families, others work with NGO’s and other agencies. The suggested probe questions should be adjusted to suit the participant’s setting and role] |
| **What do you find works in helping families stay up to date with their children’s immunizations?**  Probe:  - What helps them not miss doses or appointments? [Note: this is to probe for practical issues] - What helps those who are hesitant about getting their children vaccinated? | - This question is designed to find out what, in the participant’s experience, helps keep families up to date with immunizations for their children - [Note: the question is intentionally broad and open ended so that all possible answers are gathered]. |
| **What makes it difficult for families stay up to date with immunization?** Probe:   - Can you give some examples of reasons people give when their child has fallen behind the vaccination schedule? - Can you give some examples of reasons that people give for refusing vaccines for their children? - Are you able to overcome these challenges? How? | - This question is designed to help identify and understand difficulties the participant sees for families to keep up to date with vaccinations in their community. |
| **If you had the chance, what would you do to improve immunization services in your area?** | - Aim to identify any other issues or suggestions not identified in the previous line of questioning - Closing question |

**CHILDHOOD VACCINATION INTERVIEW GUIDES – FOR PROGRAMME MANAGERS**

| **Question / [Instruction]** | **Rationale** |
| --- | --- |
| *Introduction: Hello, I am [INTERVIEWER’S NAME] with [INSTITUTION OR ORGANIZATION NAME]. We are interviewing people to help improve vaccination services in [NAME OF COUNTRY]. We’re seeking input from people like you who know the processes and the work well. Your views are crucial and very valuable.*    *The interview is expected to take __ minutes. Your participation is completely voluntary and anonymous. The answers you give will be completely confidential. If you do not want to answer a question or wish to stop the interview, just let me know. Would you be willing to take part in an interview with me? [if audio recording the interview] Would you be happy for me to record our conversation?* | - Clear introduction to ensure true informed consent for participation is obtained before proceeding |
| **Tell me a little about yourself and your current role** Probe:   - To what extent does your work involve childhood immunization? - What kinds of immunization-related activities are you responsible for (e.g. surveillance, campaigns, communications etc) - Can you tell me more about those? | - Warm-up question - Enables understanding of the participant’s overall current role - Understanding of the breadth of the participant’s responsibilities - Understanding the extent of their immunization related activities and what those entail. |
| **What makes the provision of childhood immunization a success in your area?**  Probe:  - Are there specific examples you can describe? | - This question is designed to find out what, in the participant’s experience, helps keep families up to date with immunizations for their children - [Note: the question is intentionally broad and open-ended so that all possible answers are gathered]. |
| **What do you think are the difficulties when it comes to providing childhood immunization in your area?**  Probe:   - Do you face difficulties with children falling behind the vaccination schedule in your area? Can you describe them? - Do you face difficulties with parents refusing vaccines for their children? - Are you able to overcome these challenges? How? | - This question is designed to help identify and understand difficulties the participant sees for families to keep up to date with vaccinations in their jurisdiction. |
| **If you had the chance, what would you do to improve the childhood immunization situation in your area?** | - Aim to identify any other issues or suggestions not identified in the previous line of questioning - Closing question |

**CHILDHOOD VACCINATION INTERVIEW GUIDES – FOR HEALTH WORKERS**

| **Question / [Instruction]** | **Rationale** |
| --- | --- |
| *Introduction: Hello, I am [INTERVIEWER’S NAME] with [INSTITUTION OR ORGANIZATION NAME]. We are interviewing people to help improve vaccination services in [NAME OF COUNTRY].*    *The interview is expected to take __ minutes. Your participation is completely voluntary and anonymous. The answers you give will be completely confidential. If you do not want to answer a question or wish to stop the interview, just let me know. Would you be willing to take part in an interview with me? [if audio recording the interview] Would you be happy for me to record our conversation?* | - Clear introduction to ensure true informed consent for participation is obtained before proceeding |
| **Tell me a little about yourself and what you do** Probe:   - What are you responsible for? - How many days do you work in this role? | - Warm-up question - Enables understanding of the participant’s professional role - Understanding of the breadth of the participant’s responsibilities |
| **Question / [Instruction]** | **Rationale** |
| - Where do you perform your duties? | - Understanding how many days per week the participant works and where they are situated physically (e.g. do they work at multiple sites) |
| **To what extent does your role involve immunisation?**    Probe:   - What parts of your job involve immunisation? - Can you tell me more about that? | - To understand how much of the participant’s role is immunisation related - To understand in some detail what those immunisation-related responsibilities are |
| **I’d like to understand the process you follow to immunize a child, starting from the very beginning.**  Probe:   - Does it involve work for you even before the family arrives at the center for vaccination? - Can you summarize the procedure of immunization in around 5 steps starting once a family arrives at the center for vaccination? [note: adjust this question for non-clinic settings if required] - Are there are follow ups or steps involved once they leave the center? [note: other probes such on going door-to-door, systems of recording vaccinations, making vaccination cards and so on could be added, as required.] | - This question is for workers who administer immunizations to children. - Aim is to understand the work processes followed by the participant   - may shed light on logistic or practical barriers they may encounter when delivering immunization services   - may shed light on facilitators that could be applied elsewhere - [Note: The wording of this question is currently framed for a health worker in a clinic-type setting. The wording will have to be adjusted for the approach used in the setting being researched, for example, outreach or mobile vaccination services] |
| **What do you find works in helping families stay up to date with immunisation?** Probe:   - What helps them not miss doses or appointments? [Note: this is to probe for practical issues] - What helps those who are hesitant about getting their children vaccinated? | - This question is designed to find out what, in the participant’s experience, helps keep families up to date with immunizations for their children - [Note: the question is intentionally broad and open-ended so that all possible answers are gathered]. |
| **What do you find difficult when it comes to helping families stay up to date with immunisation?** Probe:   - Which part of the process you described before do you find the hardest to complete? Why is that? - Can you give some examples of reasons people give when their child has fallen behind the vaccination schedule? - Can you give some examples of reasons that people give for refusing vaccines for their children? | - This question is designed to help identify and understand difficulties the participant faces in helping families to keep up to date with vaccinations. - [Note: The suggested probes are to help separate differences between difficulties in the process they describe above, and difficulties they think families experience.] |
| **If you had the chance, what would you do to improve immunisation services in your area?** | - Aim to identify any other issues or suggestions not identified in the previous line of questioning - Closing question |

| **FOCUS GROUP DISCUSSION GUIDE FOR LHWs/LHVs**  **Question** | |
| --- | --- |
| 1 | Can you tell me about your role?   - What parts of your job involve immunization? - Do you have any role in the HPV vaccine rollout (have you heard about this before)? - Can you tell me more about that? |
| 2 | Can you tell me about how the NEW vaccine is being rolled out in your community (Previous experience)?   - What is the procedure in schools/health centres? - Who administers the vaccine? - How does the catch-up program for girls aged 11-17 years’ work? - Is there a specific vaccination day at schools? Tell me more. - Can you tell me about the consent process? - What information is given to parents and girls? |
| 3 | How do you think parents in the community feel about getting the new ( ) vaccine for their daughters?   - What kinds of things have you heard from parents who already had their daughters vaccinated against Routine Immunization? - What have you heard from parents who are not sure or who don’t want the vaccine for their daughter? |
| 4 | How do you or your colleagues feel about administering the vaccine?   - How do you feel about promoting the HPV vaccine? - Do you think this vaccine is different from other vaccines or not really? Tell me about that - Would you get the HPV vaccine for your daughters / granddaughters? Tell me more |
| 5 | In your current role, can you tell me about the information you received or would like to receive about the HPV vaccine?   - How would you describe what you received and how did you feel about it? - What kind or information do you need to know? - How much information would you like and when? - Who is the best person/ organization to provide this information? - What’s the best format to get this information? |
| 6 | What information about the HPV vaccine is important for the community to know?   - For parents? For teachers and schools? For girls? - Who is the best person/ organization to provide this information? - What’s the best format to get this information? |
| 7 | Can you tell me about how the NEW vaccine is being rolled out in your community? (Addressing this age cohort. Take example of recent ones , the campaign for typhoid, up to 15 years. the campaign for MR, for measles, rubella. up to 15 years at that time. context of HPV in terms of unisex).   - What is the procedure in schools/health centres? - Who administers the vaccine? - How does the catch-up program for girls aged 11-17 years’ work? - Is there a specific vaccination day at schools? Tell me more. - Can you tell me about the consent process? - What information is given to parents and girls? |
| 8 | What makes the provision of childhood immunisation a success in your community?   - Are there specific examples you can describe? |
| 9 | For childhood vaccines, what do you find works in helping families stay up to date with immunisation?   - What helps them not miss doses or appointments? - What helps those who are hesitant about getting their children vaccinated? |
| 10 | What do you think are the difficulties when it comes to providing childhood immunisation in your community?   - What impact do you think the COVID-19 pandemic and vaccine rollout has had on trust and confidence in vaccines, if any? - Do you face difficulties with children falling behind the vaccination schedule in your area? Can you describe those difficulties? - Do you face difficulties with parents refusing vaccines for their children? - Are you able to overcome these challenges? How? |
| 11 | If you had the chance, what would you do to improve immunisation services in your community |
| 12 | Do you have anything else that you would like to add? |

**BeSD Working group on data for vaccination acceptance and demand**

**Key Informant Interview Schedule**

In your role we feel you would be able to give us some insight into what kinds of measurement tools you might need, and how they might be deployed in your country/region.

1. **What is your role?**
2. **How does your region/country use data to inform immunization programme planning?**
3. **Does your region/country undertake surveys to understand the factors that contribute to vaccination uptake?**
   1. If so, how was this done (sampling, deployment, analysis, use of results)
4. **What do you most want to know about in relation to immunisation in your country/region/what is not yet known that would help you prioritise support/activities in your region/country?**
   1. [probe reasons for answers - can you tell me more about that? Look for alternatives]
5. **(Describe proposed BeSD tool structure and process) What do you think about having these tools available?**
6. **Can you foresee problems or barriers to using tools like this in your country/region?**
   1. [probe, example, perceptions of how it’s used by others, motivation, capacity, resourcing, time.
7. **How do you think your country/region might use such a package?**
8. **Who do you envisage would be conducting the qualitative interviews (local staff, consultants, students?)**
   1. [probe beyond a list – what is it that makes these things strengths?]
9. **What do you think would help get this up and running in your country/region?**
10. **What should the WHO consider doing to make sure it keeps getting used?**
11. **Do you have any other thoughts?**

**Interview Guide for Teachers**

**1. Icebreaker questions**

The moderator puts the participants at ease and uses this question to illustrate that there is no right or wrong answers.

A good icebreaker question may invite group members to use their imaginations or talk about their preferences.

**2. Engagement questions** The moderator opens the topic of the research, starting with an easy question that will engage the participants’ interest and get them actively involved and the discussion flowing.

→ “Let’s talk about some experiences you have had with vaccines and vaccination. Who would like to share?”

→ “Do you know anyone who has had a vaccine-preventable disease? Can you share that person’s story?”

**3. Exploration questions** This is the core part of the focus group discussion. The moderator explores all the research objectives for this group and comes up with questions which will ensure that they can be achieved. The order is not important, as long as all research objectives are covered.

**Knowledge**

→ “What vaccines do people routinely receive here in (country)?”

→ “Can you explain how vaccination works?”

→ “Have you heard of any new vaccines and, if so, what have you heard?”

→ Have you ever heard about HPV Vaccine and, if so, what have you heard?”

**Information sources**

→ “If you were looking for information related to vaccines, who would you turn to?”

→ “Where would you go to look it up?”

→ “What sources related to vaccines do you think are most trustworthy, and why?”

**Barriers**

→ “What do you think about vaccination?”

→ “What is your experience with vaccination?”

→ “What have you heard about other people’s experiences with vaccination?”

→ “Some people do not want to vaccinate their children. Why do you think that is?”

**Enablers**

→ “If a new vaccine were introduced into the country, what would you want to know about the vaccine?”

→ “What do you think people need to know in order to trust vaccination?”

**Communication channels**

“What would be the best way to inform people in this country about a new vaccine?”

→ “What would be the best or easiest way for you to learn more about vaccination?

**Messages**

“What would you say to a friend who asked you for advice about vaccination?”

→ “Can you think of a story, photo, news story or health promotion campaign that had a lasting effect on you? Please explain.”

**4. Exit questions**

The moderator checks to see if anything was missed in the discussion and invites participants to give any last ideas. It is important to include these questions, because sometimes people have a special concern or idea that they have not yet had a chance to bring up.

→ “We still haven’t heard very much about (topic).

Does anyone have any thoughts about that?”

→ “This has been a very good discussion! Is there

anything else anyone would like to say about

vaccines and vaccination?”

**Qualitative Interview Guide (Religious Affairs)**

Aim

To understand multifaceted sociocultural and contextual factors influencing the uptake of HPV vaccine to support the launch of HPV Vaccination in Pakistan

Objectives

1. To assess the current level of knowledge, belief, and factors associated with the acceptability of HPV vaccination among potential vaccine recipients and their parents/caregivers.
2. To explore stakeholders’ perspectives on the launch of HPV vaccination, considering the dynamics of the local population in the resource-constrained country, Pakistan.
3. To identify the social and behavioral factors that influence HPV vaccination acceptance and hesitancy within a local community (Punjab).

BeSD domain:

General

*Introduction: Hello, I am Dr…. working in the*

*The interview is expected to take __ minutes. Your participation is completely voluntary and anonymous. The answers you give will be completely confidential. If you do not want to answer a question or wish to stop the interview, just let me know. Would you be willing to take part in an interview with me? [if audio recording the interview] Would you be happy for me to record our conversation?*

Q1. Tell me a little about yourself and your job description

Probe: For how long have you been employed here? What is the nature of duties, roles & responsibilities

Let’s talk about some experiences you have had with vaccines and vaccination.

HPV

→ Have you heard about cervical cancer? What have you heard?

→ Do you know anyone who has had cervical cancer?

Can you share their story with us?

→ How serious do you think cervical cancer is?

Moderator explains:

Cervical cancer is caused by a very common sexually transmitted virus, called human papilloma virus, or HPV. A vaccine against HPV is used in many countries to protect their populations.

Q2. Have you heard about the Human Papillomavirus (HPV) vaccine before?

If yes: Where did you first hear about the HPV vaccine (e.g., media, school, health workers)? What do you know about it?

Q3. Do you know what the HPV vaccine is and what it protects against?

WHO recommends giving this vaccine to girls aged 9-16 to protect them against cervical cancer when they are adults. So Pakistan plans to begin introducing it to girls of 9-16 years of age by the last quarter of 2025.

Q 4. Pakistan is going to introduce HPV Vaccination for girls between 9 to 16 years of age. How do you feel about the introduction of HPV vaccination in Pakistan?

Please share your opinion: do we need this vaccination?

*Probes:*

- *Do you know why the vaccine is given? Have you heard anything about the benefits of the vaccine?*
- *Have you heard anything that worries you about the vaccine? (e.g., safety, side-effects?)*
- *Have you heard anything that makes you feel positive/good about the HPV vaccine?*

Social Processes

SOCIAL PROCESSES & NORMS

- Perception about the vaccine

Q 1. Being representative of religious ministry, how do people working with you generally feel about new vaccines?

And how do people working with you specifically feel about HPV vaccines?

- Decision Making

1. Let's talk a little bit about the decision to get/not to get the human papillomavirus (or HPV) vaccine.

*Probes : In your opinion, who has the most important influence in decision making process*

- *Prompts*
- *Parents*
- *Who has more influence (among parents)*
  - - *Father*
    - *Mother*
    - *Both*
- *Family (mother-in-law/grandmother)*
- *friends, community members*
- *religious or community leaders*

Do you think girls should be informed about HPV? *Do you think that girls should be involved at all in the decision?*

If yes,

How should girls be informed? Who should provide this information, and how?

- Trust

1. If the HPV vaccine is recommended by healthcare workers, would people trust their advice?

*Probes:*

- *What about family and friends: If the HPV vaccine is recommended by healthcare workers, would people trust their advice?*

*Religious perspective*

*Probe:*

*What about religious or community leaders? If the HPV vaccine is recommended by religious or community leaders, would people trust their advice?*

*What is your perspective about HPV Vaccine being representative of religious affairs?*

Personal Thinking & feeling

Q. 1: HPV is one of the most common viral infections of the private parts. And certain types of HPV infections can cause cervical cancer and other kinds of diseases. The HPV vaccine is therefore given to help prevent HPV infection and the diseases it causes. How worried are you about your daughter getting cervical cancer?

*Probes:*

- *Why do you feel that way?*
- *How worried are you about your daughter getting cervical cancer? Do you know that cervical cancer is among the top 5 causes of death in females in Pakistan?*
- *How likely do you think that you will vaccinate your daughter?*

Q 2: How do you feel about your daughter getting the HPV vaccine?

*Probes:*

- *Alignment with spiritual or religious beliefs? Other beliefs?*
- *Thoughts about the safety of the vaccine? What about side effects?*
- *Thoughts on whether it will work.*

Q 3. Are there cultural or religious values that may affect your decision to accept or refuse the HPV vaccine?

What do you think that parents need to know in order to feel comfortable having your daughter vaccinated with the HPV vaccine?

Q 4: What is your opinion about the HPV Vaccine as a stakeholder of a religious ministry?

Motivation

Q 1. What do you think about vaccinating children (especially girls) against HPV?

Q 2. Would you allow your daughter to receive the HPV vaccine? Why or why not?

Q 3. What information would you need to feel more confident about giving your daughter the HPV vaccine?

Q 4. What questions do you want to be answered before deciding to vaccinate your child

against HPV?

Q 5. Who do you trust most when it comes to health information (e.g., doctors, religious leaders, teachers)?

Q 6. Is there anything g else you’d like to say?

**Qualitative Interview Guide (Parents)**

Aim: To understand multifaceted sociocultural and contextual factors influencing the uptake of HPV vaccine to support the launch of HPV Vaccination in Pakistan Objectives 1. To assess the current level of knowledge, belief, and factors associated with the acceptability of HPV vaccination among potential vaccine recipients and their parents/caregivers. 2. To explore stakeholders’ perspectives on the launch of HPV vaccination, considering the dynamics of the local population in the resource-constrained country, Pakistan. 3. To identify the social and behavioral factors that influence HPV vaccination acceptance and hesitancy within a local community (Punjab). BeSD domain: General Introduction: Hello, I am We are interviewing people to help improve vaccination services in Pakistan. The interview is expected to take __ minutes. Your participation is completely voluntary and anonymous. The answers you give will be completely confidential. If you do not want to answer a question or wish to stop the interview, just let me know. Would you be willing to take part in an interview with me? [If audio recording the interview] Would you be happy for me to record our conversation? Q1. Tell me a little about yourself and your family. Probe: (Who lives in your household with you/ How many children do you have? How old is/are your daughters? Are your daughters up to date with their vaccines?) Q2. Have you heard about the Human Papillomavirus (HPV) vaccine before? If yes: Where did you first hear about the HPV vaccine (e.g., media, school, health workers)? What do you know about it? Q3. Do you know what the HPV vaccine is and what it protects against? Motivation Q4. Thinking back, when you took your daughter to be vaccinated, tell me why you decided you would go ahead with it. Probe: (Did anyone suggest it? ; Who decided that you should take your daughter to have their vaccines? ; Who usually takes your daughter(s) to have their vaccines?) Q5. What do you think about vaccinating children (especially girls) against HPV? Q6. Would you allow your daughter to receive the HPV vaccine? Why or why not? Social Processes SOCIAL PROCESSES & NORMS(social stigma (embarassment talking about STI), geneder inequality, spouse communication, ineffctive communication with fathe, ower dynamics (strong father/mother in law influence over maternal authority in decision making, misconception, myths ) - Q7. Do you talk about vaccination with anyone else? (Probe: Who do you talk to? ;What do they say?; Do other parents you know vaccinate their daughters?) Q8. In your community, how do people generally feel about new vaccines? Q9. Do you think your decision to vaccinate your daughter would be influenced by others (e.g., family, religious leaders, community members)? Q10. Are there cultural or religious values that may affect your decision to accept or refuse the HPV vaccine?

1. Let's talk a little bit about your decision to get/not to get the human papillomavirus (or HPV) vaccine. How did you go about making this decision? Probes - Was there anyone else involved in the decision? Did you discuss your decision with anyone? (prompt spouse, family(mother in law), friends, community members?). - Were your child(ren) involved at all in the decision? Did you talk to her about your decision? Did she agree/disagree with your decision? Why/why not? - Was your spouse/grandmother 2. If the HPV vaccine is recommended by healthcare workers, would you trust their advice? Probes: - What about family and friends: If the HPV vaccine is recommended by family and friends, would you trust their advice? What about religious or community leaders: If the HPV vaccine is recommended by religious or community leaders, would you trust their advice? Practical Factors Q11. Thinking back to the first time you took your daughter to have their vaccines, tell me how you knew it was time to do so. Probe: (What kind of reminders do you use?) Q12. Thinking back to the time when you were getting your daughter vaccinated, how would you describe your experience in the vaccination/health centers in terms of accessibility, affordability (commute expenses), quality of services, attitude of healthcare staff, and ease of access? Q13. What challenges do you face in getting your child vaccinated in general? Probes…1. difficulty with coordination, language barrier, parda , social issues Q14. Do you think the services being provided at vaccination/health centers require improvement? If yes, what do you suggest how can the services be improved? Thinking and Feeling cultural and religious beliefs (e.g., perceived risk of premarital sex or promiscuity, concerns about pork gelatine), uncertainty around HPV vaccines and infection (often linked to perceived needs for more information), parents/caregivers thoughts that vaccination was unnecessary because their daughters are too young or because it was only relevant within the context of marriage, and limited knowledge about HPV/HPV vaccine parental responsibility for getting their eligible child vaccinated, parents’ more realistic understanding of children’s sexual activity, self-efficacy and confidence in one’s ability to engage in preventive action, confidence in vaccine benefits trust in healthcare Please add these questions 3. Pakistan is going to introduce HPV Vaccination for girls between 9 to 16 years of age Can you tell me what you've heard about the HPV vaccine? Probes: — Do you know why the vaccine is given? Have you heard anything about the benefits of the vaccine? — Have you heard anything that worries you about the vaccine? (e.g. safety, side effects?) — Have you heard anything that makes you feel positive/good about the HPV vaccine? 4. HPV is one of the most common viral infections of the private parts. And certain types of HPV infections can cause cervical cancer and other kinds of diseases in both men and women. The HPV vaccine is therefore given to help prevent HPV infection and the diseases it causes. How worried are you about your child getting cervical cancer? Probes: — Why do you feel that way? — How likely do you think it is that they would get the HPV infection? — How worried are you about your child getting cervical cancer? Do you know that cervical cancer is among top 5 causes of death in females in Pakistan? 5. How do you feel about your child(ren) getting the HPV vaccine? Probes: — Alignment with spiritual or religious beliefs? Other beliefs? — Thoughts about the safety of the vaccine? What about side effects? — Thoughts on whether it will work. Please merge these with the upper ones, Q15. What are your thoughts about vaccination? Probe: (Do you think it’s a good thing? Why? Do you think it’s important? Why? Is there anything you feel isn’t good about vaccination? Can you tell me more about it?) Q16. How do you feel when your child is vaccinated? Probe: (Do you think it’s good for your child? Why? Is there anything that worries you? Why does it worry you?) Q17. Do you believe the HPV vaccine is necessary for your daughter? Why or why not? Q18. Are there any concerns you have about the HPV vaccine (e.g., side effects, age, religious concerns)? If the HPV vaccine is provided, Recommendations: Q19. What information would you need to feel more confident about giving your daughter the HPV vaccine? Q20. Who do you trust most when it comes to health information (e.g., doctors, religious leaders, teachers)? Q21. Is there anything g else you’d like to say?

SOURCES OF INFORMATION AND TRUST 1. Have you received any information about the HPV vaccine from your healthcare provider? Probes: - - If yes: Did you trust this information? What makes you feel that way? If no: Would you trust information given to you about the HPV vaccine by your health care provider? What makes you feel that way? 2. Have you received any information about the HPV vaccine from your child(ren) 's school/teachers? Probes: - - If yes: Did you trust this information? What makes you feel that way? If no: Would you trust information given to you about the HPV vaccine by your child(ren) 's teacher/school? What makes you feel that way? 3. Have you received any information about the HPV vaccine from any other sources (e.g., the internet, friends, family members, media, or other sources)? Probes: - - If yes: Did you trust the information about the HPV vaccine given to you by [the source participant mentions]? What makes you feel that way? If no: What sources of information about the HPV vaccine would you trust? 4. Is there anything else you would like to add or comment on? 10. Any questions at this stage?
