# Supplementary material for: From global to local: Developing a context-specific BeSD-HPV tool through cultural and linguistic adaptation in Pakistan
Source: PLoS One. 2026 Jun 15;21(6):e0350162. doi: 10.1371/journal.pone.0350162 (PMC13268181; doi:10.1371/journal.pone.0350162)
Supplement: S2 File — (DOCX) [file pone.0350162.s002.docx]

**In-depth Interview with a grandmother (ABC).**

**Interviewer: Dr. XYZ**

**Respondent: A grandmother**

**Date: XX-YY-2025 Duration: 14.5 minutes**

**Interviewer:** Assalamoalikum, my name is Dr. ABC. We are looking for people's opinions on a project. People get their children vaccinated or not. What do they think about vaccines? Can I record your voice?

**Respondent:** Yes, you can.

**Interviewer:** Can you tell us about yourself? How many children do you have? How are their families?

**Respondent:** In the name of Allah, the Most Gracious, the Most Merciful. Peace be upon you. I have six children. I have three daughters and three sons. Vaccinators used to come to my village house on a motorcycle. Initially they used to come on a cycle and then on a motorcycle. They used to write the names and vaccinate my children.

Once my child got a lump after vaccination, so my father-in-law said we are not going to get vaccinated. My father-in-law is also educated, but the vaccinator counselled him very nicely and He told him that it is good for my children and my family so he agreed to get vaccinated. I have vaccinated all six of my children. Six months earlier they came to my house regularly and got the children vaccinated. Alhamdulillah, now we have vaccinated all my grandchildren and got their cards made too.

**Interviewer:** So, you have vaccinated your grandchildren too?

**Respondent:** Yes, I have. I don't want my grandchildren to get sick so got them the vaccine or else they might have suffered kaali khansi or khasra as they told us. So we get our children vaccinated and give them polio drops as well.

**Interviewer:** What do you think about what is the reason for vaccination?

**Respondent:** Vaccines are important so that there's no fever or kaali khansi, or khasra or any deadly illness or disability. If god forbid something happens they inquire about the child’s vaccination and tell us that because you don’t get vaccinated so the child is having this problem. Alhamdulillah all the children are fine.

**Interviewer:** You were the one who used to make decisions about your children. You live in a joint family Who decides about the health of grandchildren at home, whether they should get vaccinated or not?

**Respondent:** I am an illiterate, so my daughter in laws, their mothers are a little educated, but by the grace of God, they are getting vaccinated.

**Interviewer:** So, they get vaccinated on their own?

**Respondent:** Yes, they get vaccinated on their own. They know that they have to go to the hospital after 2-3 months, they keep a record if days go past the date. So, they are getting vaccinated on their own. Sometime earlier the youngest grandson , so his father said not to vaccinate him.

**Interviewer:** Why?

**Respondent:** He said previous vaccination had resulted in a fever and diarrhea so he had to be taken to the doctor, he had to spend a lot of money so didn’t wanted to take him for vaccination. So, I told him that the illness had come by the will of God, so there is no issue in getting vaccinated. I counselled them that he might be teething, so he has diarrhea. He had a fever, so I told him not to worry and get the child vaccinated by the grace of God.

**Interviewer:** Okay, so what is the opinion of people in your surrounding, do they also get vaccinated like this?

**Respondent:** Some people are getting it done, some are from Afghanistan, Pathan, they don't get it done, they hide the kids. They don't even get them take polio drops, and they don't even get them vaccinated. They say, our husbands don't let us get it done. Once the team came, then the second time, the third time. They said, no, won't. Then they brought their senior who told Pathan not to do this and to get the kids vaccinated.

**Interviewer:** Don't they tell why they don't get them vaccinated?

**Respondent:** I don't know, they're saying that they don't get it done, they say their children would get sick. That's what Pathan say.

**Interviewer:** Okay, tell me, when the COVID vaccine came, did you get vaccinated?

**Respondent:** Yes, I did.

**Interviewer:** Did the kids get it done?

**Respondent:** Yes, the kids got it done.

**Interviewer:** And their younger kids?

**Respondent:** They didn't get it done. The kids, that were younger than 12-13 years, they didn't offer vaccination for them so They didn't get it done. These kids were younger. We all got it done, we all got it done, and we got the card from Nadra.

**Interviewer:** Okay, so you got it done on your own accord, that this will prevent us from getting the disease.

**Respondent:** Yes, yes, absolutely.

**Interviewer:** Okay, so you don't think there's anything hidden that we'll get hurt or something like that?

**Respondent:** I was being said that if we would get the vaccine, we'll die in two years. They said, get the vaccine in two doses and then, you will die. I said, if time of the death had come we’ll die. I went to the hospital, I saw many influential people there like the brigadiers who had come to get vaccinated with their bodyguards with them I was very satisfied to see that they are educated people and they are getting vaccinated and We are poor people, we are uneducated. I was even more satisfied to see that and got vaccinated.

**Interviewer:** Do you appreciate that the treatment is free of cost?

**Respondent:** Yes, it is free of cost.

**Interviewer:** Do you think it is a good thing?

**Respondent:** Yes, it is a good thing.

**Interviewer:** How is the vaccination staff and their attitude?

**Respondent:** It is good, but the people in the hospital are taking more care of us. Here They come to the clinic of a doctor near our house, every Friday. They are also good if we go within their timings.

**Interviewer:** If you ask them about the treatment, do they give you a satisfactory answer?

**Respondent:** Yes, they give you a satisfactory answer. They inform about all the precautionary measures like ice application or a certain bandage to avoid formation of swelling at the injection site.

**Interviewer:** Have you ever heard of cervical cancer?

**Respondent:** What?

**Interviewer:** Have you heard of cancer of uterus?

**Respondent:** Yes.

**Interviewer:** Have you ever seen a patient with uterine cancer?

**Respondent:** Yes, it was in our village.

**Interviewer:** What was the problem?

**Respondent:** I think she had a phora (lump) inside from which foul discharge started coming. They kept taking medicine but then it got worse and then she died.

**Interviewer:** How old was she?

**Respondent:** She was 50-60 years old.

**Interviewer:** This cancer is very common in Pakistan and it is also common in women here. A vaccine has been invented against it. And it eliminates the virus due to which this cancer occurs. And it will now be given to a 9 to 16-year-old girl in Pakistan in the form of an injection. So we are taking advice about it. If you are offered an injection for a 9 to 16-year-old girl who is not yet married. What do you think about getting it for girls?

**Respondent:** I think it should be given. Because this disease is spreading a lot. You know about the aunty in our neighborhood suffering from uterine cancer. This disease has spread. So this should be done for your life.

**Interviewer:** So if you find out that this vaccine is being given free of cost. Will you get your daughters vaccinated?

**Respondent:** Let's see. God willing. If their parents are told too.

**Interviewer:** So it will be up to their parents.

**Respondent:** Yes. Then maybe the girls will also be satisfied. God willing. I say that if it is free and there are no problems in it then clearly if the doctor is giving such advice then it must be right. Why not? It should be done for your life.

**Interviewer:** So if you want to know more about this vaccine. Then to whom will you go to get information?

**Respondent:** I would go to a family doctor or an intelligent doctor. Who knows about this thing. Then the person should ask him first, See what is this vaccine? They are telling us about it, Should I get it or not? What is this? This should be done.

**Interviewer:** So if this is given to the girls in schools. Then?

**Respondent:** Then the school madam herself will satisfy the children or their mother will be satisfied.

**Interviewer:** Do you think the mothers should be called?

**Respondent:** Yes, they should be called. Maybe they would be called and told that this is good for your children, you should do it, this will be good for your health, and there should be no problem for the coming children.

**Interviewer:** Do you think parents pay attention to what the teachers say?

**Respondent:** Yes, they do. For example, the eye doctors come, sometimes other doctors also come.

**Interviewer:** Have they ever been vaccinated for measles in schools?

**Respondent:** Yes, they have been vaccinated.

**Interviewer:** So, did you give permission?

**Respondent:** Yes, I did.

**Interviewer:** Did the children get vaccinated?

**Respondent:** Yes, they got vaccinated.

**Interviewer:** So, the children were fine?

**Respondent:** Yes, the children were fine.

**Interviewer:** So, do you think that this thing will be accepted in your home and the girls will be vaccinated?

**Respondent:** Yes, Inshallah.

**Interviewer:** In your view, if parents and teachers are open to communication, then teachers can certainly reach out to some parents. Also, if we need to inform parents that currently around 11 million girls aged 9 to 16 in Punjab and Sindh are eligible for these vaccines, what do you think would be the most effective way to ensure this message reaches every household, every mother, and the general public?

**Respondent:** In every home, it is the same that first of all, they should message through mobile, tell everyone. And secondly, the teacher should tell, like someone who knows, he should tell. Now the internet is doing all the work. First tell this, and then Inshallah, the school will also do it. So, if the school does it, then the teachers and the children will also be satisfied. They will explain to them in simple words that this is not the problem, this is not the problem. So, Inshallah, it will happen in the future.

**Interviewer:** So, in your opinion, people understand if they are told.

**Respondent:** Yes, yes.

**Interviewer:** Okay, the people who are not, in your opinion, the people who will still not do it, that okay, my daughter is 9 years old, I don't know what vaccine to take. So, why do you think those people are doing this?

**Respondent:** I would just say it is jahliyat (ignorance). They don't understand that when the government and the doctors too are explaining they are not our enemies. They are doing it for their benefit. So, Allah knows how Allah is giving benefit to people And from where Allah is helping people. Inshallah, Inshallah, I say that this work should be done and all these problems should be solved. Disease, like this is a disease of the liver, it is a disease of sugar, and if such a person does it, then Inshallah, it will happen soon. Otherwise, the disease will not spread. Now, if you drink clean water, do clean work, then there will be no disease.

**Interviewer:** Do you have any other questions about this vaccine?

**Respondent:** I just say that spread it as much as possible. And the girls should also understand that this is for our benefit. We have to give birth to children, we have to get married, we have to do this work for this disease.

**Interviewer:** And in your opinion, the children who will be injected, should they also have education and awareness, should they also sit and explain it well, or will they listen to their parents?

**Respondent:** No, no, they should also be told that this is for your benefit. That my daughter, do this work. And this is also a protection vaccine for your life. It wouldn’t harm you, Otherwise, death and life are in the hands of Allah. Nothing can happen to a man. People will definitely object we don't know why they are giving vaccines, this and that. Now, you can't say anything about how people would think. But if the teachers and the doctors, explain it to the parents, then it will happen.

**Interviewer:** Thank you very much, …..Aunty.

**In-depth Interview with Dr. ABC (Gynecologist)**

**Interviewer: XYZ**

**Respondent: Dr. ABC**

**Date: XX-YY-2025, Duration: 27 minutes**

**Interviewer:** Assalam-o-Alaikum, Dr. ABC, my name is Dr. XYZ. I would like to take your opinion on HPV vaccine. As you know, it is the only vaccine that has been developed against cancer and is being rolled out in Pakistan.

Before that, it is being assessed that how people will be able to take it and what barriers will be there in taking it. So, since you are in contact with such patients who are directly affected by this disease, can you tell us how many patients come to you and at what stage do they come?

**Respondent:** Ok, if we see our population, particularly speaking about the Pakistani population, then I also have 17 years of clinical experience from your fellowship, which I have taken from independent patients. But, I have not encountered much of CA cervix, even I have not seen much of pre-cancerous lesions. Ok. So, if you say that our general population, like Diabetes is 50%, Obesity is 50%, PCOS is a rising trend, if we say that this is an epidemic, then there is nothing like that.

Ok, so general population, but obviously prevention is better than cure. If we screen the whole population and then see how many people will fall in that category and then do vaccination, then it will be a better option. But, if the government is supplying and there can be lifetime immunity, then why not, it should be done.

**Interviewer:** Ok. If you see that the patient's ratio is not that much, first of all, you tell me that the patients who come to you for CA cervix, are they screened for HPV?

**Respondent:** No, never actually.

**Interviewer:** So, is there any HPV screening here?

**Respondent:** There is no such thing in HPV screening in the government sector, but if you see the inflammatory changes or pre-cancerous changes on Pap smear, then you suspect that it can be due to Human papilloma virus, there are number of reasons of CA cervix, so HPV is the most common. In HPV, further genotype, if you see the type specific, then that is not happening. If you talk in private sector, then in private sector also, in every city, everywhere, it is not available.

**Interviewer:** Ok, HPV screening is not available?

**Respondent:** No, type specific genotyping is also not there. Otherwise, HPV is also not available as such universally. Yes, it is not that common test that you can do easily. It costs the patient about 6000 to 8000. Correct. If you advise HPV. And the benefit of that is obviously, only the diagnosis will be done. Yes, only screening and to know the causative agent. Otherwise, if you have lesion, then it will be known, treatment will not be effective. Treatment is the same for everybody.

**Interviewer:** Ok, so what do you think, if this vaccine is launched, then how patients will take it? When you are saying that there are not many patients in our society, so if people do not have awareness, then how people will react to this vaccine?

**Respondent:** People's reaction to take the vaccine will be the same as the COVID vaccine. Ok. For example, it took a long time in the acceptance of COVID, and some people have not got the COVID vaccination yet. The same will happen, most probably, about the HPV vaccine. Because if you talk about young adolescent girls, school going girls, if you say that they should get this vaccine, and their parents will be told that in the future, they will not get CA cervix, and particularly, gynecological issue and all this, then probably, their acceptance issue will come. According to our society.

**Interviewer:** And what would they predict, what can happen with this vaccine, that there will be a barrier?

**Respondent:** Our social myths are there, that many people get attached to it, that they have started introducing something, and our girls will get infertility, and these issues are obvious.

**Interviewer:** I would like to hear this, what else the patients tell you that it is not good.

**Respondent:** Vaccination is not known to the public yet, there is no such thing about it, but these are proposed, these are predicted myths, which are attached to any vaccine in our society. So, it can happen, but till now, people are not aware of this vaccine. If you talk about highly educated or better people, So, this acceptance issue will definitely come in our girls, for that, you will get a little barrier.

**Interviewer:** Okay, and if this barrier is to be overcome, then which would be the best modality?

**Respondent:** Do school going girls counselling, do their health awareness, start health education programs there, then only you will get this. Okay, so you will have to give it to the teachers at the school level. Because the minimum age for the vaccination would be 9 to 16 years. Yes, so that will be the school age, so you will have to go there and tell them.

**Interviewer:** And then parents, because obviously, if so many children talk for 9 to 10 years, then those girls obviously, if they understand something, then maybe they will be able to communicate a little to the parents.

**Respondent:** Yes, they will be taking the message home, obviously, when they will talk in their school again and again, then maybe they will talk in PTMs, parents will meet the teachers, now when this discussion will start, then obviously, it will be open to discussion, then these discussions will go ahead, then it will happen. So, it's a long process.

**Interviewer:** And to target the parents?

**Respondent:** You can target the parents in PTMs.

**Interviewer:** Okay, do you think that before the vaccine rollout, there should be some awareness?

**Respondent:** Yes, there should be awareness. Without awareness, it is not possible. Why do your planning programs fail? Because you are not aware.

**Interviewer:** And minimum, what do you think, how much time do you think people take to accept this vaccine? How much time do you think, on the basis of your experience, how much time does it take for people to start accepting it? And this information trickles down.

**Respondent:** It takes years, it takes years.

**Interviewer:** And do you see any difference between urban and rural?

**Respondent:** In rural, there is no acceptance at all. I don't think in rural areas. In our social circles, if you are talking about city, then you may not find so many barriers here. But if you talk about the whole of province, then depending upon the educational status, you will find barriers.

**Interviewer:** Okay cervical cancer, when you diagnose, are people told the name of the disease? Or is it just said that, this is the cancer of womb or uterus. How do you tell people?

**Respondent:** This is the carcinoma of the old age. Usually, the patients are dependent. And when they come, they are already very late. Because screening is never complete. And this disease takes a long process, from the screening to the disease. So, if we talk about that it takes time for them to open up, they don't open up because they don't have symptoms.

**Interviewer:** No, when they are diagnosed, they reach you, they are labeled as those patients. So, how are they explained and counseled? I mean, I want to know, what do the families think of that disease?

**Respondent:** They are definitely told that this is CA, this is carcinoma, this is root cancer, and this is on mouth of uterus, but it is not necessary to tell them what are the modes of transmission of this? No, this name is also not taken as cervical.

**Interviewer:** In common language, as you said, uterus mouth has cancerous lesion, so, like uterus, people still hear this word, but when we talk about cervix, that is not the norm.

**Respondent:** See, if not used as a typical word of cervix, but, the patient, to tell that what is its site, so, just telling it is present at the mouth of uterus is enough

**Interviewer:** When I ask people about this cancer, they get to know that this is something that belongs to women or something like that But this particular word, as saying cervical CA is, so then I get to know that in the common language, how people get to know about it.

**Respondent:** So, the people who have a little education status, they still do their research, a lot of knowledge through Google, and they see where this cancer is from, what is being talked about. So, in that way, they also get a sense of education.

**Interviewer:** Okay, in your opinion, the whole vaccination system of Pakistan, what are the supporting factors that can help in the launch of this vaccine? Are there any supporting factors?

**Respondent:** So, your paramedics, the influential people in your local areas, if you bring them together, then there will be a little feasibility in your awareness program.

**Interviewer:** That means people trust them, so this system is one, that if a person, an influential person is trusted.

**Respondent:** Absolutely, and specially our religious influencers, so we will also bring religious people together, then only this will work. Because this will become a stigma, that cervical cancer due to sexually transmitted infection, so it should not become stigmatized. You tell so much that it becomes stigmatized, that its transmission is going to happen.

**Interviewer:** So, in your opinion, if people have to be aware about this vaccine, to what extent information should be provided, so that would be enough?

**Respondent:** To the extent that this is a lifelong disability, this will be carcinoma, it will be fatal, it can lead to death, so prevention is better than cure. Okay. So, this is enough for our general population. If someone needs more information, you should have brochures, you should have the literature, so give them printed literature, tell them sites, give them links, that you can search from here. But generally talking about this carcinoma and availability of vaccine, so you just tell this much, that there is no cure for many tumors, but this tumor is such that it takes a long time to form, so if you apply vaccine, it will never form. So, this is one generally.

**Interviewer:** I also have a daughter and you are also mother of a daughter, would you go for it? We being health professionals.

**Respondent:** I have never thought of it, but if I talk of myself, I have never gone through the screening even, because there is no national program of the screening. So, what to talk of the vaccination then?

**Interviewer:** But the thing is that we people can afford this vaccine, even if it is not like given free of cost, so in that sense, are we motivated enough to like use it for our own daughters?

**Respondent:** I think not. We ourselves have a lot of reservations.

**Interviewer:** And what might be the reason? Why is Pakistani society that reluctant to call all these, means how is this thing bridged?

**Respondent:** Vaccinations, there are thousands of vaccinations available, if you think that I have got Meningococcal infection, so let me apply for Meningococcal, now Typhoid has come, so let me go for Typhoid, so in this way, you can apply thousands of vaccines. Your own antibodies will never develop, so all of you will go on Passive Immunity. So, this is also like that, means no one has a family history, no one has good religious beliefs, people do not have any infections, if you feel that you are protected enough, then why to go for that vaccination?

**Interviewer:** So, you think that it is not like that necessary, or like I said, we do our research and we are going to see what have been their positive effects, then we may opt for it. So, do you think that we should still think that this vaccine, as the literature says that this is a very leading cause of cancer in Pakistan, so we should go for it?

**Respondent:** See, if we see in clinical settings, then how many patients we have seen of CA cervix, if CA was so common, then why we do not have 50% patients of ca cervix in our ward, if you see CA cervix against ovarian CA, then in 6 months, only one CA cervix is reported, we are talking about Teaching Hospital, obviously, CA cervix will not be missed, it will come from somewhere or the other in Teaching Hospital, so no one takes the tumor like this and does not keep it with them, so it is not possible that you have missed CA cervix. So, my opinion is that we are not seeing so much CA service, that we should put so much cost for it.

And bring in such things with which have religious myths, religious stigmas and many other things attached with the patient, so I do not think that our general population will accept it so soon.

**Interviewer:** One thing, if someone is taken on the board among Health Professionals and they are asked to do awareness sessions, do you think people who are very staunch with regarding to stigmas and myths and misconceptions, they would like react negatively towards the Health Professionals, that they are saying such things or how they react against the polio workers.

**Respondent:** Some tribal areas, very low social classes, very low educational status people will do it, but generally I have seen that people have a lot of conservative approach and diplomatic approach, so they will not say anything to you, they will not come to you second time.

**Interviewer:** And do you think that our population will trust the Health Professionals, because many times people used to say that we will ask our Gynecologists, so do you think they trust?

**Respondent:** Yes, they do, their families do it for their children, but if you ask Gynecologists that how many of our patients we have told to start their daughters the vaccine when they are 9 years old, then probably not. We have not told, because we are not on the page yet.

**Interviewer:** And all the Health Professionals, Gynecologists you have worked with, do you think they are going to participate in this campaign, if this awareness campaign starts, considering the fact that they have not seen so much patient load, do you think they would be convinced?

**Respondent:** Yes, there are many concepts, people will go with it, it depends on what are their vested interests, they will go according to that.

**Interviewer:** Anything else you want to add?

**Respondent:** I think that before vaccination we should have started the national screening program, so that once we have the data available that how much population is at risk of having cervical carcinoma, then you should go for the cervical vaccination. You should go for the very cheap test which is available, readily available, anybody can do this.

And screening is obviously 3 yearly, has to be repeated, so that you have to do even after your vaccination.

**Interviewer:** In DHQs, we have seen that there is Pap smear, so that is happening,

**Respondent:** but not for everybody. As a screening, screening means that you are registered for this purpose of screening, and in other developed countries, you are registered, since you are registered, then every 3 years you get an appointment, you get a call that this is your time for screening. So, you get a reminder, you don't have to leave it on auto, screening means that if a program is going on, then you have to follow it regularly. So, you have the card, you have certain restrictions that your ID card will not be renewed until your screening report is done. So, there can be many steps in this.

**Interviewer:** So, you think that screening should be done first and then follow up. Anything else you want to add?

**Respondent:** No, I think it is a good program, but there should be the steps to follow.

**Interviewer:** Thank you so much.

**In-depth Interview with Dr ABC (……in Gyne/Obs, ………….. Hospital).**

**Interviewer: Dr. XYZ**

**Respondent: Dr. ABC**

**Date: XX-YY-2025, Duration: 18.5 minutes**

**Interviewer:** Assalam-o-Alaikum, my name is Dr. XYZ and we are doing a project on vaccine uptake that how much is the receptibility of vaccines in our general public. And specifically I want to ask you about HPV vaccine. So, can I record your interview?

**Respondent:** Yes, of course.

**Interviewer:** Can you first introduce yourself?

**Respondent:** My name is Dr ABC. So, I am working in Gyne Obs, ………Hospital.

**Interviewer:** Okay, and how long have you been working here?

**Respondent:** I have been working here at DHQ for more than 10 years.

**Interviewer:** So, do you have any experience in cervical cancer? How many patients have you encountered?

**Respondent:** I can't give you numbers exactly, but I think nowadays one or two patients come every month.

**Interviewer:** And how long have you started to notice this increase?

**Respondent:** For the last 2-3 years.

**Interviewer:** And is there any particular trend or age group that is affected by this?

**Respondent:** No, there are very unusual presentations that are being presented, very young ages are being affected. In the last year, there were 2-3 cases that were in their 30s. Otherwise, most of these patients are of old age, in 60s and 50s, but the current trend is of early age.

**Interviewer:** And at what stage do they present?

**Respondent:** 3^rd^ or 4th.

**Interviewer:** And do they present before that?

**Respondent:** No, no.

**Interviewer:** Do they go to a doctor?

**Respondent:** Yes, they go with symptoms of Vaginal Discharge, multiple times with post-coital bleeding, to GPs and to small clinics, but no one makes a proper diagnosis like this, no one does an examination, no one does a test.

**Interviewer:** So, is there a Pap smear done here or what is the line of management?

**Respondent:** Foremost we get a Pap smear done. First, if there is a Vaginal Discharge, then a proper course of antibiotic is given, after that patients are given a follow-up, after that a test is done. So, mostly the problem is that if the patient is given treatment, she gets well, then she doesn't come for follow-up. So, loss to follow-up happens.

**Interviewer:** And Pap smear is done, and if there is atypia in it, then you go to Biopsy or any other test?

**Respondent:** Yes, we also do Human papilloma virus, we have started that too. So, it depends on that, what type of dyskaryosis appears mild, moderate, or severe, according to that with HPV then according to that follow-up is done.

**Interviewer:** Is HPV screening done on blood, how is that done?

**Respondent:** No, no, that is also done on sample of Papsmear, you take Liquid Based Cytology, and that is done in the same sample. Yes, Cervical Scrapping, from that HPV is also taken. It is called Liquid Based Cytology, both are done.

**Interviewer:** And how often do you get it done? I mean, do you get it done from every patient of atypia?

**Respondent:** Yes, we do. It is done from outside the hospital and it is a little expensive, so very less number of patients get it tested, as there is an issue of its affordability, that it costs around 15,000 rupees So, the community that comes to us, they don't have that much affordability. We also do Biopsies of the patients, if we find any Unhealthy Cervix, and if the patient is not affordable, then we also take Biopsies.

**Interviewer:** Okay, if we talk about HPV Vaccine, so how much do you recall, that you have counseled the patients

**Respondent:** We get patients, who have passed the age group of vaccination, they are Sexually Active, they are Married. Yes, if someone comes at a young age, if someone presents, then we definitely tell, we also tell the family, but if I tell you frankly, we don’t do effective Counseling.

**Interviewer:** Okay, the people whom you have counseled for HPV Vaccine, so do they get Vaccinated?

**Respondent:** I have advised one or two people in the last few days, and they said that they couldn’t find it in the market.

**Interviewer:** Okay, means they are not available in the market.

**Respondent:** Yes, they didn’t get it.

**Interviewer:** So they were convinced that they should get vaccinated.

**Respondent:** Yes, I had some colleagues, who had Daughters, 10 years old, 9 years old, I told them to get them vaccinated, so they were convinced but said, it is not available and so they couldn’t get vaccinated.

**Interviewer:** Okay, if this Vaccine is rolled out in Pakistan, for the general public, how do you see its receptibility, how will people accept it?

**Respondent:** I think, if we give awareness, and we, I mean, through social media, and through Camps, and if we give awareness of these things, then people will get it vaccinated. Right now, a lot of other vaccines are also being used, right? When COVID came, people first refused, but finally got vaccinated even if 60% of the people got vaccinated, so it is the same for us, that even if 100% people do not get vaccinated, but 60%, 50%, this is also a very big number for us.

**Interviewer:** So, in your opinion, regarding this age bracket, will there be any cultural or religious misconceptions and myths will arise?

**Respondent:** There will be, there will definitely be, because it’s a norm here that if any vaccine is introduced, people start making myths, that this will happen, fertility issues will happen, that will happen, but definitely now there is a lot of awareness among people, educated people, they understand these things.

**Interviewer:** So, what do you think about the general public that comes here, the general hospitals that come here, what do you think, if they start vaccinating the girls of this age group, what will they think?

**Respondent:** If it comes from the government, then they will definitely get vaccinated, and if it remains private, then the public coming here will never get vaccinated. If it will be available free of cost and the program is run by the government, if someone spreads awareness, if the government does something, then definitely it will happen.

**Interviewer:** Okay, and in your opinion, as HPV is sexually transmitted, so when we start giving awareness, so in your opinion, to what extent should the information be delivered to the patients, and how should it be done, that they should be convinced to get vaccinated?

**Respondent:** Well, it's basically counselling. Yes, it should be told, it should be told clearly that it is sexually transmitted, and for this, it is very important to get vaccinated, and you can be saved from a very big disease. It is such a good preventive measure, it should be told. And it should definitely be told that it is sexually transmitted.

**Interviewer:** Okay, now I will ask you about the girls. Now these are big girls, 9 to 10 years old. So in your opinion, should they be educated?

**Respondent:** No. I mean, it's okay to tell them, but you can't tell them so openly that it is sexually transmitted. But their mothers can be counseled. Its Just like any other vaccinations for them, they also get vaccinated for other diseases as well so It is a preventive measure, but you can't tell them so much in detail about the sexual activity, you can tell them about the periods and everything, but in my opinion, girls of 9 to 10 years old are immature, they cant be told so much, but obviously, their parents can be counseled.

**Interviewer:** Okay. Now, if we talk about the parents, so in our country, the males are the decision makers.

**Respondent:** No, I think both males and females, both parents can be counselled. And it should be told. Because males absorb things more, understand things, and their perception is better then the females. So if you do it to both parents, that is, father and mother, I think it will be more successful.

**Interviewer:** We were talking about parents, as mothers are in contact with gynecologists. If fathers are to be targeted to create awareness, then what would be the best forum or modality to create awareness?

**Respondent:** Social media should be used. Everyone watches social media.

**Interviewer:** If we run a campaign on social media, who should address the people?

**Respondent:** Gynecologists and oncologists. Oncologists will have more effect as they can use more fear. They can highlight the side effects. They will tell us how many cases are coming, how they are presenting, how they are coming in the last stage. That will have more effect.

**Interviewer:** Ok, if this rollout happens, as you said, the market is short. If the government takes an initiative, do you think there will be any issues with access?

**Respondent:** No.

**Interviewer:** Ok, so was the covid vaccine freely available to you?

**Respondent:** Yes. When the government takes a step, then everything is fine.

**Interviewer:** Ok, and people who come from far-flung areas. Will they be so motivated to get the vaccine that they will come or will we have to send them somewhere?

**Respondent:** We will have to do it at every level. We will have to do it at every district. Not just tertiary care hospitals. Just like all other vaccinations are being done at every level. We will have to do it at primary, tertiary, secondary, every level.

**Interviewer:** Do you want to give any other recommendation for its successful rollout?

**Respondent:** I would say that more than vaccination, if we emphasize on cervical screening, that the patient should follow the schedule of cervical screening, then we can save ourselves from a lot of things, which is Pap smear. There is no awareness of screening, then vaccination is a lot more advanced step.

**Interviewer:** So, do you try for its awareness?

**Respondent:** We do, why not? I am telling you that we give treatment for 7 days, we tell the patient to come after 7 days and get Pap smear, but the patient does not come. 2 out of 10 patients will come, 8 will not come.

**Interviewer:** And why do you think they do not come?

**Respondent:** Just issues of affordability, economic issues, awareness issues.

**Interviewer:** So, Pap smear can also be done at THQs. Its available there.

**Respondent:** They don’t do it, its carelessness on the part of doctors but our public is very carefree, they are not concerned, due to illiteracy.

**Interviewer:** Ok, sometimes it happens that you have written to someone, and he did not come at that time, you understand, then he came for some problem, then you asked him that when I had written, why did you not get it done? So, what do you say?

**Respondent:** “Children are small, it is very difficult to come to the doctor, where does the husband go, with whom should we come? There are a lot of issues.” These are the answers.

**Interviewer:** So, the point is that these are such procedures that cannot be hospital-based, it cannot be that this is a doorstep, how polio drops are given, so obviously they will have to come.

**Respondent:** They will have to come. There are the problems of coming.

**Interviewer:** And if we were giving free of cost here, then also these people do not come.

**Respondent:** For screening, we are free of cost, they just have to pay 2,000-3,000 for the cytology, but we do not take money to do it.

**Interviewer:** So, that may be the reason that they do not come.

**Respondent:** No, we have not told them yet, that they have to get it done from outside, that there is a test, they do not even come to get the examination done.

**Interviewer:** Right. Thank you so much.

**In-depth Interview with Dr ABC, …… Department of Pediatrics**

**Interviewer: Dr. XYZ**

**Respondent: Dr ABC**

**Date: XX-YY-2025, Duration: 18:37 minutes**

**Interviewer:** Assalam-o-Alaikum, my name is Dr. XYZ and I am very thankful that you have given your precious time. We are doing a project on vaccine uptake and specifically on HPV vaccine uptake. Since you have a lot of experience with children, your opinion will be very important for us. Can I record your interview? Yes, of course. First of all, please introduce yourself.

**Respondent:** My name is Dr. XYZ, I am an ……and ……..of Pediatric Department at ……. Medical University. How old is your experience? After my graduation in …., I did a house job in Pediatrics. I did not work anywhere else apart from Pediatrics. That makes it about 20 years.

**Interviewer:** Let's start with vaccination. What is your experience with child vaccination? Do you think people are 100% motivated to get vaccinated?

**Respondent:** I have worked in city. In big cities, deliveries are usually done in health care facilities, not at home. When children are discharged from the hospital, the vaccination card is provided to them. Initial birth vaccines are given to them so Parents think it is important to get vaccinated. Most of them follow the vaccination schedule.

**Interviewer:** Do you think there is a barrier that prevents people from getting vaccinated?

**Respondent:** I think the previous generation had a lot of misconceptions and confusions. In Pakistan, there is a family setup. Parents decide whether to vaccinate their children or not. It is not just the decision of the parents. There are other stakeholders such as grandparents, aunts, uncles. Someone said that a child in our area was vaccinated. After that, he stopped walking. After that, the child got very sick. Such cultural and educational circumstances have a negative impact.

**Interviewer:** Do you think that is why some people drop out?

**Respondent:** Yes, because of the influence of the older generation. Because of their misconceptions.

**Interviewer:** Most of the parents I have seen are more receptive. They get their children vaccinated. It is also possible that the vaccination card is required later.

**Respondent:** I am not sure about that.

**Interviewer:** How did you feel about COVID-19? Because it was a new vaccine. What was the receptibility of that vaccine?

**Respondent:** There was a lot of vaccine hesitancy. I know a lot of doctors. They said that it has a lot of side effects. Heart attack, sudden cardiac arrest, etc. It was related to the vaccine. There were so many misconceptions. Even educated people were hesitant to get COVID-19 vaccines. But I got it done. I also got my children vaccinated. There was a school campaign. You have to give consent. I gave my consent. There was no interaction with people because of the lockdown. I shared my vaccination story on social media. To make the vaccine acceptable. To promote it. To reduce people's fears. I shared my vaccination story. I am myself a doctor. I also got my children vaccinated. So don't be afraid.

**Interviewer:** Did people contact you about your awareness?

**Respondent:** Yes. People gave me feedback that seeing your story gave us a confidence to get vaccinated. When you think it is safe for your children. It will definitely be safe.

**Interviewer:** If you want to rate that response. Do you think it made a difference? You got overwhelming response? Do you think if awareness is created it makes a difference?

**Respondent:** It really makes a difference. If Intentions are good, your communication makes a difference. Yes, my intentions were good. I was wondering why people are hesitating. Studies have shown. That severe cases are less. Even If you are sick. You won't need ICU care.

**Interviewer:** What about doctors? Why were they hesitating?

**Respondent:** There were studies. It is not a bad thing. You should decide after seeing both. There were studies. With specific vaccines. With side effects.

**Interviewer:** When we talk about HPV vaccine. Pakistan is intending to roll out this vaccine. Because it is the only vaccine against HPV virus. The age bracket would be 9 to 14 years, unmarried girls so first of all you will share your thoughts that what do you think how will be the receptibility of this vaccine in our public?

**Respondent:** I think there will be a little problem in this compared to other vaccinations because in our country reproductive health is still considered taboo and if children are asked what is the purpose of this vaccine, they will feel shy or they are going to the hospital to get vaccinated so what will they tell their family members, their father, brother, what is the injection for so if we overcome this shyness then I think vaccination percentage will improve

**Interviewer:** And what do you think is the best way to do this?

**Respondent:** Talking more about reproductive health probably

**Interviewer:** And which forums like obviously?

**Respondent:** On televisions, social media, our people have more reach, mobile phones are also available you can use podcasts, youtube or other channels, print media, electronic media campaigns I think if you see a few years ago then it was also a taboo subject but now no one feels awkward if there is a TV and there is an ad for birth control or if there is a talk of Subz-Sitara because people have got used to listening to it, in the same way like sanitary pads used to be considered taboo but now every channel has it, earlier it was hidden in grocery stores and now if you enter the grocery store, it is kept in the first rack and women pick it up from there and keep it in the grocery basket so when you do this a lot then shyness or hesitation reduces for those things and I think this can be a social norm, if you look at it from an Islamic point of view then there is no shyness or hesitation about these things in Islam even in the Holy Quran, all kinds of pure and impure, haiz, nafas, health, all issues have been explained very well because we are not familiar with the Arabic language so when we read it we are not used to listening to it but the child who is speaking Arabic from the beginning, a 3-4 year old child, when he starts reading the Holy Quran he is in his own language so obviously he is aware of these things whereas in our society, even when we are telling children about Islamic issues these things are kept hidden or under the cover so in my opinion, we should also look at this aspect that it is just a social set up and it is a good thing to talk about it from an Islamic point of view

**Interviewer:** Do you think that social media would be enough to convince fathers or fathers separately, because our society is male dominated and a lot of females are brought here by males so fathers should be targeted in a different way

**Respondent:** You can do it individually, not in a group if you do it in a group then I don't think it will be beneficial like if you have a couple or a family, you can make them sit together and explain ok,

**Interviewer:** So if people in our society talk individually then which forum do they trust the most?

**Respondent:** Doctors, if they have come to get their adolescent daughter checked because our age group is flexible, we are in some hospitals for 12 years but the children's hospitals like the one in Lahore, there are 18 cut-off so 16-17 year old girls and boys come there so the age group of 11-16 years, where it is proposed that this vaccine is compulsory so if they visit a general physician for any illness so a general physician should counsel both the parents so we can do this, that medical specialties and pediatricians and the GPs, our majority community goes to the GPs so you can involve primary, secondary health care, dispensaries or private setups where there are GPs. You can involve them in this educational seminars conducted for them so they know the importance of this so they can recommend this to their patients and along with this, there should be a campaign which should be done by the proper health department so that the patients don't think that this has the benefit of a doctor that it has got a percentage because these are also trust issues.

**Interviewer:** Ok, so if this vaccine is available free of cost as it is known, then obviously as it is expensive otherwise so do you think, as you talked about the money do you think that they will think about this

**Respondent:** Yes, regarding this, our population has a misconception about polio drops that whatever work is being done here, iodized salt is being introduced so it is being done to forcefully reduce our birth rate to cause infertility to people similarly, polio vaccine is also there maybe the non-believers outside want our birth rate to be reduced however, if you think about it, this is our own problem to control the population, we should be worried about this why are those non-believers so worried about us that if our population is reduced then many of our problems will be solved and our country will develop in fact, if they don't recommend it to us then they should have thought about increasing the population so that our children stay in their homes so this is a big misconception so the first thing that will come is that this is a conspiracy of non-believers to make girls infertile that this vaccine is being given.

**Interviewer:** I think in public hospitals there is a lot of patient load and I think this is still going on so do you think that doctors would have time to counsel the patients and the girl would also be of this age so the first question is, do you think the girl needs counselling and secondly, do you think the health workers would have the time to counsel them to address all these things

**Respondent:** In my opinion, we have tertiary care we have nurses we have medical students we should make them a part of our workforce because for counselling there is no harm it is good that they get used to community service so if we engage them then this can be done and as far as the girl is concerned she should also be explained so that she is educated and knows that it is for her benefit but for decision making, I don't think that the girl can take her own decision whether to get an injection or not so that decision is up to the parents.

**Interviewer:** So here, vaccination obviously, you have EPI so does it ever happen that there is a shortage of vaccine or there is a problem of availability

**Respondent:** Yes, it happens, for example, MMR vaccine is not available Mumps vaccine is already short in Pakistan so it was MR yes, it is because of availability otherwise, there should be MMR especially in boys who have serious complications. MMR is for 5 years MMR is recommended for small children and it should be done but it is not available in Pakistan

**Interviewer:** So do you think that such problems can arise because of this vaccine because it is new and expensive did it happen in Covid vaccine?

**Respondent:** No, it did not happen

**Interviewer:** And as far as access is concerned is there been any problem mentioned by patients, for example for vaccination there is access and public centers people say that there is outreach and mobile services

**Respondent:** I think this is a good setup of EPI and the cold chain of government vaccines is also better as a pediatrician I prefer government vaccines because I have more trust on them and I think their source is trustworthy so I would choose for my own children I think there is no issue of outreach

**Interviewer:** Being a mother ourselves, if you have a mother and she is afraid of side effects so how do you counsel her?

**Respondent:** I will have to read the side effects because I don't know what happens with every vaccine obviously it is safe and it is recommended for all children if it is considered safe by WHO that means this vaccine is safe

**Interviewer:** So how much depth of information do you think should be told to a patient as it is sexually transmitted and it can cause warts so how much detail would be sufficient

**Respondent:** I think if they are told that it will be a reason for causing them cancer of uterus and womb later on in life, and the vaccine can save them later, then that will click more because it is very common i.e. dysfunction uterine bleeding is a very common symptom in 40 plus age group and most of the women go through surgical procedures and their surroundings have heard about it and they have gone through DNC and they have gone through so if we talk about STD then I think it can have a negative impact

**Interviewer:** And should side effects are mentioned a lot?

**Respondent:** Some patients may have allergic reactions but if you know the source that it is fresh and its cold chain is fine then it is very rare

**Interviewer:** And do fathers talk to you about their child illness

**Respondent:** We call fathers for counselling

**Interviewer:** And do they take initiative to ask

**Respondent:** Some people do, some people think that the mother does not know anything so I will talk to her so women and fathers both interact and some relatives are also more interested so that they can tell the whole village that we asked the doctor and they told us this

**Interviewer:** Thank you so much, any other recommendation for a successful rollout of this vaccine

**Respondent:** I think you have covered a lot and it will be a good step

**Interviewer:** And do you think that preliminary awareness is a must

**Respondent:** Yes, start it now and when it launches the response will be good

**Interviewer:** Thank you for your precious time.

**In-depth Interview with Dr. XYZ (Technical Officer, new vaccines) FDI.**

**Interviewer:** Dr. ABC

**Respondent:** Dr. XYZ

**Date:** XX-YY-2025, **Duration: 41 minutes**

**Interviewer:** Assalam-o-Alaikum Sir, I’m Dr. ABC. We’re here to get your perspective on the vaccination programs in Pakistan, with a particular focus on the HPV vaccine. We’re currently working on a project at the ABC centre called *Cultural Adaptation and Validation of the BeSD Framework*—which stands for Behavioral and Social Drivers of Vaccination. This framework is meant to support the introduction of the HPV vaccine in Pakistan.

We’re research associates working under Dr.abc, the principal investigator of this project. As part of our work, we’re conducting qualitative interviews to help tailor this framework to the local context. For this purpose, we’d like to hear your thoughts. With your permission, may we record the interview?

**Respondent:** Yes, please. Welcome in FDI, it's a good initiative, let's see how we can move forward.

**Interviewer:** Sir, first of all, please tell us something about yourself, about your designation.

**Respondent:** I am Dr. XYZ and here in FDI, I am working as a technical officer, New Vaccines, starting from its submission to application, most of the time, whichever vaccine we introduce, we do it through GAVI. The benefit of GAVI is that we get the vaccine in co-financing, plus if we have a big cohort, for example, 9 months to 15 years, 9 years to 15, 14 years, if we have to do a big chunk cohort, then we get a campaign in it, which is a big support and again to dent that specific disease, so in that regard, we submit with support from GAVI. So, starting from submitting the application till the execution and implementation of the said vaccine and introduction in R.I., there is a contribution from my side in that.

**Interviewer:** Sir, regarding surveillance, campaigns which are launched, in all of these, you people are the managing team over here?

**Respondent:** Yes, all the team is ours. I mean, if you take the specific name of surveillance, then for surveillance, my expertise is not as such, but we have a complete team.

**Interviewer:** Okay, sir. Sir, so we directly, I think, come to HPV vaccine. So, sir, at what level is it? How is the government on it? At what level are we now? Will we launch it? Please tell us something about it, sir.

**Respondent:** Yes, as I told you, we collaborate with GAVI and introduce any vaccine. So, we submitted the application to GAVI, it has been approved and this is a three-year phase all over Pakistan and in which we are in the first phase, first phase is 2025, in which we are here in …., ….., ……, …., which is 70% of the country, with the population, so that is approximately 70% of the country.

So, here we are launching in 2025. Our expected date till date is, we will go into campaign mode first, in which we will introduce approximately 11 million female girls of 9 years to 14 years. In this, we will also tell you that what we are introducing in HPV, we are addressing only female girls, that is, we will target them, there is no male involvement in this, that we will not vaccinate the male.

Approximately, in this, we will address 11 million, more than 11 million and we are thinking to did this campaign this year. Okay, so it will start in ….. The campaign will start, after that, it will come in routine immunization.

And you are saying that you are planning to achieve 11 million in 2025. So, has there been any planned checkout, where will you approach or your usual EPI centers in our hospitals or? Since you are talking about modality. Yes, how are you going to like it? Okay, in this, our routine strategy is that we do EPI in three ways.

When we are vaccinating children under 11 months, then our strategy is in three ways. Fixed site, outreach and mobile. So, we will follow the same strategy.

Vaccination will be available on the fixed site for this campaign. Outreach to reach the target population in the community to the near doorstep of their homes. But again, we will knock on every house, but there will be no vaccination at every house.

Fixed outreach means that a team will go and sit in one place. Because it is an injection. It is announced in the mosque.

Announced in the mosque and it is an injection like polio, it is not the drops. Injection safety is a must for injection. Cold chain is a must.

Injection safety is a must. So, we don't go to every house and get vaccinated. We sit at a point.

We have social mobilizers. They knock on the house and move the target population towards the center. And there is vaccination there.

This is your outreach. And some hard to reach areas, some areas which are busy areas. We use a mobile team there.

These are the three strategies. This will be our vaccination strategy. Then we have to develop another strategy in the sense that schools and out of school girls will be there.

We have developed a proper strategy for schools. And we are expecting that approximately 50% of the population will be from schools. And for 50%, it varies from province to province.

But approximately 5.5 million population will be from out of schools. So, for out of schools, this outreach strategy is our strategy. Sir, before launching this, is there any awareness session for health workers? Yes, there is.

That is a part of it. There will be awareness sessions, school sessions, media briefings. Then there will be all kinds of media.

Electronic, print, even social media. So, when will you launch this? We have said that we will do it in September. We will do a little bit now.

But we will do it two months ahead. Because our target is September. So, if we do it two months ahead, then we will have something in mind to try.

We will start it approximately two months ahead. Sir, HPV, because the nature of this vaccine is that the age group is being targeted. And especially females are being targeted.

So, what are you predicting? Just like you introduced a new vaccine for normal vaccines. So, the factors that are favorable and show hindrances from that side. Are you predicting the same? Or do you think that you will have to be more aware of this? Or how do you feel about this? If you are saying in the context of the campaign, We have done a lot of campaigns in the context of the campaign.

Addressing this age cohort. Like when we did the campaign for typhoid. We went up to 15 years.

When we did the campaign for MR. For measles, rubella. We went up to 15 years at that time. So, in that, as such, maybe 49-51 ratio or 40-60.

There is not much difference. It can vary from province to province. Maybe there is a little difference in …. and …..

The female ratio can be 40-60. But overall we addressed this. But the special difference that is coming here.

It is coming in the context of unisex. Because earlier we did it across the board. Irrespective of male or female.

So definitely we should have fear in it. But there is no fear. But there is a sort of.

That this thing can come. And for that, we have started mitigation. A proper communication strategy has been developed for it.

Against which the provinces are making their action plans. Sir, will tell you a little bit about it. Because, for example, typhoid.

Like our child. So in the injection form. First they circulate a brochure.

That parents have permission. Then like next day or the coming day. Then the vaccine is given to the children.

So obviously when that vaccine is given. Typhoid is heard by everyone. If you have a stomach disease or measles.

People are well versed with these diseases. When it comes to HPV. It's like new.

You will find out. Cervical cancer. Means from that.

If anyone searches on Google. So parents will also search. And the girls themselves are also in such age groups.

So in that regard. You have obviously taken some measures. According to the awareness.

Very clear messages. And that. Yes, God willing.

The messages will be very clear. I thought of this thing. That we have done this focus.

That we. The concept of sex. We will keep it a little suppressed.

And the element of cancer. We will be promoting it more. With sexual.

We will keep its relation minimal. It is also not possible. That you are not giving information.

Minimal information. Minimal it will be. But the more promotion it will be.

That your. Saving from cancer. We are doing more.

So you think parents would be. Definitely questions will come. And its.

We have also prepared its FAQs. We have. What do you say.

We have prepared FAQs of different levels. Own of doctors. Own of female staff.

Own for the community. We have prepared FAQs for everyone. We have a helpline.

We will train them. That if they get phones.

I don't know if you have experienced. During covid times. We will activate it.

All the concerns. We will try to address them there. But definitely.

We are entering a new age. Before training HR, we have also tried to address typhoid and such campaigns. We are well-versed in campaigns, it is not such a big issue.

Like before this, we have also been campaigning for MNT, for TT vaccine, tetanus toxide, and we have also done for TT, tetanus diphtheria. So that is purely for females. But for that, when we are doing TT vaccine in the program, we are only doing it for pregnant women.

But when we go into campaign mode, we are addressing all the child-bearing age, 15 to 49. So in that sense, we have something. But again, in this case, because this is a very sensitive age, we have encouraged women staff to come forward.

First of all, you talked about training. We have taken a step back from training. Even if you come back, we will try to have female staff to address this age.

We will also involve nurses, female medical technicians, we will coordinate with nursing schools. And according to the school health strategy, in the majority case, our maximum effort will be that our female staff vaccinate the female. This is for the staff.

And the second thing is what material will be given to them. I have told you that we have FAQs and training for them. All these things will be tried accordingly.

And is there any difference in maintaining the vaccine cold chain? No, no. It is 2 to 2.8. All our vaccines are at 2 to 2.8 degrees centigrade. And it will be in injectable form.

Injectable form. We got it different in COVID. Otherwise, our routine vaccines are all at 2 to 2.8. We talked about this in the context of the campaign.

Yes, in the context of routine, we are adding a new age cohort, a new age bracket. We had no experience in routine until now, except that of pregnant mothers. But for T.D., it used to be that a pregnant woman used to be a better age bracket.

This age bracket is very sensitive. So for this, we are definitely working on it. And let's hope that we will succeed.

Sir, there is also a religious concept attached to this. That with polio, people usually cause infertility. There are myths and misconceptions.

Yesterday, the women were their own daughters. So they were concerned that this is what happens in our minds. Maybe this is coming from outside.

So this birth control is the birth of our Muslims. They had some comments like this. Do you have a program to train religious people for these myths and misconceptions? Yes, definitely.

The training is different. We call it training sensitization or awareness creation. All of these are included in the program.

PMA, PPA, SOGP, cancer, etc. These are all religious affairs. We have a technical working group.

We have added a member there. We are trying to get him. We will get our favorite things from him well in time.

As you said, there are announcements from the mosques. The same thing can be announced from the mosque. If they have accepted it, then it happens.

We will definitely see this religious factor and address it. Sir, I am a doctor myself. But still, as a mother, your concerns are a little more.

For example, if I am a mother and you have to convince me for HPV vaccination, how are you going to convince me? You have a lot of experience. So I want to ask that if I meet my parents in the future, how can I convince them as a layman? You are absolutely right. I will not give an expert opinion on this.

Because we have a whole ACSM team. If you are aware of ACSM and polio, they have different categories. How to talk to hardcore refusals? And how to talk to a normal parent? I will repeat the same old thing here.

We will focus on the point in time. It is the only vaccine which is addressing the cancer. Our main focus will be on this.

Other than that, the educated parents have to Google. They have to find out. All things will come in front of them.

I don't think there will be such bad things in Google. You are right. For those who do not know about cervical cancer, or oropharyngeal cancer, do you have any messages for them? For example, do you have a pamphlet? Do you have any information? You can share it.

You can email me. I will discuss it. It is in process.

It has not been finalized yet. I will consider it as a draft. It is just for my own personal consumption.

When you are interviewing people, because they have kids and children, they are concerned. If they want to get it done, how should we... Very true. It is like a lamp.

Whatever you say to someone, it spreads to others. It is still being worked on. I don't have an expert opinion on it.

Sir, your program will start in 2025. It will take three years. In 2026, we will do it in KP.

In 2027, we will do it in Balochistan and GB. When you talk about provinces, does the strategy change? KPK and Balochistan? Rural areas or urban areas? As such, there is no strategy change. But definitely, lessons learned from each year.

Lessons learned in 2025. We will improve it. But province-wise or sector-wise, rural or urban, people are dealt with the same way.

Awareness-wise or sensitization-wise. There is no difference. There is a slight difference.

In the sense that, when we look at social media, or media, which media is in which area, which media is running, we have so much leverage that we do things according to the media. So, availability... The strategy is the same. The strategy for every area is the same.

So, the availability for the last three years is confirmed. It hasn't been discontinued. No, no.

The plan has been chalked out. Budgets are available. Provinces agree on it.

They want to support routine immunization. So, that is... Our experience so far is that in the last ten years, we have introduced six vaccines. So, we have never had any such issue.

So, there has been no issue Availability in terms of vaccines, in terms of finances. Although we are taking vaccines from Gavi, but the government of Pakistan or the respective provinces have to give their due share to ensure the availability. So, we have been getting our due share in the last three years.

Right. But it is expensive. It is not that expensive.

How much is it expensive? It is not that expensive. And in this case, the point-in-time that we are getting from Gavi is 1-3. Let's say, if it is a 3-rupee vaccine, then we have to give 1 rupee to Gavi.

Right. So, will it be like this for all three years? Or will it be like the phases of vaccines in Pakistan? We definitely have to go but we are expecting that by that time, the vaccine will be cheaper. The cost is also increasing, but at the same time, the cost of the vaccine is also reducing.

Right. Sir, are we ready to receive new vaccines in terms of logistics? Yes. We have a state-of-the-art code-chain system starting from federal up to health facility level.

Just for your information, in the last campaign, in 2022, we targeted more than 90 million children. So, our capacity is 90 million. At one point in time, we can accommodate 90 million vaccines.

Here, it is 11 and 12 million. It is 11 million with some wastage. If you say 12 million, we have to address that.

So, the code-chain is not an issue. Same is the case with logistics. But the system is in place.

Already, we are addressing the 12 diseases and 8 to 10 vaccines. So, the system is in place. It has a synergetic effect.

It is like an add-on. Sir, for outreach, the fixed-point campaigns that you mentioned… Thank you, sir. Thank you.

Thank you so much. So, it will happen with time. Thank you so much.

Sir, the fixed-site campaign modality that you mentioned that your team will be there on the fixed site. Through announcements, people will come to that specific point and get vaccinated. The polio model will also be replicated.

We are ready for that. Our logistics team and logistics infrastructure are all supporting us. We are prepared for that.

Sir, what do you mean by polio structure? What do you mean by polio structure? Okay, sir. I don't know the polio structure. There is no to-do remuneration.

You said that you will not do it. Yes, I said that. Okay, coming back.

As you said, there is no announcement on the fixed site. But everyone knows that this is the fixed site. A patient is coming with his company.

So, we will… The fixed site will be the already existing healthcare facility. Yes. The EPI center is there.

People are going to get vaccinated. Okay. The rest of the things will be on your outreach and mobile.

Announcements, mosque announcements will be there. And the difference between our school and polio and our difference is very big. In a sense, we don't follow the polio model.

In a sense, we don't follow polio. You knock on the door, give them a drink and leave. We don't do that.

We have told you before that the team sits there. This is your whole union council. My social mobilizer will knock on every house.

The target population will come to this point. It is because as madam said, the cold chain will be maintained. We can't go to every house and open it.

It is injectable. Although it is a single dose, we can do it at the door. In the context of safety measures, I am not standing at the door and injecting.

To ensure safety measures, we will sit at one place and get our people to move. They will knock on the houses and come to this point. This is the difference between polio and us.

This is for STEAM. Since this is a measles rubella, we did a polio test on it. We also did an age cohort test on it.

I don't see a male or female in this. We also tested male and female. This is a parent's reference slip, which the social mobilizer will be using.

This is for that. This is for missed children. This is for first level supervisors.

This sheet will go to the first level. This will be added to the team. This will be the consolidated report of the UC.

This is for the different AFI. This will be the supervisor's checklist. This will be the rapid convenience assessment.

We will also check the coverage. We will also check the coverage of that area. We will take corrective measures accordingly.

[Speaker 2]

Sir, if I ask you in terms of availability, accessibility and affordability. So, we are ready in all these three terms with regards to the HPV vaccine.

[Speaker 1]

We can say that the availability for routine vaccines will be available.

[Speaker 2]

And after this pilot project, which starts from 2025 to 2028. Then afterwards, it will be the part of routine immunization. The part of EPI that will be made will be after this phase.

[Speaker 1]

Why did you use the word pilot? It's not pilot. It's actually 100% rollout.

[Speaker 2]

Sir, what I mean to say is that the immunization cards are running in a sequence. The parent without asking second question. When will we reach that stage?

We will reach there when it becomes part of that card. And a routine sequence will start. In which the receptiveness of our community will reach the stage of parents.

That they will take it as a routine immunization.

[Speaker 1]

You are asking me for a prediction.

[Speaker 2]

Okay.

[Speaker 1]

What should I answer? Has this thing been introduced in our R.I. yet?

[Speaker 2]

Sir, as we have introduced Rota. Rota is now also included in the immunization card on your website.

[Speaker 1]

We will have to think about it. That card is addressing 6 visits. If you want to do 6 visits.

If I know about the first visit, I will be able to address the second visit. If I know about the second visit, I will be able to address the third visit. It is addressing 6 visits.

That's why it is becoming a card. We are still thinking about it. It may be a single card.

[Speaker 2]

Because there is a lot of age gap in it.

[Speaker 1]

Age gap is also coming in it. And when we go into the routine, which I told you 9 to 14. In the routine, we will only address the 9-year-old girl.

One single dose. For that, we will have to do something further. That we will not be using this card.

Okay. This card is of your child's room. There is a time factor in it.

There is a number of visits. There is no such thing in it.

[Speaker 2]

It is a single dose.

[Speaker 1]

It is a single dose. For its record purposes. Maybe a single.

[Speaker 2]

We will certify it.

[Speaker 1]

Yes. It can be a card like this.

[Speaker 2]

Signed stamp card. Yes. It can be a card like this.

[Speaker 1]

It is not a retail card.

[Speaker 2]

Sir, thank you very much for your insight. One thing. Sir, it is written here.

The brochures you are making. What term are you using for Urdu? For cervical cancer.

But some people asked us. I mean, when we understand such a parent. Education wise.

[Speaker 1]

We have not used any. We have not reached to Urdu. First English will be made.

Because we are trying to understand people. If there is any question. Please note it down.

I will check and tell you.

[Speaker 2]

Sir, one more last question. I will not ask more than you. I think it is more relevant.

There is no unisex in it. Do we have contextually relevant tools. To study these drivers.

Of vaccination behavior. Social drivers.

[Speaker 1]

It is a proper questionnaire.

[Speaker 2]

They have adapted their questions. And locally. Contextually.

We have adapted it. And there will be surveys on it. And the results will be published.

[Speaker 1]

Definitely. As I said. Today we will present preliminary results.

[Speaker 2]

Sir, this figure of 11 million. Sir, this is the figure of 11 million. This is taken from our census.

[Speaker 1]

This is 9 years to 14 years cohort. This is 11 million. Punjab, Sindh, AJK and Islamabad.

Overall, if we talk about the whole of Pakistan. It is around 18 million. 17.5 million. 17.5 million.

[Speaker 2]

Less than 18 million.

[Speaker 1]

And it is being added on. Year is also being added on. In 2026.

Something else will be added. Our next 9 year age cohort. It is 2.9 million. 2.7 to 2.9 million.

[Speaker 2]

If we do age brackets. 9 years.

[Speaker 1]

No. For routine. How much will we have to do every year?

[Speaker 2]

How much will we have to add?

[Speaker 1]

2.9 million. We did a campaign. And added 11 million.

After that, we did not sit comfortably. Every year.

[Speaker 2]

In the routine.

[Speaker 1]

What you are doing in the routine. How many cohorts will you be doing?

[Speaker 2]

Last year, the remaining 8 years. 9 years.

[Speaker 1]

It will be done. It is 2.9 million.

[Speaker 2]

One more thing was coming to my mind. There will be a lot of money spent on it. Resource intensive program.

To what extent. Compulsion. How stressful will the government take it?

For polio. We do a lot. You have to get it done.

Missing children. We have to ask. Have you been vaccinated?

No. To what extent?

[Speaker 1]

Different provinces. We know. It is a devolved subject.

Every province is doing its own. At the national level. We can say.

Vaccination is the right of every child. But in the context of compulsion. Kp.

The state has approved its immunization bill. The state has approved its immunization bill.

[Speaker 2]

how to tell and how to explain that we are going to administer the vaccine. Obviously, whoever comes with the child, he will ask questions, he will get the information.

[Speaker 1]

The concept of cancer will be explained to him.

[Speaker 2]

No, no, the training will be given to the vaccinators.

[Speaker 1]

See, there is a complete operational guideline and these are the guidelines for the supervisors. In this, each and everything is there, including ACSM component and there is a separate module of IPC. Interpersonal communication.

Thirdly, then our team, the vaccinator, for that, in Urdu, as it is in English, our R is in Urdu.

[Speaker 2]

Sir, last question, will we work on LHVs or LHWs or is it basically your team?

[Speaker 1]

No, no, it will not be our team. LHVs will be on your fixed site and LHWs will play the role of social mobilizers, who will knock on the doors.

[Speaker 2]

And with their help, when you roll out in the community, they will be the part of it?

[Speaker 1]

They will be the part of it, as social mobilizers and those who know how to vaccinate, at times they will be for vaccination.

[Speaker 2]

Thank you very much, sir, for your valuable time. We are really grateful, sir, for this.

[Speaker 1]

Thank you very much. Done. Complete.

Based on the in-depth expert interview with **Dr. ….**, a technical officer involved in HPV vaccine rollout, the following **qualitative thematic coding** was conducted using the **WHO BeSD Framework**. Each domain is broken down into **codes**, **sub-themes**, and overarching **themes**, capturing structural, strategic, and social insights crucial to understanding HPV vaccine acceptance in Pakistan.

**1. Thinking and Feeling**

| **Codes** | **Sub-Themes** | **Themes** |
| --- | --- | --- |
| Suppression of sexual transmission element in messaging | Message framing for acceptability | Emotional sensitivity in health communication |
| Emphasis on cancer prevention to enhance message appeal | Benefit-oriented framing | Focus on disease severity and fear appeal |
| Community may Google HPV; exposure to unknown information | Perceived knowledge risk | Information-seeking behavior |
| FAQs and helpline prepared for parental concerns | Structured support tools | Preemptive clarification mechanisms |
| Sensitivity about girls’ age and gender in messaging | Age-appropriate education | Concern for youth comprehension and reactions |
| Fear of cancer mentioned in community | Illness fear as motivator | Emotional driver for acceptance |

**2. Social Processes**

| **Codes** | **Sub-Themes** | **Themes** |
| --- | --- | --- |
| Involvement of Lady Health Workers as social mobilizers | Community engagement | Trust-based health communication |
| Female staff prioritized for vaccination delivery | Gender-congruent interaction | Cultural acceptability in healthcare |
| Engagement of religious leaders for myth correction | Use of influential figures | Addressing religious-based vaccine hesitancy |
| Community trust leveraged via mosque announcements | Local norms in outreach | Integrating vaccine efforts with cultural norms |
| School-based sessions and outreach for out-of-school girls | Institutional and community outreach | School-community collaboration |
| Encouragement of parental permission and slip systems | Structured parental involvement | Social and familial consent processes |

**3. Motivation (Intent)**

| **Codes** | **Sub-Themes** | **Themes** |
| --- | --- | --- |
| “Only vaccine that prevents cancer” | Uniqueness of HPV vaccine | Positive reinforcement of vaccine utility |
| Experience with past campaigns builds confidence | Trust through precedent | Motivation based on prior successful campaigns |
| Emphasis on routine integration after campaign | Normalization of vaccine behavior | Intent to routinize HPV vaccination |
| Use of educated parents' Google search behavior | Information empowerment | Proactive motivation among informed parents |
| Promotion of vaccine among health professionals | Internal motivators in system | Championing from within health system |

**4. Practical Issues**

| **Codes** | **Sub-Themes** | **Themes** |
| --- | --- | --- |
| Three-pronged delivery (fixed, outreach, mobile) | Flexible service delivery | Context-adaptive operational planning |
| Social mobilizers direct children to fixed site | Human resource utilization | Efficiency in outreach without compromising safety |
| Vaccine safety protocol due to injectable form | Service safety requirements | Injection-based logistics & cold chain demand |
| Vaccine delivery not feasible door-to-door due to injection | Structural constraints | Limitations of house-to-house model |
| Operational guidelines include ACSM and IPC components | Integrated training frameworks | Preparedness in communication and counselling |
| LHWs assist with mobilization, not core vaccinators | Role clarity in workforce | Defined division of labor |
| Routine immunization to follow campaign success | Sequential strategy | Sustainability through phased transition |
| Availability and cold chain fully established | Infrastructure readiness | System capacity and preparedness |

**Summary Table of Themes Across WHO BeSD Domains**

| **BeSD Domain** | **Core Themes Identified** |
| --- | --- |
| Thinking and Feeling | Emotional framing, perceived cancer threat, structured communication support |
| Social Processes | Gender sensitivity, role of LHWs, cultural integration, institutional partnerships |
| Motivation (Intent) | Unique value of vaccine, lessons from past campaigns, drive to normalize behavior |
| Practical Issues | System readiness, outreach strategy, service constraints, logistic adaptab |
| **Analysis of IDIs & FGD to highlight facilitators and barriers of HPV vaccine uptake according to BeSD Framework**  Thinking and Feeling  Social Processes  Motivation  Practical Issues   \| **Respondent** \| **Facilitators** \| **Barriers** \| \| --- \| --- \| --- \| \| **Parents** \| \| \| \| **1** \| T   - **Belief in benefits of vaccination:**   *“Vaccines are a very good thing and should be given… these vaccines are good for the health of our children.”*   - **When explained trusts medical advice :**   *“….we saw on TV and heard in the news that this is a good thing, then we got them vaccinated.”*   - **Change in health behavior after personal experience:**   *“We did not get COVID vaccination for a month… Then we realized that we had made a mistake. We went back and got them vaccinated.”* \| - **Fear of side effects, especially infertility:**   *“Sometimes, people think that the growing girls won't be able to become pregnant later on…”*   - **Exposure to misinformation during COVID:**   *“There were a lot of rumors that we should not be vaccinated.”* \| \| S   - **Trust in schoolteachers:**   *“Yes, the children listen to the teachers. The children have full confidence that what the teachers are saying is good for them.”*   - **Daughters counseling parents on health-related issues:**   *“My elder daughter explains to us what she learns at school… There are a lot of things that our children have told us.”*   - **Potential for community-level education:**   *“Doctors should make a team. They should hold a conference. They should explain (about HPV vaccine) to everyone… That this is a good thing for your daughters.”* \| - **Household decision-making controlled by elders:**   *“My mother-in-law. She has people she trusts. So she gives a better solution.”*   - **Influence from surrounding community beliefs:**   *“The Pathan do not get vaccinated… They say that this is madness. They should not be vaccinated.”*  *“People will think bad about it(HPV vaccine)… They say, if we are okay now then it is fine.”* \| \| M   - **Conditional acceptance with medical endorsement:**   *“If it is good for the girls, we will get the vaccine.”*   - **Proactive willingness after understanding the disease:**   *“We will definitely accept it. If it is good for us. We will discuss it with people around and our family members.”*   - **Empowered girls influencing household norms:**   *“She (the daughter) says, MashaAllah, you should speak for your rights. Why are you quiet so far? You are doing this wrong.”* \| - **Hesitation until full trust is established:**   *“You have put it in my mind. I will have to think about it.”*   - **Preference to depend on experts for decision-making:**   *“It's better to consult a doctor first and then see what we can do about it.”*   - **Need for validation from others:**   *“If we take a decision on our own, we get double mind whether it's right or not.”* \| \| P   - **Reliable routine visits by vaccinators:**   *“They (vaccinators) have adequate stock and they come twice in a week.”*   - **Support for school-based vaccination:**   *“Even if the doctor goes to the school and does the awareness programs with the girls and teachers, it will be better.”* \| - **Rural health practices perceived as neglected:**   *“When I was in my village before marriage, there was no practice… Doctors didn’t use to go and were afraid to go there.”*   - **Dependence on doctor consultation may delay action:**   *“I will talk to my doctor… then we will definitely get it.”* \| \| **2** \| T   - **Open to vaccination when informed:**   *“… this vaccine will be an effective way to either completely get rid of this cancer or improve her quality of life, so I have no issue using this vaccine for my daughter.”*   - **Values scientific evidence and prior success stories:**   *“If we get some stats, it will be helpful for me to make a decision... We welcome the positive effects.”*   - **Understands analogy with COVID vaccine acceptance:**   *“...when people around them started getting vaccinated, and they saw that there were no side effects... they got vaccinated happily.”* \| - **Concern about long-term side effects:**   *“When she turns 40… then the body's immune system also weakens. So, what are the long-term effects?”*   - **Skepticism until sufficient evidence is reviewed:**   *“Before going to have that vaccine, I would personally like to do some research.”*   - **Perceived unknown hormonal risks:**   *“It's just hormonal changes, I can't think of anything else. Other than that, it's female-related issues which females know better.”* \| \| S   - **Belief in doctor-guided decision-making:**   *“We will go to an expert gynecologist to know more… may have a second opinion from a general physician as well.”*   - **Recognizes key role of mothers in convincing males:**   *“So the starting point is the mothers. Only if mothers will be convinced they will be able to convince the fathers…”*   - **Supports awareness through respected/influential figures:**   *“Influential people… members of national assembly, provincial assembly… if they work collectively for this cause only then will people understand quickly.”* \| - **Cultural authority of imams in rural settings:**   *“Agar imam sahab nay keh diya lagwa lain to lagwa liya, agar unhon nay kaha kay haram hay to phir who haram ho jayay ga.”*   - **Gendered communication norms:**   *“These conversations are more between mothers and daughters… fathers don’t talk about these things with 15 or 16 years old.”*   - **Fear of stigma around unmarried girls:**   *“If any slightest issue happens, they label the girl’s life being ruined completely.”* \| \| M   - **Accepts girls’ autonomy in decision-making:**   *“It is compulsory to take their permission… although we can do that but it is compulsory for us to discuss it with them.”* \| - **Vaccine Hesitancy without visible examples of disease cases:**   *“If they have not yet encountered such a case... I don’t think anyone would agree.”*   - **Resistance to HPV vaccine uptake without effective communication of knowledge:**   *“Somebody having no prior knowledge of this disease, if he would be directly pitched, he would not be interested…”* \| \| P   - **Prefers government-led initiative:**   *“Government of Pakistan initiates this campaign itself. It is spread through mainstream media…”*   - **Recommends localized doctor engagement:**   *“If doctors will work in their own areas under the initiative of government… that would be more beneficial.”*   - **Supports schools and colleges as outreach venues:**   *“Girls colleges, women universities… seminars can be conducted and girls can be counselled…”* \| - **Demand for evidence before action:**   *“We need to do some homework ourselves… so that we don't get into any complications.”*   - **Skepticism with regards to cooperation from school bodies:**   *“Schools would not own the decision of actually administering the vaccine so government will have to take the initiative…”*   - **Fears over cooperation from private educational institutions:**   *“Even on slightest issue their reputation is tarnished…”* \| \| **3** \| T   - **Belief in vaccine benefits:**   *“I say that it should be introduced…”* *“If you arrange the seminar, we will definitely attend it, we will listen, so that we get the awareness.”*   - **Trust in doctors to consult for daughter’s condition:**   *“I will definitely ask her(Dr) whether to get it done… because she knows all her cases.”* \| - **Fear of side effects from previous vaccination experience (COVID):**   *“We all have a lot of pain in our muscles. We have a lot of pain in our bones. We have a lot of pain in our eyes.”*   - **Husband’s refusal to COVID vaccine for the daughter due to iron deficiency anemia and hormonal imbalance:**   *“She was already getting her treatment, so we didn’t get her vaccinated thinking she does not get any other problem.”*   - **Belief in poor quality of freely provided medicines:**   *“People conceal real or quality things and cheat others here so people are afraid.”* \| \| S   - **Trust in institutional doctors and social sharing in workplace:**   *“I am working in an institution, we share with each other.”*   - **Active community engagement through health camps:**   *“In our area, whenever a trust sets up a health camp… people come… it works well when there’s a clear message.”* \| - **Dependent on husband's approval despite personal acceptance:**   *“If I don’t get the permission from home, then I can’t do anything.”*   - **Distrust in free services:**   *“If they are given something free of cost, they think they are being tested.”*   - **Community refusal (e.g., ethnic resistance to vaccination):**   *“Most of them were Pathan… They said that they don’t get it done.”* \| \| M   - **Would act positively if informed and supported:**   *“I will get it done, because it is for my daughter’s safety, she is a girl.”*   - **Motivated to act on trusted doctor’s recommendation:**   *“Now, I will definitely consult with the doctors… I will definitely ask her whether to get it done…”*   - **Motivated by personal knowledge of cancer:**   *“There was a teacher in my school. She had ovarian cancer and had it removed. I have seen this with my own eyes.”*   - **Acceptance of seminars and structured awareness efforts:**   *“We will definitely attend it, we will listen, so that we get the awareness.”* \| - **Health complications of child delay decision-making:**   *“...her periods were not stopping… there was a lot of blood deficiency… that’s why we didn’t do it(COVID vaccine).”*   - **Influence of misinformation post-vaccination:**   *“There were a lot of videos on YouTube… that those who will get it done, will die in two years…”* \| \| P   - **Access to cooperative staff and functional health system:**   *“Our doctors are also always very good, we get all the information from here, and they guide us well.”*   - **Positive experience with government vaccination drives:**   *“There was a very good system… tents, chairs set up… the health workers were also from the government. And they used to cooperate.”*   - **Recommends community-based health camps for outreach:**   *“...you can organize sessions there… give talks… If you coordinate with a relevant doctor… many people will come.”* \| - **Mistrust in medicine quality:**   *“People conceal real or quality things and cheat others here…”*   - **Negative past experience in government facility :**   *“My BP was high… waiting so much… very bad experience… I got all my treatment done in private.”* \| \| **4** \| T   - **Positive attitude toward childhood vaccination:**   *“In my view, we vaccinate our children to protect them from any future deficiencies or disabilities.”*   - **Motivated after hearing real stories of young girls with cancer:**   *“There were girls as young as 14 and 15 who were having their uteruses removed… Hearing that was truly painful and eye-opening.”*   - **Receptive to new information about HPV:**   *“No, no. I have not heard about it. But definitely, if it is a disease, then to know about it is necessary.”* \| - **Cultural discomfort about sexual transmission:**   *“It should only be given with that specific purpose in mind, not for any other reason.”*   - **Lack of prior awareness about cervical cancer or HPV vaccine:**   *“No, I have just heard about it… I have not heard that a vaccine is available.”* \| \| S   - **Trust in family health professionals:**   *“My sister-in-law is a nurse, so I would talk to her about it.”*   - **Cooperative family dynamic:**   *“My family is very cooperative and they will support me.”*   - **Support for religious scholars in awareness campaigns:**   *“To correct the misconception that these vaccines are from non-Muslims… religious scholars can play a better role.”*   - **Support for teachers, doctors, and Ulma working together:**   *“Doctors and teachers can also play an effective role…”* \| - **Peers resisted polio vaccine due to religious misinformation:**   *“She said that it is just that this vaccination is from non-Muslims… it will take our children towards disability.”*   - **Has the opinion that public acceptance will be gradual:**   *“Some people will accept it and support it right away, while others will be hesitant.”* \| \| M   - **Strong protective maternal instincts:**   *“As mothers—and I myself have a daughter—we naturally care. If there is something that can help, then it is worth considering.”*   - **Open to exploring more information:**   *“When I talk to you, you tell me that there is vaccination for cervical cancer… how is it administered?”*   - **Desire for seminars for awareness before vaccine is launched:**   *“Seminars should be organized… awareness has to come first.”* \| - **Not previously aware of the vaccine despite being educated:**   *“I only learnt about it recently myself.”*   - **Cultural readiness not immediate:**   *“People might come around eventually, but I do not think it will happen quickly.”* \| \| P   - **Good experience with hospital vaccinators:**   *“ They talk to you in a very good way… I have a very good experience with them.”*   - **Prefers multi-channel outreach (seminars, social media, schools):**   *“Multiple platforms need to be used. The message should be spread in various ways…”*   - **Sees role for Religious Affairs Ministry and scholars positively:**   *“They can conduct seminars… most of them are Ulma coming from the Madaris.”* \| - **No prior communication by vaccinators about what they administer:**   *“They just look at the card and apply it… he does not tell you anything.”*   - **Missed opportunity for in-depth counseling:**   *“No, they do not inform us what the vaccine is for.”* \| \| **5** \| T   - **Positive perceptions regarding vaccination programs:**   *“So I think vaccination is a protective measure… if the government is investing so much… then positive thinking should be that they want to give a facility…”*   - **Belief in health responsibility from a faith-based perspective:**   *“Islam is a religion of nature so we have not been stopped from doing this.”*   - **Real-life experience of disease burden influencing her thinking:**   *“My sister-in-law developed a cyst… it progressed so quickly that she had to have her uterus removed… now she’s a heart patient and diabetic.”* \| - **Suspicion over vaccines as a population control tool:**   *“They may be trying to reduce the birth rate of Muslim children.”*   - **Personal loss linked to vaccine hesitancy:**   *“My child recovered from disease quickly… but he had meningitis and could not survive. After that, we didn’t give importance to vaccination.”*   - **Cultural myths leading to fear (iodized salt):**   *“My teacher once said this is also being used to control the birth rate… the family planning sector was promoting iodized salt.”* \| \| S   - **Cultural norm to protect vaccination cards:**   *“Illiterate mothers are also getting vaccinated… They keep their cards safe.”* \| - **Familial resistance and lack of prioritization for routine vaccines:**   *“Our family is a religious family… these things are not given much importance.”*   - **Fear of stigma around sexuality and age group:**   *“They will say… you are taking this vaccine before marriage. This is even worse. They will say, this is exactly what we were saying.”*   - **Teachers and religious leaders' mixed influence:**   *“People with strong religious beliefs turn to scholars… some in favor, some against.”* \| \| M   - **Openness to act if intention is clear and trust is built:**   *“If you want to do it… and your purpose is to serve your people… then sincerity is the source of guidance.”*   - **Support for school-based interventions and long-term strategies:**   *“Like polio, even now the campaign is still running successfully… that shows when something is done with sincerity, people support it.”*   - **Belief in early intervention after observing disease cases:**   *“If one in your family has gone through it, that one person is also equally important.”* \| - **General hesitancy after personal experience with child death:**   *“After that [loss of child], my husband and I did not give importance to vaccinate the rest of the children regularly.”* \| \| P   - **Appreciation for government vaccination system:**   *“You are given so much importance… in hospital, they have made a place at the reception… we get the first dose immediately and take the baby home.”*   - **Perception of government doctors as experts:**   *“Although people say there is a lot of inconvenience in government hospitals… doctors are experts.”* \| - **Doubts about why vaccines are free while medicines are expensive:**   *“When we take medicine… we have to pay. But vaccination is free everywhere. People are asking why this is happening.”*   - **Questioning public health priorities:**   *“Why is the government spending so much on vaccination, but not on other health problems?”*   - **Limited explanation at point of vaccination:**   *“It’s written on the card… but no one really tells you what it is or why.”* \| \| **6** \| T   - **Supportive of vaccination against cervical cancer:**   *“Yes, absolutely…it should be allowed.”*   - **Acknowledges rising cancer burden among youth:**   *“Because there were very rare cases before. Now I think we are hearing about the second, third, fourth cases… And it is more among young people.”* \| - **Concern over side effects, especially menstruation-related:**   *“We want there to be no side effects on periods.”*   - **Fear from previous COVID vaccine side effects:**   *“They say that since we got vaccinated, we have had a lot of body aches.”* \| \| S   - **Trust in schools and healthcare staff as messengers:**   *“Schools are doing good job… girls are given separate sessions regarding menstrual hygiene.”*   - **Girls trust information from teachers:**   *“Girls take words of teachers very seriously.”*   - **Father identified as key influencer in joint families:**   *“Father… if consulted, can convince others.”* \| - **Community may associate vaccine with hormonal or health disruptions:**   *“As girls’ periods are irregular… they already have health issues… So people may worry.”* \| \| M   - **Clear intent to vaccinate daughters:**   *“Yes, absolutely. We will try to get them vaccinated.”*   - **Belief in early preventive and protective value of vaccine:**   *“They should be told that this thing is spreading very fast… there is a vaccine which can be used to be safe from it.”* \| - **Risk of rejection if source is not trusted or information not well-explained:**   *“Majority of people approach the negative points….”* \| \| P   - **Experience in the health sector supports trust in procedures:**   *“Students seriously listen to us.”*   - **Institutional channels for awareness (school-based sessions):**   *“Their should be health sessions like those where girls are taught to carry menstrual kits…”* \| - **Concern about introducing new vaccines without prior sensitization:**   *“They will need awareness… because this vaccination is a start-up.”*   - **Need for clarity about vaccine benefits mostly:**   *“They should not be told about the side effects.”* \| \| **7** \| T   - **Belief in vaccination as prevention against illness:**   *“Vaccines are important so that there's no fever or kaali khansi, or khasra or any deadly illness or disability.”*   - **Confidence in the purpose of vaccines:**   *“I say that this work should be done … this is a protection vaccine(HPV) for your life.”*   - **Supportive attitude after seeing disease burden (e.g. cervical cancer):**   *“I think it should be given. Because this disease(cancer) is spreading a lot… this should be done for your life.”* \| - **COVID-related misinformation (vaccine causes death):**   *“I was being told… you will die in two years. Get the vaccine in two doses and then, you will die.”* \| \| S   - **Influence of educated women (daughters-in-law) on health decisions:**   *“My daughters-in-law… are getting vaccinated. They know when to go to the hospital.”*   - **Positive attitude toward school vaccination programs:**   *“Yes, I did give permission…and the children got vaccinated.”*   - **Teachers seen as trusted messengers:**   *“If the school does it, then the children will also be satisfied.”* \| - **Family male resistance to vaccination due to mild adverse events:**   *“His father said not to vaccinate him… because of fever and diarrhea(after previous vaccination).”*   - **Community-specific resistance (ethnic group):**   *“Some are from Afghanistan, Pathan, they don’t even give polio drops. They say, our husbands don’t let us get it done.”*   - **Family resistance due to past adverse event (child developed a lump):**   *“Once my child got a lump after vaccination… my father-in-law said we are not going to get vaccinated.”* \| \| M   - **Willingness to vaccinate if advised and awareness spread:**   *“If it is free and there are no problems in it… then clearly, if the doctor is giving such advice then it must be right.”*   - **Belief in religious responsibility and trust in Allah’s will:**   *“Death and life are in the hands of Allah… But if it is helpful, then we should take it.”*  *“We get our children vaccinated and give them polio drops… Alhamdulillah all the children are fine.”* \| - **Need for family approval (especially parents):**   *“It will be up to their parents… then maybe the girls will also be satisfied.”* \| \| P   - **Free vaccination seen as beneficial:**   *“Yes, if it is free of cost, it is a good thing.”*   - **Good experiences with public vaccination services:**   *“There is a lot of co-operation… they guide us properly.”* \| - **Dependency on convenient timing with regard to outreach services:**   *“They are also good if we go within their timings.”* \| \| **8** \| T   - **Strong belief in vaccine efficacy from experience:**   *“Vaccinated children fall sick very less… Thank God that my children did not fall ill.”*   - **Trust in government-backed health initiatives:**   *“If something is coming from the government… it must be proven… it is a very good thing.”*   - **Desire to protect daughters from cancer:**   *“Cancer is very common now… I think girls should get vaccinated.”* \| - **Concern about side effects:**   *“When I hear this for the first time, I will ask if it has any side effects… If it has benefits, then what are the negative effects?”*   - **Impact of COVID-19 vaccine fears:**   *“As soon as the corona vaccine came, there were so many issues… people said it makes you weak.”* \| \| S   - **Positive influence from peers and family:**   *“My sisters, my sisters-in-law are all getting their children vaccinated.”*   - **Teachers and school as trusted channels:**   *“My children are told that they have been vaccinated at school… I don’t have any hesitation.”* \| - **Anticipates resistance from elders in joint families:**   *“It will be difficult at first… There are so many rumors associated with polio drops.”*   - **Community-level ignorance or suspicion:**   *“This is Pakistan. It is very difficult to satisfy them… This is ignorance.”*   - **Endorses awareness through doctors and schools:**   *“We will consult our family doctor… They should tell people through school too.”* \| \| M   - **Personal motivation to vaccinate:**   *“If our children get safe from diseases, why won’t we get them vaccinated?”*   - **Positive past experiences with vaccines:**   *“I have also been vaccinated against COVID-19 and my children have also been vaccinated.”* \| - **Concerns if vaccine is not well-explained:**   *“We will ask the doctor. We will get to know. Then we will decide.”*   - **Conditional motivation based on guidance from trusted sources:**   *“We will consult our family doctors… Because we are not doctors ourselves.”* \| \| P   - **Regular engagement with EPI centers and satisfaction:**   *“I got them vaccinated from the EPI center… If there’s any problem, they help us.”*   - **Encourages free HPV vaccine:**   *“If it is free, people will get vaccinated… This is a very good thing.”* \| - **Demand–supply gap observed occasionally:**   *“If the vaccine is not present, they tell us the date and we come back.”* \| \| **9** \| T   - **Belief in childhood vaccines benefits:**   *“It is necessary to protect you from various diseases.”*   - **Willingness to accept new vaccines if evidence of its prior use provided:**   *“If people have been vaccinated in other countries… we will definitely get vaccinated.”*   - **Awareness of cancer risk from family exposure:**   *“Some people in our family have had it(cancer). So, it is dangerous.”* \| - **Menstruation and fertility-related fears:**   *“There should be no problem with the child's menstruation or with pregnancy.”*   - **Fear of fake or ineffective vaccines:**   *“We will get it from a certified institution… sometimes it happens that medicines are fake.”* \| \| S   - **Family doctors trusted for final decision:**   *“If the doctor assures us that it is safe… we will definitely get vaccinated.”*   - **Maternal empowerment in vaccine decisions:**   *“I make health related decisions most of the time… I used to take my children to the doctor.”*   - **Mothers as mediators for fathers:**   *“Men should be targeted by their families…females should educate their husbands.”* \| - **Societal resistance to new vaccines:**   *“It is difficult in our society… people do not accept it.”*   - **Fear of widespread rumors about infertility:**   *“They will say… there will be no children later on… such problems increase in our country.”* \| \| M   - **Clear personal intention to vaccinate daughters:**   *“Yes, I would like to get her vaccinated.”*   - **Open to adopting if benefits are explained and vaccine is safe:**   *“If it is for our benefit… and it is safe… then we should be a part of it.”* \| - **Wants health worker authentication before proceeding:**   *“We will get it from a center where the doctor will make sure that this is a real vaccine.”*   - **Distrust of school-based vaccination programs:**   *“People are concerned—who is applying it? Where did it come from? So I don’t think this will be very effective.”* \| \| P   - **Experience of both public and private sector vaccination:**   *“We got a daughter vaccinated from a government hospital… it was good.”*   - **Support for free vaccine provision through public centers:**   *“It should be given free of cost… If it is very expensive… people cannot afford it.”*   - **Endorses social media, hospitals, and cancer centers for outreach:**   *“Awareness should be started in cancer wards… through doctors and social media.”* \| - **Rural communities have low access:**   *“There is no such facility in our village… just a small health center where LHVs work.”*  *“… It was in the city only.”* \| \| **10** \| T   - **Trust in traditional childhood vaccines:**   *“We are doing it as a duty and it's okay… All the mothers were getting their children vaccinated and it was okay.”*   - **Belief in disease prevention:**   *“The purpose would be prevention… to prevent tuberculosis, measles, mumps, rubella.”*   - **Familiarity with cervical cancer and its seriousness:**   *“Yes, I have heard of it… I heard it from an Indian actress who wanted to raise awareness.”* \| - **Vaccine hesitancy from COVID experience:**   *“There were no studies… People were saying that they don’t know what will happen in five or two years.”*   - **Fear of being used for experimentation:**   *“We don’t want to experiment on ourselves.”*   - **Doubt about cervical cancer prevalence:**   *“I don't think there is so much cervical cancer in our society… I know a few young women who have died of breast cancer, not cervical.”* \| \| S   - **Culturally embedded childhood routine immunization:**   *“We never asked them. It was totally acceptable for us. It was a routine practice.”*   - **Sees women as influencers for male decision-makers:**   *“Only a spouse can do it. Mothers should educate their husbands.”* \| - **Male-dominated decision-making in society:**   *“It is the men. It is a male-dominant society. It is a macho society.”*   - **Conservative resistance depending on region:**   *“KP is more conservative. Punjab is not that conservative… It may depend on the area.”*   - **Skepticism from global figures reinforces hesitancy:**   *“A European tennis player quit the Olympics… he did not get vaccinated(COVID).”*   - **HPV vaccine uptake would depend on personal experience or family history:**   *“People with family history would be concerned… others would not take it seriously.”* \| \| M   - **Accepts preventive value for vaccines with proper information:**   *“If this thing is coming… then I think it is a very good thing.”* \| - **Reluctance to adopt new vaccines quickly:**   *“It will take time. We’ll think about it in 10 to 15 years. Not now.”*   - **Not convinced enough to consider HPV vaccine for herself or daughter:**   *“There are a lot of vaccines. But we don’t want to experiment on ourselves.”* \| \| P   - **Vaccinated children at private clinics without access issues:**   *“We got them vaccinated from our child specialists… I did not face any challenge.”*   - **Supports use of hospitals, and social media for awareness:**   *“Definitely social media. And definitely hospitals where many patients come.”* \| - **Lack of explanation from healthcare providers:**   *“We never asked them… They never told us what disease they are vaccinating against.”*   - **Gynecologists trusted by women:**   *“Women trust their gynecologists the most… they talk to them about everything.”*   - **Recognizes need for informed choice for adolescents:**   *“A child should be educated. He should know what he is doing and why he is doing it.”* \| \| **Healthcare Providers** \| \| \| \| **1** \| T   - **Strong preventive health mindset:**   *“I am working on preventive health… that we should cure the disease before acquiring it.”*   - **Aware of rising cancer cases and HPV relevance:**   *“Cancers are becoming very common.”* \| - **Low baseline awareness of cervical cancer and HPV:**   *“There are very few people who know about this thing… even educated people don’t know about cervical cancer.”*   - **Community does not prioritize health:**   *“We, the Pakistani community, give our health the least priority.”* \| \| S   - **Mothers and teachers identified as key influencers:**   *“Mothers and teachers are two categories that kids listen to.”*   - **Supports engaging extended family—especially grandmothers:**   *“If we want to convince the husband, his mother is the biggest factor… We can engage grandmothers.”*   - **Trust-based counseling with HCWs builds compliance:**   *“They trust me because they know I will not tell them anything which is not to their advantage.”* \| - **Male dominance in decision-making can slow acceptance:**   *“In our society, decision-making is usually done by fathers or males in the family.”*   - **Social resistance in conservative or rural communities:**   *“If you talk generally, there is resistance in families.”*   - **Fear of side effects and historical vaccine rumors:**   *“We have to convince parents… like there are so many myths about polio—that children will get infertility.”* \| \| M   - **Personally motivated to promote vaccination:**   *“Why not I save them [nieces] by creating awareness…”*   - **Role model behavior as trusted source in family:**   *“They call me first if they have any health issue… then they go to the doctor.”*   - **Sees potential for behavior change through logic-based counselling:**   *“If we have convincing power, and we talk logically, men can also be convinced.”* \| - **Hesitancy in parents:**   *“The first question will be… why are you doing this? Second, will there be side effects?”*   - **Need for targeted and relevant spread of information:**   *“We will have to be well prepared for all these questions.”* \| \| P   - **Experience in organizing vaccine awareness and subsidized drives:**   *“We conducted awareness and then vaccination camps for Hepatitis B… and got a very good result.”*   - **Strong network for outreach (e.g. community midwives, BHUs):**   *“We can target houses of community midwives… locate their areas… get data from district health officers.”*   - **School and institutional readiness to host sessions:**   *“We conducted health sessions monthly… invited expert speakers… emailed all departments.”*   - **Engaged in rural outreach and committed to expand:**   *“We have a second campus in a city… I’m planning a camp before the June holidays.”* \| - **Logistical challenges like stock shortages (COVID experience):**   *“There was shortage of COVID vaccine due to high burden… we have to estimate sample size before rollout.”*   - **Time constraints of working mothers:**   *“… working women have to manage time to attend sessions.”* \| \| **2** \| T   - **Strong support for prevention when vaccine is available:**   *“If the government is supplying and there can be lifetime immunity, then why not, it should be done.”* \| - **Believes cervical cancer burden is low in clinical practice:**   *“We are not seeing so much CA cervix, that we should put so much cost for it.”*   - **Expresses reservation about using new vaccines:**   *“If you think that I have got Meningococcal infection, so let me apply Meningococcal vaccine… your own antibodies will never develop.”*   - **Skeptical herself about getting vaccine for her own daughter:**   *“We ourselves have a lot of reservations.”* \| \| S   - **Endorses school-based education to reach families:**   *“Do school going girls counselling… they will take the message home.”* \| - **Anticipates social stigma due to STI link:**   *“It will become a stigma… you tell so much that it becomes stigmatized, that its transmission is going to happen.”*   - **Predicts cultural myths around infertility:**   *“Our social myths… girls will get infertility… and these issues are obvious.”*   - **Highlights that even doctors are hesitant to recommend:**   *“We have not told [patients] to start their daughters on the vaccine… because we are not on the same page yet.”*   - **Supports involving religious influencers:**   *“Specially our religious influencers… only then this will work.”* \| \| M   - **Willing to consider if data supports effectiveness:**   *“If we screen the whole population… and then do vaccination, then it will be a better option.”*   - **Believes that public will engage if presented with basic facts:**   *“Just tell them… this is a lifelong disability, it can lead to death… so prevention is better than cure.”* \| - **Delayed trickle-down of information and behavior change:**   *“It takes years, it takes years.”*   - **Skeptical of urgency without a national screening program:**   *“Before vaccination, we should have started the national screening program.”*   - **Lack of acknowledging the urgency of the problem:**   *“If CA was so common… why do we not have 50% patients in our ward?”* \| \| P   - **Suggests starting with awareness at PTMs and schools:**   *“You can target the parents in PTMs.”*   - **Advocates for printed brochures, digital resources:**   *“Give them printed literature, tell them sites, give them links.”* \| - **No structured national screening registry:**   *“Screening means… you are registered… every 3 years you get an appointment.”*   - **Screening and HPV genotyping expensive and inaccessible:**   *“HPV test costs about 6000 to 8000… not available universally.”* \| \| **3** \| T   - **Believes in vaccine effectiveness based on lived experience:**   *“We explain to the people that if the vaccine was applied on time, the child would not have been sick… People have started getting vaccinated on time.”*   - **Acknowledges fear, but sees counseling as a way to overcome it:**   *“We will tell them that this is a dangerous disease… cancer is spreading… then they may comply.”*   - **Role modelling an effective way to target fear:**   *“People say, after corona vaccine there has been changes in my husband… but we counsel them with examples from our own family.”* \| - **Cancer-related fear triggers avoidance:**   *“People will be scared of the name of cancer vaccine… like what happened when corona vaccine came.”*   - **Concerns about infertility and marriage prospects:**   *“People will also be scared that their daughter is getting married today or tomorrow… She may not be able to become a mother.”* \| \| S   - **Trusted community figure with deep rapport:**   *“We are just like their nani dadi… People laugh with me. I become a mother-in-law to them… they trust us.”*   - **Uses real-life community examples to encourage compliance:**   *“We show examples: this girl got vaccinated, and there was no harm.”*   - **Works with schools and teachers to ensure coverage:**   *“If you give guidelines to the teachers, they also tell the parents.”* \| - **Misinformation spreads via rumors:**   *“They thought… America is testing on our children. They didn’t get vaccinated.”*   - **Concerns over unknown volunteers replacing LHWs:**   *“When you don’t know the vaccinator… you worry whether hands are clean… Are my children safe?”* \| \| M   - **Deeply motivated and leads by personal example:**   *“First I vaccinated my child, then I told others. People trusted because I showed my own child.”*   - **Committed to community education:**   *“We consider our community like our own home.”*   - **Willing to vaccinate own daughter and support HPV rollout:**   *“We are given awareness and training, we will get it done first.”* \| - **Need to address parental fears and clarify rumors repeatedly:**   *“Parents will have questions… ‘Why are you doing this?’”*   - **Infertility concern being a persistent fear:**   *“Now people worry a lot about infertility.”* \| \| P   - **Experience in managing school vaccinations, covering absentees:**   *“We take the list in advance… then we cover those children.”*   - **Advocates for multi-pronged awareness campaigns:**   *“Go to communities, do sessions for men and women, use brochures, TV, mobiles…”*   - **Willingness to coordinate with NGOs for outreach:**   *“NGOs request us for sessions when they can’t work in uncovered areas.”* \| - **Lack of LHW coverage in high-population areas:**   *“In an area, population is 60,000… Only 4 LHWs cover 6,000 people.”*   - **In some areas, absence of trusted LHWs undermines trust:**   *“They ask us… why aren’t you working in our area?”*   - **Staff shortage limits geographic reach:**   *“We only work on a population of 2000… We don’t go beyond it.”*   - **Vaccine stockouts are common (e.g., meningitis, pneumococcal):**   *“Vaccines often shorten… then vaccinators write it and call later.”* \| \| **4** \| T   - **Strong support for HPV vaccine and cancer prevention:**   *“My gut feeling is that people will welcome it… it will be received positively.”*   - **Confidence that cancer is serious and rising:**   *“As much as cancer has been diagnosed, people have awareness that it’s a lethal disease.”*   - **Views vaccine as preventive and justified:**   *“If the government is doing this, then it’s a very good initiative.”* \| - **Mistrust triggered by social media content:**   *“Whatever comes on WhatsApp, people believe it blindly… especially elders.”* \| \| S   - **Trust in physicians as key influencers:**   *“If a doctor is your treating physician for 8–10 years… patients will do what you say.”*   - **Mothers seen as main decision-makers in child health:**   *“Mostly, mothers talk… and they are comfortable even with male doctors.”*   - **Fathers tend to comply when mothers are convinced:**   *“If you want to work in any house… then mothers can convince fathers.”* \| - **Social media misinformation spreads easily:**   *“60-year-old people form WhatsApp groups… if elders say something, families accept it.”*  *“People used to say kidneys are being removed, vaccine is water… they’re doing experiments.”*   - **Distrust persists in certain ethnic regions (e.g., KPK):**   *“Some far-flung areas of KPK, people say it’s not our trend, and just don’t vaccinate.”* \| \| M   - **Highly motivated to vaccinate his own daughter:**   *“Yes, I am definitely motivated… we clearly tell people the problem and solution.”*   - **Pediatricians and public doctors ready to lead awareness:**   *“Healthcare providers giving presentations at school will have a very positive effect.”* \| - **Caution against launching without awareness:**   *“If we launch the program directly, obviously there will be hesitancy.”*   - **Skepticism in low-literacy or rural settings:**   *“In rural areas… we will have to do a little more effort.”* \| \| P   - **Good infrastructure for existing vaccine delivery:**   *“No, there’s no issue. In government vaccines, there has been no shortage.”*   - **Supports school-based sessions and phased awareness:**   *“Most campaigns should be at school level… through doctors, for a positive effect.”*   - **Acknowledges EPI and private vaccination coexistence:**   *“At my private hospital, patients come for flu, typhoid… and we counsel them.”* \| - **Initial COVID vaccine rollout faced logistical strain:**   *“There were issues of demand and supply… because it was new.”*   - **Poor disease-specific awareness among general public:**   *“50% people don’t even know what disease a vaccine is protecting them against.”* \| \| **5** \| T   - **Confidence in vaccine efficacy when recommended by WHO:**   *“If it is considered safe by WHO, that means this vaccine is safe.”* \| - **Skepticism from both public and professionals during COVID:**   *“Even educated people were hesitant… some doctors said it causes heart attack or cardiac arrest.”* \| \| S   - **Trust in doctors as primary messengers:**   *“Doctors… especially general physicians, pediatricians, and GPs… can be involved through educational seminars.”* \| - **Decision-making still rests with parents, not adolescents:**   *“Decision is up to the parents… not the girl.”*   - **Cancer and reproductive health are taboo topics:**   *“In our country, reproductive health is still considered taboo… what will they tell their family members, their father, brother?”*   - **Fear of side effects influenced by past vaccine campaigns:**   *“People said polio drops are to reduce birth rates… that it will make girls infertile.”* \| \| M   - **Role modelling: vaccinated her children and advocated publicly:**   *“I shared my vaccination story on social media… to reduce people’s fears.”* \| - **Worried that linking HPV vaccine to STDs may reduce acceptance:**   *“If we talk about STD, then I think it can have a negative impact.”*   - **Fear of backlash if rollout happens without prior education:**   *“There should be a campaign… from health department… to avoid trust issues.”* \| \| P   - **Trust in EPI system and government vaccines:**   *“As a pediatrician, I prefer government vaccines… I trust their cold chain and source.”*   - **No access issues reported; positive outreach mechanisms:**   *“There is a good setup of EPI… and outreach is not an issue.”*   - **Recommends engaging nurses and students for counseling:**   *“We should make nurses and medical students part of our workforce… it’s good community service.”* \| - **Shortages of MMR reflecting supply risk:**   *“MMR is not available… mumps vaccine is already short in Pakistan.”*   - **Concerns that new vaccines may face similar availability issues:**   *“Because it is new and expensive… such problems can arise.”* \| \| **6** \| T   - **Believes in HPV vaccine uptake if awareness created:**   *“If we explain to them the side effects, the disadvantages and the benefits, they will understand us.”* \| - **Disinterest from families who are not directly affected:**   *“They say… our children are healthy, so what’s the point of medicines?”* \| \| S   - **Suggests school settings and PTMs for outreach:**   *“If they are approached in schools… and if there is a parent-teacher meeting… they can be counseled.”*   - **Family physicians seen as important touchpoints:**   *“The doctor should counsel nicely… then the patient gets satisfied.”* \| - **Gender and age-related shyness among adolescent girls and communities:**   *“Girls of this age… they are shy… they don’t talk openly… they don’t let us touch them.”*  *“They do not discuss such things with anyone. They do not trust anyone or share their issues.”*   - **Cultural resistance, especially from different ethnic background:**   *“Especially the people from Pathan… they do not think it is good to get vaccinated.”*   - **Distrust from community members despite professional engagement:**   *“We used to give pamphlets, but people looked at us as if we were asking for something.”* \| \| M   - **Personally willing to receive HPV vaccine:**   *“Yes, I will go for it.”*   - **Experienced in community education and motivated to counsel:**   *“We used to guide people… breast cancer is very common… we used to give them pamphlets.”*   - **Positive counseling seen as a critical motivator:**   *“If we counsel properly… if the person listens… then maybe they will understand.”* \| - **Youth indifferent to preventive health without illness:**   *“Young patients… they were not getting vaccinated(COVID)… said ‘we are not immunocompromised.’”* \| \| P   - **Belief in structured campaigns for awareness:**   *“If there is a gathering… then we can tell them our opinions.”* \| - **High patient load in public hospitals limits counseling:**   *“There is a lot of workload… even in OPD and ER, we don’t take full history.”*   - **No standardized HPV screening or tracking mechanism:**   *“I have seen 2 cases… but no one is doing screening… no data is collected.”*  *“There is no screening in the government hospitals… patients are just discharged with symptomatic treatment.”* \| \| **7** \| T   - **Strong endorsement of preventive health measure through HPV vaccine:**   *“It is such a good preventive measure, it should be told.”*   - **Observes increasing cervical cancer cases in younger women:**   *“Very young ages are being affected… 30s even… otherwise, most cases were 60s and 50s.”* \| - **Low compliance with follow-up despite counseling:**   *“If patient feels better after initial treatment, she doesn’t come for follow-up.”* \| \| S   - **Believes in dual counseling of both parents:**   *“Males absorb things more… their perception is better… both parents should be counselled.”*   - **Colleagues with daughters were convinced when informed:**   *“I told them to get them vaccinated, and they were convinced.”*   - **Trust in gynecologists and oncologists for public messaging:**   *“Gynecologists and oncologists should address people on social media… it will have more effect.”* \| - **Cultural myths around fertility and new vaccines:**   *“People start making myths… fertility issues will happen...”*   - **Taboo around discussing sexual transmission in younger girls:**   *“You can’t tell them(adolescent girls) it’s sexually transmitted… they are immature… but obviously, their parents can be counselled.”* \| \| M   - **Motivated to recommend vaccine when available:**   *“When COVID came, people refused initially… but eventually 60% got vaccinated… it will be the same for this(HPV vaccine).”*   - **Believes partial uptake is still success:**   *“Even if 60% or 50% people get vaccinated… this is also a very big number for us.”* \| - **Mother’s limited travel autonomy limits opportunities to educate the patients:**   *“They(females) say they couldn’t come as their husband didn’t come, children are small, who should we come with?”* \| \| P   - **No supply issue is anticipated if government backs the rollout:**   *“When the government takes a step, then everything is fine.”*   - **Screening and vaccination ideally integrated:**   *“If we emphasize cervical screening… we can save ourselves from a lot.”* \| - **Cytology is unaffordable for many patients:**   *“For cytology, they have to pay 2,000–3,000… they don’t even come for free screening.”*   - **Counseling impact limited if there is loss to follow up:**   *“2 out of 10 patients will come, 8 will not come.”*   - **Awareness of HPV screening and vaccination is lacking:**   *“There is no awareness of screening… vaccination is a lot more advanced step.”* \| \| **8** \| - **Would vaccinate her own daughter and relatives:**   *“I for my daughter and siblings… I think I would opt for it.”* \| - **Gender-specific concerns may complicate rollout:**   *“They’ll say… you’re already finishing its germs… that’s how it’s seen.”* \| \| S   - **Fathers may be more receptive than mothers:**   *“I think fathers would be more receptive as compared to mothers.”*   - **Believes mothers can convince families if they trust:**   *“If they are counselled properly… then they will convince their husbands.”* \| - **Use of the word "cancer" causes panic:**   *“We use ‘jaron wali rasoli’ instead of ‘cancer’… the patient gets scared.”*   - **Women’s health often deprioritized in family roles:**   *“They are least concerned about themselves… they keep themselves last.”*   - **Infertility fear is dominant myth:**   *“People will think this is a medicine to end the germ cells… related to infertility.”* \| \| M   - **Sees cancer experience as a strong motivator:**   *“I have seen such complicated cases… so I think we would opt for it.”*   - **Supports incentives for community engagement:**   *“They send girls to school when NGOs give cooking oil… same model can work here.”* \| - **Hard to convince patients:**   *“…convincing them is very difficult.”*   - **Perceived low self-value in women limits motivation:**   *“She is not concerned about herself… says I have to take care of my children.”* \| \| P   - **Advocates mass media and patient testimony:**   *“Bring the cancer patients in front… this will be more effective.”*   - **Supports tailored, visual IEC material:**   *“Visually engaging pamphlets in Urdu… with global data to show vaccine is effective.”*   - **Recommends targeted staff training:**   *“Health workers should be counselled from baseline in the periphery.”* \| - **Lack of Pap smear availability in public hospitals:**   *“Pap smear is not available with us in government setup… have to get it done privately.”*   - **Cost barriers to screening discourage preventive action:**   *“It costs around 5,000 to 6,000 rupees… most patients refuse when they hear the price.”* \| \| **9** \| T   - **Recognizes rising burden of cervical cancer:**   *“In the past 6 months, we have seen 6 cases… Now it is very common.”*   - **Understands benefit of early HPV vaccination:**   *“It was not available in our time… so we would love to vaccinate our daughters.”*   - **Believes awareness can overcome hesitancy:**   *“With time… people are getting polio drops, people got COVID vaccination later as well… education and awareness is important.”* \| - **Difficulty counseling about unmarried girls:**   *“It becomes difficult to convince them for unmarried… and I don't think it is so easily available.”*   - **Reluctance to share sexual history even in clinical settings due to lack of privacy and increased patient load:**   *“We cannot give that much time that they build a rapport and disclose to us openly.”*   - **Past vaccine myths will resurface with HPV:**   *“Like it was with COVID vaccine… people will associate it with infertility.”* \| \| S   - **Acknowledges male concern for daughters’ health:**   *“For daughters, they(fathers) will be very concerned to protect them.”*   - **Doctors can build trust over time:**   *“When rapport develops… patients start giving weightage to what you (doctors) say.”*   - **Acknowledges the role of social mobilizers and health workers:**   *“Community workers… understand their taboos… can guide them in a good way.”* \| - **Male and elderly females (e.g., mother-in-law) make key decisions:**   *“Women do not make decisions… if their husband is telling them… they are suppressed.”*   - **Taboos often rooted in elder female beliefs:**   *“Actually these are women who spread this misinformation… his mother or grandmother gave him this information.”*   - **Counseling men difficult due to the limitation of their entry in OPDs:**   *“It is not our tradition that male partners come in our gynecological OPDs.”* \| \| M   - **Committed to offer HPV counseling despite clinical workload:**   *“We offer it, we give awareness, we plan that our daughters should be vaccinated.”*   - **Advocates patient empowerment through repeated visits:**   *“If one person says, let’s think about it… then half the problem is solved.”* \| - **Counseling hampered by time constraints:**   *“Unfortunately, we do not have time… human resource is limited.”*   - **Hesitation to begin discussion on reproductive health issues and safe sexual practices:**   *“We can say it in limited, covered words… like ‘males are not trusted.’”* \| \| P   - **Advocates school-based vaccination:**   *“It is practically possible… and a better opportunity, rather than making a new setup.”*   - **Supports media, mobile outreach, and school programs:**   *“If cervical awareness is shown… girls themselves would like to be vaccinated.”* \| - **HPV screening expensive and largely unavailable:**   *“HPV DNA testing is around 8000 rupees… available only in private labs.”*   - **No national registry or regular Pap smear protocol:**   *“Recommended every 3 years, but not followed in Pakistan.”*   - **Limited presence of dedicated STD clinics for men:**   *“Separate, dedicated STD clinics should be there… husbands don’t come forward.”* \| \| **10** \| T   - **Belief in effectiveness of vaccines from personal experience:**   *“My daughter had chickenpox after getting vaccinated, but the severity was reduced—only a few pustules appeared.”*   - **Trust in government and WHO-backed efforts:**   *“If the government is making this effort for their(public) benefit, then they should cooperate and participate.”* \| - **Concern about side effects, especially for children:**   *“Naturally, when receiving a vaccine for the first time, concerns arise… parents are especially anxious about any risk to the child’s life.”*   - **Suspicion based on past rumors e.g., relating polio drops with family planning:**   *“They will say these drops are given so that people’s children are not born.”* \| \| S   - **Mothers seen as key decision-makers:**   *“I am related to medicine, so my husband trusts me… mothers mostly take more decisions.”*   - **Doctors highly trusted for health information:**   *“Doctors—whatever the doctor says, people do it… even we consult doctors ourselves.”*   - **Social media influencers cited as powerful sources:**   *“Dr. ABC and a female doctor from a city in Punjab—I've watched their videos on cervical cancer.”*   - **Routine immunization well accepted even among religious groups:**   *“Yes, everyone comes here… religious people too.”* \| - **Reproductive health concerns:**   *“Some people will ask why only this organ, why not breast cancer vaccine.”*  *“In eastern society, there will likely be questions about why this area is being vaccinated.”* \| \| M   - **Personally willing to vaccinate her daughter:**   *“I have a 9-year-old daughter. I will get it done.”*   - **Positive response predicted if awareness is done properly:**   *“Seminars, ads, media engagement can create awareness… and motivate people.”*   - **Financial incentives may increase uptake:**   *“If a reward is attached, people will get it. That’s how things work here.”* \| - **Fear of new vaccine rollouts repeating COVID anxiety:**   *“During COVID vaccine, there was anxiety, high BP… so similar concerns will arise.”* \| \| P   - **Positive public response if vaccine is free:**   *“If they get it free of cost, then maybe people will take it.”*   - **Experienced in counseling patients about routine side effects:**   *“We guide them about fever after Penta… no antibiotics needed, just Panadol.”* \| - **Staff shortage in EPI center affects quality time with clients:**   *“There were 3 of us, now only 1 person was hired after 2 retired… we are overworked.”*   - **Limited time for education and detailed discussion:**   *“We don’t have time to talk to them for so long… very few people ask us questions.”*   - **Rumors linked to monetary entitlements create mistrust:**   *“People think the local staff are eating up the incentive money promised in programs like Aghosh.”* \| \| **Program Managers** \| \| \| \| **1** \| T   - **Acknowledges the need for HPV vaccination and regrets its late rollout:**   *“It should have been done a long time ago. But the time has come.”* \| - **Cancer and vaccines are poorly understood by the public:**   *“They believe germs are being introduced. Their logic is—if there are no germs present, how can someone get sick?”*   - **Parents hesitate due to poor understanding of asymptomatic disease prevention:**   *“This vaccine is for healthy girls. Parents often ask why it’s needed if their child is fine.”* \| \| S   - **Supports school-based parent engagement via PTMs:**   *“PTMs are the best opportunity… Parents should be encouraged to attend and be informed.”*   - **Trust in doctors and existing vertical programs:**   *“People trust doctors the most when it comes to counseling.”* \| - **Social taboos affect vaccine acceptance for girls:**   *“It concerns the female side, so this is even more sensitive than polio.”*   - **Male dominance limits mothers’ role in decision-making:**   *“Male dominance is still a reality… in infertility too, the focus is on women.”*   - **Infertility is a dominant fear:**   *“Some believe vaccines cause infertility. This misconception is still widespread.”* \| \| M   - **Optimistic about success if health staff are mobilized:**   *“When our team will educate people properly, it will definitely work.”* \| - **Understands behavioral resistance and calls for pre-emptive awareness:**   *“If you skip the education step and just start handing out consent forms… it will likely lead to refusals.”* \| \| P   - **Using existing vertical program structure to implement the HPV vaccination program would help:**   *“Our team structure has now merged… better liaison and open communication improves delivery.”*   - **Referral systems for positive Pap smears exists:**   *“Pap smear slides go to city, and patients are informed.”* \| - **Health education departments are missing or disjointed:**   *“There isn’t a dedicated department for health messaging.”*   - **Community violence toward health workers:**   *“Our volunteers come back beaten… Even in city.”*  *“There is very little tolerance… even security doesn’t work with mobs.”*   - **Healthcare worker motivation affected by system burden:**   *“Doctors need 20–25 minutes per patient to answer all questions… it’s not justifiable in a busy hospital.”*   - **Lack of data demotivates advocacy:**   *“We don’t have exact data on cervical cancer. Once people are educated, only then will we see the real picture.”* \| \| **2** \| T   - **Sees HPV vaccine as a long-overdue intervention:**   *“It should have been done a long time ago. But the time has come.”*   - **Frames HPV as a cancer-prevention initiative to build trust:**   *“We will focus on the point in time that it is the only vaccine which is addressing cancer.”*   - **Plans clear messaging tailored to minimize controversy:**   *“We’ll focus less on sexual transmission and more on cancer prevention.”* \| - **Recognizes cancer and HPV are less familiar terms:**   *“Typhoid and measles are well known… HPV is new… even girls themselves will Google it.”*   - **Anticipates public discomfort around reproductive health:**   *“When you say ‘bachaydani’… people become sensitive.”* \| \| S   - **Will involve LHWs and LHVs as trusted community workers:**   *“LHWs will play the role of social mobilizers… and sometimes support with vaccination.”*   - **Plans to conduct school-based campaigns:**   *“We’ll go into schools, do sessions… media briefings, social media, print—all kinds.”* \| - **Male dominance and consent remain cultural obstacles:**   *“Male dominance is still a reality. We won’t ask for consent; we’ll just inform.”*   - **Prior consent forms led to refusals and limited access:**   *“When we ask for consent, 50% refuse, and schools stop vaccinators at the gate.”* \| \| M   - **Government highly motivated and fully funded by GAVI:**   *“This is not a pilot; it’s a 100% rollout. We’ve never had availability or financing issues.”*   - **Female staff and nurses are prioritized to build acceptance:**   *“Female vaccinators will be deployed… even nursing schools will support.”*   - **Supports tailored ACSM strategies and IPC modules:**   *“There’s a complete guideline… even interpersonal communication modules are included.”* \| - **Fear of infertility and religious myths still linger:**   *“Like with polio… people will say it is for birth control.”* \| \| P   - **Fixed-site, outreach, and mobile teams are already functional:**   *“The same EPI model will be used—fixed, outreach, and mobile strategies.”*   - **Cold chain and logistics infrastructure fully in place:**   *“We can handle 90 million doses at once… HPV is just 11 million.”*   - **NEIR (National Electronic Immunization Registry) used for daily updates:**   *“Supervisors will upload data electronically each evening. Paper-based at team level.”* \| - **School-based rollout differs from polio and poses limitations:**   *“This is not like polio where you knock and vaccinate. Here, they must come to us.”* \| \| **FGD 1** \| \| \| \|  \| T   - **Belief in vaccine effectiveness:**   *"We explain to parents that vaccines are beneficial for children."*   - **Trust in government-supplied vaccines vs private sector:**   *"We tell them to compare—get the vaccine from our center, then check the price in the market."*   - **Growing health awareness in the digital age:**   *"Nowadays people are much more informed. They search everything on Google."* \| - **Lack of knowledge about cervical cancer:**   *"I personally am hearing about cervical cancer for the first time."*   - **Side effect concerns from past experiences (e.g., COVID):**   *"They say this injection causes high fever, and medicine after the vaccine costs money."*   - **Fear of infertility and hidden agenda:**   *"People ask if this is a Jewish vaccine or for family planning."*   - **Parents skeptical of free vaccine value:**   *"People find it hard to believe the government gives expensive vaccines for free."* \| \| S   - **Strong community trust in LHWs:**   *"People trust us a lot... their husbands say, 'ask the LHW, only then get vaccinated.'"*   - **Peer influence in vaccine acceptance:**   *"We tell them, families on this street got it—nothing happened to their kids."*   - **Use of Sehat committees for male engagement:**   *"We also have separate sessions for men in our Sehat committee meetings."* \| - **Influence of religious narratives and male dominance:**   *"Pathan families strictly refuse… They say it’s a Jewish plot."*   - **Resistance from school authorities:**   *"Private schools say parents are refusing... we need ASVs to intervene."*   - **Teachers not trained for awareness:**   *"No training was given to teachers in the COVID campaign."* \| \| M   - **Willingness to vaccinate own children if given proper knowledge:**   *"If we are confident in the vaccine, we’ll vaccinate our own daughters."*   - **Policy-linked motivation (e.g., school entry requirements):**   *"Make it like COVID—no vaccine, no admission or exam."*   - **Professional availability:**   *"Our doors are open 24/7. We are available even at night."* \| - **Suspicion due to lack of disease visibility:**   *"If there’s no known patient around, parents ask why the vaccine is needed."*   - **Hesitation without community exposure to the vaccine:**   *"If we had a real-life example—vaccinated girl who married and had children—it would help."* \| \| P   - **Women-centered support group model:**   *"Each LHW has 4 support groups of 15-20 women from her area."*   - **Integrated community presence and data tracking:**   *"We maintain detailed diaries of each child’s birthdate and vaccine schedule."*   - **Mobile vaccination models exist (BCU, key points):**   *"We set up vaccine centers in homes... inform people door to door."*   - **Observational follow-up:**   *"We monitor children after injection before letting them go."* \| - **One male vaccinator per UC limits outreach:**   *"Sometimes parents object to male vaccinators... prefer female."*   - **Training gaps for new vaccines:**   *"We need clear and proper training before rollout."*   - **Record keeping still manual:**   *"No computerized system—everything is in the LHW diary."*   - **Brand skepticism:**   *"People asked which vaccine is better, Pfizer or Chinese."* \|   Based on the in-depth interview transcript with the Lady Health Worker (LHW) , I have coded the data using the **WHO Behavioural and Social Drivers (BeSD) Framework**. The framework includes four domains:   1. **Thinking and Feeling** 2. **Social Processes** 3. **Motivation (Intent)** 4. **Practical Issues**   Below is the thematic framework organized accordingly, including **codes**, **sub-themes**, and **themes**:  **1. Thinking and Feeling**   \| **Codes** \| **Sub-Themes** \| **Themes** \| \| --- \| --- \| --- \| \| Fear of side effects (swelling, fever) \| Vaccine-related fears \| Perceived health risks and concerns \| \| Fear of infertility \| Misconceptions about vaccine impact \| Myths and misinformation \| \| Cancer is life-threatening, so people are afraid \| Emotional response to disease threat \| Fear-driven hesitation \| \| “We explain it's for your benefit” \| Health literacy through counselling \| Perceived benefits of vaccination \| \| Comparing costs of treatment vs. prevention \| Understanding health economics \| Risk-benefit awareness \| \| Reference to COVID vaccine hesitancy due to misinformation \| Impact of previous vaccine experiences \| Legacy of vaccine distrust \| \| “If my child is fine, yours will be too” \| Peer modeling \| Reassurance through community examples \|   **2. Social Processes**   \| **Codes** \| **Sub-Themes** \| **Themes** \| \| --- \| --- \| --- \| \| Trust in LHWs (“they consider us nani-dadi”) \| Community rapport \| Trust in frontline health workers \| \| Use of local references and community figures \| Social proof and identity \| Influence of local leadership \| \| Female modesty/shyness in schools \| Gender sensitivity \| Gendered social norms \| \| People trust vaccinated examples in community \| Social modeling \| Collective behavior and peer influence \| \| “We become mother-in-law or grandmother to counsel them” \| Cultural role flexibility \| Relational identity of LHWs \| \| Word of mouth (use of LHW name by others in community) \| Reputation and endorsement \| Social credibility \|   **3. Motivation (Intent)**   \| **Codes** \| **Sub-Themes** \| **Themes** \| \| --- \| --- \| --- \| \| “We will get it done first” (referring to HPV) \| Proactive endorsement \| Intrinsic motivation among health workers \| \| Role modeling by vaccinating their own children first \| Leading by example \| Positive personal intent \| \| Awareness about cancer motivates intent \| Disease knowledge fosters motivation \| Health-protective intention \| \| “If we don’t vaccinate, child will suffer” \| Preventive mindset \| Future-oriented motivation \| \| Expectation of monetary reward from vaccination (reference to Sindh) \| Incentive-based motivation \| Conditional intention \| \| “Trust us today, or regret tomorrow” \| Emotional leverage to motivate \| Moral-ethical persuasion \|   **4. Practical Issues**   \| **Codes** \| **Sub-Themes** \| **Themes** \| \| --- \| --- \| --- \| \| Excuses for non-vaccination (child sick, forgot, no one to take him) \| Daily life barriers \| Household-level logistical constraints \| \| Shortage of vaccines (e.g., pneumococcal, meningitis) \| Supply chain gaps \| System-level limitations \| \| Fixed catchment areas (2,000 population policy) \| Staffing structure \| Coverage gaps due to workforce policies \| \| Need for door-to-door service \| Access facilitation \| Home-based outreach critical for uptake \| \| Use of schools for vaccination \| Institutional access points \| School-based delivery systems \| \| Sessions with posters, brochures, media awareness \| Health communication tools \| Multichannel outreach strategies \| \| Low LHW coverage in some UCs \| Workforce shortage \| Structural inequity in access \| \| Training issues (need for refreshers, counselling skills) \| Capacity building gaps \| Training and preparedness of health staff \|   **BeSD TOOL Annotated with Codes, Sub-Themes, and Themes**  **1. Thinking and Feeling Domain**  **Existing Questions:**   - How important do you think HPV vaccines are for your child’s health? - How safe do you think HPV vaccines are for your child?   **Suggested Additions / Probes:**   - Have you heard concerns that HPV vaccine might cause infertility or other health problems? *(Code: fear of infertility, sub-theme: misconceptions about vaccine impact)* - Are you afraid of HPV because it is a type of cancer? *(Code: fear of cancer, sub-theme: emotional response to disease threat)* - What concerns do you have, if any, about this vaccine causing side effects like fever or swelling? *(Code: fear of side effects, sub-theme: vaccine-related fears)*   **2. Social Processes Domain**  **Existing Questions:**   - Do you think most parents you know get their children vaccinated? - Do you think your close family/friends want you to get your child HPV vaccinated? - Do you think your religious/community leaders support HPV vaccination? - Has a health worker recommended your child be HPV vaccinated?   **Suggested Additions / Probes:**   - Do you trust the local health workers such as Lady Health Workers in your area? *(Code: trust in LHWs, sub-theme: community rapport)* - Have you seen examples of other children in your community being vaccinated without harm? *(Code: social modeling, sub-theme: peer influence)* - Would you be more likely to vaccinate if a respected community member or local health worker showed their own child getting vaccinated? *(Code: role modeling, sub-theme: trust and influence)*   **3. Motivation (Intent) Domain**  **Existing Questions:**   - Do you want your child to get this vaccine? - When this vaccine is launched, do you want to be contacted?   **Suggested Additions / Probes:**   - If you had the opportunity, would you volunteer to vaccinate your child first to set an example? *(Code: proactive endorsement, sub-theme: intrinsic motivation)* - Do you think being vaccinated could prevent expensive treatment costs in the future? *(Code: understanding health economics, sub-theme: preventive mindset)* - If others in your community vaccinated their children, would you feel more comfortable doing the same? *(Code: peer modeling, sub-theme: moral-ethical persuasion)*   **4. Practical Issues Domain**  **Existing Questions:**   - Do you know where to go to get your child HPV vaccinated? - Have you ever been turned away? - How easy is it to get services/pay for vaccination? - What makes it hard? - How satisfied are you with the services?   **Suggested Additions / Probes:**   - Have you ever delayed vaccination because your child was sick or you had no one to take them? *(Code: excuses for non-vaccination, sub-theme: household constraints)* - Have you ever found the clinic closed or vaccine unavailable when you went? *(Code: supply chain gaps, sub-theme: system-level limitations)* - Are there Lady Health Workers visiting your area to provide vaccines? *(Code: low LHW coverage, sub-theme: workforce shortages)* - Would it help if awareness sessions or health camps were conducted in your community? *(Code: multichannel outreach, sub-theme: access facilitation)* - Do you think training and attitude of vaccinators affect your trust in the service? *(Code: poorly trained staff, sub-theme: staff credibility and service quality)*   **Summary Table of Key Themes Mapped to BeSD Tool Sections**   \| **BeSD Domain** \| **Mapped From Interview** \| **Modified or Suggested Question Areas** \| \| --- \| --- \| --- \| \| Thinking & Feeling \| Fear of side effects, infertility, cancer, cost awareness \| Perceived safety, perceived risk, perceived benefit \| \| Social Processes \| Trust in LHWs, peer influence, cultural roles \| Trust questions, role modeling, community endorsement \| \| Motivation (Intent) \| Personal intent, future regret, social proof \| Role modeling, conditional intent, influence of examples \| \| Practical Issues \| Workforce shortages, access gaps, service satisfaction \| Service availability, supply gaps, outreach methods \| |  |
