# Supplementary material for: From global to local: Developing a context-specific BeSD-HPV tool through cultural and linguistic adaptation in Pakistan
Source: PLoS One. 2026 Jun 15;21(6):e0350162. doi: 10.1371/journal.pone.0350162 (PMC13268181; doi:10.1371/journal.pone.0350162)
Supplement: S3 File — (DOCX) [file pone.0350162.s003.docx]

BeSD Framework adaptation_12_June_25

Codes

Codes\\HPV Vaccine

| Name | Description | Files | References |
| --- | --- | --- | --- |
| Confidence in vaccine safety |  | 0 | 0 |
| Fear of side effects |  | 6 | 8 |
| Consultation with adolescent girls |  | 5 | 5 |
| Knowledge about HPV vaccine |  | 3 | 3 |
| Knowledge of cervical cancer |  | 10 | 10 |
| Motivation |  | 0 | 0 |
| Fear-based motivation |  | 6 | 8 |
| Positive reinforcement |  | 5 | 6 |
| Need for HPV vaccine |  | 2 | 2 |
| Negative opinions |  | 1 | 1 |
| Positive opinions |  | 3 | 7 |
| Opinion about the cost of the vaccine |  | 2 | 3 |
| People's perception of HPV vaccine |  | 1 | 3 |
| Negative perceptions |  | 3 | 5 |
| Positive perceptions |  | 1 | 1 |
| Role of Husbands, family |  | 7 | 11 |
| Vaccine hesitancy |  | 4 | 4 |
| Whome will the people seek consultation from |  | 6 | 12 |

Codes\\Motivation

Readiness to be vaccinated Personal decision-making power (especially among adolescents) Cultural or gender-related attitudes

| Name | Description | Files | References |
| --- | --- | --- | --- |
| Extrinsic motivation |  | 0 | 0 |
| Experience of others |  | 1 | 1 |
| Government mandate |  | 1 | 1 |
| Positive reinforcements |  | 1 | 1 |
| Religious beliefs |  | 0 | 0 |
| role of social media |  | 1 | 3 |
| Intrinsic motivation |  | 7 | 13 |
| Fear based motivation |  | 4 | 6 |
| Uptake of childhood vaccination |  | 4 | 7 |

Codes\\Practical issues

Availability of the vaccine Accessibility of vaccination sites Cost, travel, school-based vs. clinic-based programs Reminder systems and convenience

| Name | Description | Files | References |
| --- | --- | --- | --- |
| Access to vaccination facility |  | 2 | 3 |
| Consent taking |  | 3 | 4 |
| Negative experience with healthcare delivery |  | 0 | 0 |
| Outreach services |  | 3 | 7 |
| Public vs private facility |  | 4 | 4 |
| Vaccine availability |  | 3 | 5 |
| Vaccine facility satisfaction |  | 6 | 9 |

Codes\\Recommendations

| Name | Description | Files | References |
| --- | --- | --- | --- |
| By Caregivers |  | 9 | 31 |
| Access to vaccine |  | 1 | 1 |
| Approach through the educational institutions |  | 6 | 9 |
| Demand for awareness |  | 8 | 11 |
| Evidence based sensitization |  | 3 | 4 |
| Need for Government initiative |  | 1 | 2 |
| Role of social media |  | 3 | 3 |

Codes\\Social processes

Influence of parents, peers, teachers, and religious/community leaders Social norms and acceptability of HPV vaccination Stigma related to sexual health (relevant for HPV)

| Name | Description | Files | References |
| --- | --- | --- | --- |
| Decision process |  | 9 | 21 |
| Experiencing health loss |  | 1 | 1 |
| Family norms |  | 9 | 19 |
| Health worker counselling |  | 0 | 0 |
| Mothers travel autonomy |  | 1 | 1 |
| Public health Advocacy |  | 0 | 0 |
| Social norms |  | 10 | 45 |
| Anti-government stance | Thoughts about cost of vaccines | 2 | 4 |
| thoughts about the cost of vaccination |  | 1 | 2 |
| Community leader norms |  | 1 | 1 |
| Peer norms |  | 8 | 14 |
| Religious leader norms |  | 3 | 8 |
| Trust in Government initiatives |  | 3 | 4 |
| Trust in Healthcare providers |  | 2 | 3 |
| Trust in school teachers |  | 3 | 6 |
| Vaccine refusal |  | 5 | 5 |

Codes\\Thinking and Feeling.

Trust in the vaccine and health system Perceived risk of HPV and cervical cancer Concerns about side effects General vaccine confidence or hesitancy

| Name | Description | Files | References |
| --- | --- | --- | --- |
| Fear of side effects |  | 4 | 4 |
| Percieved benefits of vaccines |  | 7 | 13 |
| Percieved skepticism towards vaccines | Negative views | 2 | 4 |
| Religious perceptions |  | 1 | 1 |
| Speculation over vaccine components |  | 2 | 2 |
