# Supplementary material for: From global to local: Developing a context-specific BeSD-HPV tool through cultural and linguistic adaptation in Pakistan
Source: PLoS One. 2026 Jun 15;21(6):e0350162. doi: 10.1371/journal.pone.0350162 (PMC13268181; doi:10.1371/journal.pone.0350162)
Supplement: S4 File — (DOCX) [file pone.0350162.s004.docx]

**Information sheet for reference**

| **Domain** | **Statements** | **Question number** |
| --- | --- | --- |
| **Thinking and Feeling** | **How safe do you think HPV vaccine is for your daughter?** | **1** |
|  | **How much do you trust the health workers who give HPV vaccines?** | **2** |
|  | To what extent do you agree that preventive measures, such as vaccination, are necessary to protect women from serious illnesses? | **3** |
|  | In your opinion, could the vaccines being administered in Pakistan be used for experimental purposes? | **4** |
|  | In your opinion, do vaccines contain ingredients that are prohibited in religion (pork or alcohol)? | **5** |
|  | To what extent do you think that the HPV vaccine beneficial to the women in Pakistan? | **6** |
|  | To what extent do you believe that the HPV vaccine protects against cervical cancer, based on your existing knowledge? | **7** |
|  | Do you believe is your current knowledge sufficient to inform you regarding vaccinating against HPV? | **8** |
|  | How much do you trust the HPV vaccine being introduced in your country? | **9** |
|  | Do you think that if the government makes the HPV vaccine mandatory, some people will try to get a card without actually being vaccinated due to their biases? | **10** |
|  | If the HPV vaccine were offered free of cost, do you think people would receive it? | **11** |
|  | Do you believe the HPV vaccine is acceptable in a conservative society? | **12** |
|  | To what extent do you agree that all girls aged 9 to 14 years should be vaccinated against HPV? | **13** |
|  | If you think the women in your family are at low risk of cervical cancer due to family history, would you still vaccinate them against HPV? | **14** |
|  | Do you think the HPV vaccine will help protect women in your family from serious illness? | **15** |
|  | To what extent do you think a person’s strong natural immunity affects their decision to receive the HPV vaccine? | **16** |
|  | To what extent are you concerned about the possible adverse effects of the HPV vaccine? | **17** |
|  | To what extent do you think the HPV vaccine could negatively affect reproductive health? | **18** |
|  | How much do you trust social media to get information about the HPV vaccine? | **19** |
| **Social Processes** | **Do you think most parents you know would want you to get your daughter vaccinated against HPV ?** | **20** |
|  | **Do you think most of your close family and friends want you to get your daughter vaccinated against HPV?** | **21** |
|  | **Has a health worker recommended your daughter vaccinated against HPV?** | **22** |
|  | **If it was time for your daughter to get vaccinated, would the mother need permission to take your child to the clinic?** | **23** |
|  | How often do you hear rumors or misconceptions about the HPV vaccine in your community? | **24** |
|  | To what extent do religious beliefs in your community influence decisions about the HPV vaccine? | **25** |
|  | .To what extent do people in your community trust information about the HPV vaccine shared on social media platforms? | **26** |
|  | Would you prefer to wait for efficacy of HPV vaccine being proven before deciding on the HPV vaccination? | **27** |
|  | If a community leader recommends the HPV vaccine, would you be more willing to get the girls in your family vaccinated? | **28** |
|  | Would your family involve the girls in discussions about HPV vaccination before deciding on vaccination? | **29** |
|  | Would you recommend the HPV vaccination to others? | **30** |
|  | Would you prefer that the healthcare provider educating about the HPV vaccine be a woman? | **31** |
|  | To what extent do you trust the following sources for accurate information about the HPV vaccine? | **32**  **(5 parts)** |
| **Motivation** | **Do you want your daughter to get vaccinated against HPV ?** | **33** |
|  | When the HPV vaccine campaign begins, do you intend to register the girls in your family? | **34** |
|  | To what extent do you believe vaccinating girls against HPV is as important as receiving other childhood vaccines? | **35** |
|  | If you know that cervical cancer cases are increasing among young women in your area, will it motivate you to get the girls in your family vaccinated? | **36** |
|  | Would your past experience with routine immunization impact your decision about HPV vaccination? | **37** |
|  | Compared to other vaccines, how much do you trust the safety of the HPV vaccine? | **38** |
|  | If you are given scientific information about the HPV vaccine, would you consider getting the girls in your family vaccinated? | **39** |
|  | To what extent would learning that other countries are administering the HPV vaccine increase your trust in receiving the vaccine? | **40** |
| **Practical Issues** | **Have you ever been contacted about your daughter being due for HPV vaccination?** | **41** |
|  | Would you get the girls in your family vaccinated if there is sufficient data available on the impact of usage of the HPV vaccine in Pakistan? | **42** |
|  | If healthcare facility staff provide sufficient information about the HPV vaccine, would it increase your confidence in choosing HPV vaccination? | **43** |
|  | Do you think incentives such as financial rewards, free healthcare services, or educational benefits should be used to promote HPV vaccination? | **44** |
|  | If HPV vaccination is made mandatory by the government, do you think schools are the appropriate places to vaccinate the girls? | **45** |
|  | If HPV vaccination becomes a legal requirement (e.g., for school admission or travel), would you ensure that the girls in your family receive the vaccine? | **46** |
|  | Do you agree that public and private schools have different attitudes towards vaccination campaigns? | **47** |
|  | To what extent do you think teachers should receive specific training to raise awareness about the HPV vaccine among adolescent girls? | **48** |
|  | Do you think girls should be given awareness about the HPV vaccine in schools to support successful launch? | **49** |
|  | Do you trust outreach vaccination services for the HPV vaccination? | **50** |
|  | In your view, how much does it matter to people if the HPV vaccine is produced locally versus being imported? | **51** |
|  | Based on your past experience, to what extent do you think vaccination staff are cooperative? | **52** |
|  | To what extent would you prefer each of the following places as the site for receiving the HPV vaccination? (School, Fixed site, outreach services) | **53**  **(3 parts)** |
|  | To what extent do you think each of the following barriers exists in your community regarding access to vaccines? | **54**  **(7 parts)** |
|  | To what extent do you usually experience long waiting times at the health center for routine vaccination? | **55** |
|  | To what extent do you trust each of the following social media platforms for vaccine-related information? Platforms to be rated separately: | **56**  **(6 parts)** |
|  | To what extent do you think reminders (e.g., mobile messages, school announcements) are helpful in ensuring timely vaccination? | **57** |
|  | To what extent do you receive vaccine-related information through TV, radio, or mobile phone? | **58** |
| **Cultural Integration** | To what extent does each of the following usually make health decisions in your household? (Maternal grandparents, Paternal grandparents, Mother, Father, others) | **59**  **(5 parts)** |
|  | Do you feel comfortable discussing women’s health matters with your family? | **60** |
|  | To what extent do you think men should be educated about the HPV vaccine through awareness campaigns? | **61** |
|  | To what extent would family opinions influence your decision about getting the girls in your family vaccinated? | **62** |
|  | If your daughter expresses her interest in receiving the HPV vaccine but you still have some concerns, how would you support this decision as a parent? | **63** |
|  | To what extent do you think that hearing real stories of cervical cancer patients would increase parents’/caregivers’ trust in the HPV vaccine? | **64** |
|  | If you see health care professionals vaccinating their own daughters against HPV, would you feel more confident about doing the same? | **65** |
|  | In your opinion, does the gender of the health worker affect acceptance of the HPV vaccine for women /girls ? | **66** |
|  | Do people in community hesitate to get the HPV vaccine due to social reasons? | **67** |
|  | To what extent, do you think the gender focus of HPV vaccination programs affects how people view the vaccine? | **68** |
|  | Do you think cultural background influences families’ willingness to vaccinate their daughters against HPV? | **69** |
|  | How important is parental consent when offering the HPV vaccine to school-going girls? | **70** |

**(Parts of questions are in separate columns)**

**Likert scale options (Labels and Codes)**

| **Labels** | **Codes** |
| --- | --- |
| Not at all | 1 |
| To some extent | 2 |
| Neutral (at some places there is spelling mistake as neural) | 3 |
| To a considerable extent | 4 |
| To a great extent | 5 |
| Don't know/ prefer not to say | 6 |
