# Supplementary material for: From global to local: Developing a context-specific BeSD-HPV tool through cultural and linguistic adaptation in Pakistan
Source: PLoS One. 2026 Jun 15;21(6):e0350162. doi: 10.1371/journal.pone.0350162 (PMC13268181; doi:10.1371/journal.pone.0350162)
Supplement: S1 Table — (DOCX) [file pone.0350162.s005.docx]

| **BeSD (TPB Domain) : Thinking and Feeling(Attitudes)** | | | | |
| --- | --- | --- | --- | --- |
| **Construct** | **Survey item** | **Verbatim** | **Rationale** | **Urdu question** |
| Perceived trustworthiness of vaccines | 1.Do you think that people will be hesitant about HPV vaccine if it is offered free of cost? | I have heard people say that vaccination is free. When we take medicine even for a minor disease, we have to pay a fee, but why is vaccination free everywhere. (P5) | This item assesses the belief that free vaccines are viewed with suspicion. It highlights a concern that associating a health service with no cost may indicate hidden motives or reduced quality. | کیا آپ سمجھتے ہیں کہ اگر ویکسینHPV مفت دی جائے تو لوگ اسے لگوانے میں ہچکچاہٹ محسوس کریں گے ؟ |
| Perceived trustworthiness of vaccines | 2.Do you agree with the belief that HPV vaccine offered in Pakistan may be used for experimental purposes? | People questions the hidden agenda behind free vaccination program. As a result, many initially refused vaccination. (HCW 3) | This item explores fears about the vaccine being part of experimentation on local populations. It addresses concerns about mistrust in foreign-produced vaccines, which may inhibit uptake. | کیا آپ اس بات سے متفق ہیں کہ پاکستان میں دی جانے والی ویکسین HPV کو تجرباتی مقاصد کے لیے استعمال کیا جا سکتا ہے ؟ |
| Perceived trustworthiness of vaccines | 3.Do you believe that HPV vaccine might be part of efforts to control the population? | They will say, you are giving this vaccine for 9 to 16 years, and before marriage. This is even worse… they mean to control birth rate. (P5)  Many people believe the vaccine is part of a family planning agenda and even claim it's a plot by Jews. (FGD1) | This item captures distrust linked particularly to the association between vaccination and population control, highlighting deeper religious narratives that may drive hesitancy. | کیا آپ سمجھتے ہیں کہ  ویکسین HPV  آبادی پر قابو پانے کی کوششوں کا حصہ ہو سکتی ہے ؟ |
| Perceived need for vaccination | 4.Would you choose HPV vaccine for your daughter if you believe she has a low risk of developing cervical cancer? | Young patients were not getting vaccinated for covid, they were saying, when we aren’t immunocompromised, if something happens, we will see. (HCW 6)  I’ve heard people say that giving vaccines to children increases the risk of cancer, as they believe germs are being introduced into the body. Their logic is—if there are no germs present, how can someone get sick? (PM1)  They’ll ask why we’re vaccinating someone who hasn’t been tested, who shows no symptoms or signs of illness. This could lead to a lot of confusion and resistance. (FGD1) | This item assesses how individual risk perception influences vaccine uptake. It helps understand how low perceived vulnerability may lead to reduced motivation to vaccinate. | اگر آپ سمجھتے ہوں کہ آپ کی بیٹی کو سروائیکل کینسر ہونے کا خطرہ کم ہے تو کیا آپ اپنی بیٹی کو  ویکسین HPV لگوائیں گے ؟ |
| Perceived benefits of vaccines | 5.Do you think that giving the HPV vaccine to your daughter will help prevent serious illness? | In my personal experience, I got my daughter vaccinated for chickenpox. When my daughter had chickenpox later, I think, 8 to 10 or 20 pustules appeared. I mean, The severity of the disease decreases. (HCW 10) | This item helps assess whether belief in the vaccine’s protective value influences willingness to vaccinate. | کیا آپ سمجھتے ہیں کہ  ویکسین HPV  آپ کی بیٹی کو سنگین بیماری سے بچانے میں مدد دے سکتی ہے ؟ |
| Religious perceptions | 6.Do you think HPV vaccine is unnecessary in a Muslim society? | The vaccine is related to protection with regards to sexual activity—so it should only be given with that specific purpose in mind, not for everyone. (P4) | This item explores the role of religious beliefs in the uptake of HPV vaccination. It helps identify objections tied to sexuality that may impact vaccine acceptance. | کیا آپ سمجھتے ہیں کہ  ویکسین HPV  ایک مسلم معاشرے میں غیر ضروری ہے ؟ |
| Trust in information sources – social media | 7.How much do you trust social media when it comes to gaining information regarding HPV vaccination? | …. it is the era of the media. They have heard about it from the media. Then they come and confirm with us too. (FGD1) | This item measures trust in social media as an information source. It helps in understanding the extent to which online platforms shape vaccine uptake especially in the absence of formal health communication. | ویکسین HPV کے بارے میں معلومات حاصل کرنے کے لیے آپ سوشل میڈیا پر کتنا بھروسہ کرتے ہیں ؟ |
| Trust in vaccination enforcement systems | 8.Do you think there will be attempts to bypass HPV vaccination and still obtain the card if the government makes it mandatory? | We were also responsible for making registration cards, and we’d tell people they couldn’t travel without one. This made people anxious—they wanted their entry confirmed but didn’t want to get vaccinated. Since the card was mandatory, many tried to bypass the vaccine. (FGD1) | This item addresses concerns about loopholes in vaccine programs and the practical challenges of implementing mandates. | کیا آپ سمجھتے ہیں کہ اگر حکومت  ویکسین HPV  لازمی قرار دے دے تو کچھ لوگ ویکسین لگوائے بغیر ہی کارڈ حاصل کرنے کی کوشش کریں گے ؟ |
| *Concerns about vaccine safety* | 1. How concerned are you about the potential side effects of the HPV vaccine? | People are unsure about what the side effects might be, and that uncertainty holds them back. (A2)  I’m worried about possible side effects. That’s my biggest concern. (P1) | This item assesses the degree of concern related to possible adverse effects of the HPV vaccine. It provides insight into emotional hesitancy and perceived risk, which can significantly influence decision-making. | آپ کس حد تک ویکسین HPV کے ممکنہ ضمنی اثرات کے بارے میں فکر مند ہیں ؟ |
| *Perceived necessity of vaccination* | 1. To what extent do you agree with the following statement: “All girls between ages of 9 to 14 should get the HPV vaccine.” | Because you mentioned that only girls will receive this vaccine, and I feel like women's health often isn’t given enough attention. People don’t take these things seriously unless it's a widespread issue.(A2) | This item measures beliefs about the perceived need for vaccination within the target age group. | آپ کس حد تک   اس بات سے متفق ہیں کہ ۹ سے ۱۴ سال کی تمام لڑکیوں کو HPV ویکسین لگوانی چاہئے؟ |
| *Concerns about vaccine content* | 1. Have you ever heard that vaccines contain forbidden substances (e.g., pig gelatin)? | Others express concern that vaccines may contain ingredients that aren’t halal or ethically permissible. (A1)  Some individuals believe that vaccines contained ingredients like pig gelatin or substances derived from donkey meat. These types of rumors created a lot of hesitation and mistrust among certain groups. (P7) | This item explores the influence of cultural concerns about vaccine ingredients. It reflects how rumors or misinformation about “haram” substances affect acceptance. | کیا آپ نے کبھی سنا ہے کہ ویکسین میں حرام اجزاء  جیسے کہ سور کا گوشت  شامل  ہوتے ہیں؟ |
| *Perceived alternative health beliefs* | 1. Do you think that good dietary practices reduce the need for HPV vaccine? | There hasn’t been any known case in our village or in the surrounding villages. It might be because the diets here in rural areas are generally healthier, and girls don’t face such issues as commonly. (P7) | This item captures beliefs in natural immunity that may compete with the perceived need for vaccines. It helps assess barriers rooted in health misconceptions. | کیا آپ سمجھتے ہیں کہ بہترین غذا HPV ویکسین کی اہمیت کو کم کر دیتی ہے؟ |
| *Perceived relevance of vaccine introduction* | 1. How worried are you that HPV vaccine may be introduced unnecessarily? | To be honest, no, I don’t think it is necessary. There are many other diseases out there for which we also don’t have vaccinations. In my view, not all illnesses require a vaccine. Some illnesses lead to complications over time, but not all are considered immediate threats. So, I am not fully convinced about the necessity of this particular vaccine.  (T1) | This item evaluates skepticism about the need for the HPV vaccine. It identifies concern that it may be introduced without clear justification, reflecting doubt in public health priorities. | کیا آپ سمجھتے ہیں کہ  HPV ویکسین غیر  ضروری  طور  پر مُتعارِف کی جا رہی ہے؟ |
| *Impact on reproductive health* | 1. Do you believe HPV vaccine can interfere with reproductive health? | The biggest fear we hear is related to infertility. People worry that this vaccine might prevent women from becoming mothers in the future. This kind of fear isn’t new—when we were children, similar concerns were raised about polio drops. (T2) | This item explores myths or fears about vaccines affecting girls' reproductive ability. It helps identify misinformation that may drive resistance. | کیا آپ سمجھتے ہیں کہ  HPV ویکسین تولیدی صحت پر منفی اثارت ڈال سکتی ہے؟ |
| *Trust in newly introduced vaccines* | 1. How much do you trust the HPV vaccine that is being introduced in your country? | There will certainly be resistance from some, but many parents today are much more informed than before. A lot of change has come in Pakistan. Many parents now understand medical advancements and want to make informed choices for their children. So, I would say it’s a 50-50 situation—some will support it, while others might still be hesitant. (T6) | This item measures public confidence in the safety and effectiveness of a newly introduced vaccine. It reflects national-level trust in health systems and global partnerships. | آپ اپنے ملک میں متعارف کی جانے والی  HPV ویکسین پر کتنا اعتماد کرتے ہیں؟ |
| *Perceived knowledge about HPV vaccine* | 1. How well informed do you feel about the benefits of the HPV vaccine?   Potential side effects | One of the most significant things I learned was that cervical cancer is currently the only form of cancer for which a vaccination exists. Unlike other types of cancer, which usually begin treatment only after symptoms appear, this vaccine offers preventative protection. It was emphasized that early vaccination—especially among school-aged girls—can prevent the disease from developing altogether. (T2) | This item assesses how informed individuals feel about the vaccine’s benefits. It provides insight into the effectiveness of information dissemination and potential information gaps. | کیا آپ کو لگتا ہے کہ آپ  HPV ویکسین کے فوائد کے بارے میں مکمل طور پر باخبر ہیں؟ |
| *Perceived effectiveness of the HPV vaccine* | 1. To what extent do you believe the HPV vaccine is effective in preventing cervical cancer? | From what I understand, cervical cancer seems to be becoming more common in Pakistan. If there’s a vaccine that can help prevent it, then I think that’s a good thing. (A4) | This item evaluates belief in the vaccine’s protective function. It is critical to understand motivation for uptake based on perceived clinical benefits. | آپ کس حد تک سمجھتے ہیں کہ HPV ویکسین سروائیکل کینسر کے خلاف تحفظ فراہم کرتی ہے؟ |
| *Perceived importance of preventive health measures* | 18.To what extent do you agree with the following statement: “Early screening and preventive actions (such as vaccinations) are essential to prevent serious diseases in women.” | During the session, they talked about Angelina Jolie’s medical decision-making. It was shared that due to a strong family history of cancer – starting from her mother and grandmother – she made the brave choice to undergo preventive surgeries. Since she was a model and public figure, removing her ovaries was seen as a huge and bold step. But she did it to protect her health. This story was used to emphasize the importance of early diagnosis and preventive action. (FDG1) | This item measures support for preventive healthcare, including vaccination. It helps identify general openness to proactive health-seeking behaviors. | آپ اس بات سے کس حد تک متفق ہیں  خواتین:  میں  سنگین بیماریوں سے بچاؤ کے لئے ابتدائی اسکریننگ اور حفاظتی اقدامات جیسے ویکسینیشین ضروری ھیں؟ |
