# Supplementary material for: From global to local: Developing a context-specific BeSD-HPV tool through cultural and linguistic adaptation in Pakistan
Source: PLoS One. 2026 Jun 15;21(6):e0350162. doi: 10.1371/journal.pone.0350162 (PMC13268181; doi:10.1371/journal.pone.0350162)
Supplement: S2 Table — (DOCX) [file pone.0350162.s006.docx]

| **BeSD (TPB Domain) : Motivation(Intention)** | | | | |
| --- | --- | --- | --- | --- |
| **Construct** | **Survey item** | **Verbatim** | **Rationale** | **Urdu question** |
| 1. Fear based motivation | Would knowing that cervical cancer is increasing among young women in your area motivate you to get your daughter vaccinated with HPV vaccine? | The people who were already affected or if they had any disease, they were getting vaccinated, because of COVID. (HCW6)  Maybe it’s a human psyche that until they will not see its adverse effect, they will not believe in it. (P2) | This item assesses motivation triggered by awareness of rising local disease burden i.e. cervical cancer. It underscores how perceived risk in one's surroundings influences intention to vaccinate. | اگر آپ کو پتہ چلے کہ آپ کے علاقے میں نوجوان لڑکیوں میں سرویکل کینسر کے کیسز بڑھ رہے ہیں تو کیا یہ معلومات آپ کو اپنی بیٹی کو ویکسین HPV لگوانے پر آمادہ کرے گی ؟ |
| 2.Government mandate | If the government made the HPV vaccine mandatory, would you get your daughter vaccinated? | Because we are employed and it was the requirement of workplace (covid vaccination). Maybe I would not have been vaccinated either. (P3)  They just need official documentation—like a written card—to feel assured and go ahead with the vaccination. (HCW10) | This item underscores motivation derived from policy enforcement and how external authority and requirement affect vaccine decision-making. | اگر حکومت ویکسین HPV کو لازمی قرار دے دے تو کیا آپ اپنی بیٹی کو ویکسین لگوائیں گے ؟ |
| 3.Impact of prior negative experience on future vaccine uptake | If one of your children experienced side effects from a vaccine, how likely would you be to consider HPV vaccination for your daughter? | ….my child died when he is 4 months old due to meningitis. But after that, my husband and I did not give importance to vaccinating the rest of the children. (P5)  On one occasion, one of my children developed a lump following vaccination, which led my father-in-law—despite being educated—to initially oppose further vaccinations. (P7) | This item assesses how past adverse events shape future vaccine uptake and explores the effect of previous experiences in decision making. | اگر آپ کے کسی بچے کو ویکسین سے ضمنی اثرات ہوئے ہوں تو کیا آپ اپنی بیٹی کو ویکسین HPV لگوانے کا فیصلہ کریں گے ؟ |
| 4.Intention to vaccinate | How likely are you to register your daughter for the HPV vaccine when the campaign begins? | We’re currently engaged in registration of girls aged 9 to 14 for the HPV vaccine. A large-scale awareness and registration drive is going on. Outreach teams are going door to door, collecting data – asking how many children are in each household. Specifically, how many girls fall into the 9 to 14 age range.  People are showing great interest and are voluntarily providing their information. In fact, many are proactively asking when the campaign will officially start because they are eager to get their daughters vaccinated. (FGD1) | This item assesses the respondent’s readiness to act when vaccination becomes available. It reflects intrinsic motivation, which is key predictors of uptake. | جب  ویکسین HPV  کی مہم کا آغاز ہو گا، تو کیا آپ اپنی بیٹی کو رجسٹر کرانے کا ارادہ کریں گے؟ |
| 5.Perceived importance of HPV vaccination | Do you think vaccinating girls against HPV is as important as other childhood immunizations? | We are not rigid in our stance, it’s just that at the moment, HPV vaccine hasn’t felt essential. (T1) | This item captures how HPV vaccination is prioritized relative to other vaccines. It helps assess whether people view it as equally essential for adolescent girls. | کیا آپ سمجھتے ہیں کے لڑکیوں کو  ویکسین HPV  لگوانا دیگر بچوں کی ویکسینز جتنا ہی اہم ہے؟ |
| 6.Comparative confidence in vaccine safety | How confident are you in the safety of the HPV vaccine compared to other vaccines? | If a safe and effective vaccine exists, and we’re informed about it properly, then I see no reason to hesitate. I would get vaccinated immediately—for my children—because prevention is always better than treatment.  (P6) | This item assesses trust in the safety of the HPV vaccine in relation to more familiar vaccines. It provides insight into hesitancy stemming from limited local experience. | دوسری ویکسینز کے مقابلے میں آپ کو  ویکسین HPV کے محفوظ اثارت پر کتنا بھروسہ ہے؟ |
| 7.Influence of scientific information on vaccination intention | Would you consider getting your daughters vaccinated against HPV if you received evidence-based information? | A transparent presentation of clinical research and case studies would build my confidence. If the data shows a strong positive correlation between vaccination and a reduced risk of cervical cancer, I will feel fully empowered to advocate for it. I would be able to tell students and their parents, “According to research, this is a proven way to protect your health.” Nothing is more effective than prevention — and if we act today, we can avoid future health crises for the next generation. (T3) | This item assesses whether credible, evidence-based communication can positively shift vaccine acceptance. It highlights the role of trusted knowledge in motivating action. | اگر آپ کو ویکسین HPV کے بارے میں سائنسی شواہد پر مبنی معلومات ملیں، تو کیا آپ اپنی بیٹی کو ویکسین لگوانے پر غور و فکر کریں گے؟ |
| 8.Global uptake as motivator | Would learning that the HPV vaccine is widely administered in other countries make you more likely to trust the HPV vaccine? | If I see that it’s being given in foreign countries and that it’s safe and effective, then I would feel more reassured. (P1) | This item evaluates whether global acceptance influences local trust. It captures the role of international trends as a confidence-building factor in vaccination. | اگر آپ کو پتہ چلے کے دیگر ممالک میں ویکسین HPV لگائی جا رہی ہے، تو کیا اس سے آپ کا ویکسین پر اعتماد بڑھے گا؟ |
