# Supplementary material for: From global to local: Developing a context-specific BeSD-HPV tool through cultural and linguistic adaptation in Pakistan
Source: PLoS One. 2026 Jun 15;21(6):e0350162. doi: 10.1371/journal.pone.0350162 (PMC13268181; doi:10.1371/journal.pone.0350162)
Supplement: S3 Table — (DOCX) [file pone.0350162.s007.docx]

| **BeSD (TPB Domain): Social processes (Subjective Norms)** | | | | |
| --- | --- | --- | --- | --- |
| **Construct** | **Survey item** | **Verbatim** | **Rationale** | **Urdu question** |
| Consultation with adolescent girls | 5.Would you discuss with your daughter about the HPV vaccination before deciding to get her vaccinated? | Regarding adolescent girls, it is important that they are informed and educated about the vaccine, so they understand its benefits. However, when it comes to making the final decision about receiving the injection, I believe that responsibility ultimately lies with the parents, not the child. (HCW5)  I mean, it's okay to tell them, but you can't tell them so openly that it is sexually transmitted. But their mothers can be counseled. It’s just like any other vaccinations for them. (HCW7) | This item explores the extent to which parents involve adolescent girls in their health-related decisions, particularly HPV vaccination. It reflects cultural readiness to engage young girls in health matters. | کیا آپ  ویکسین HPV لگوانے کا فیصلہ کرنے سے پہلے اپنی بیٹی سے اس بارے میں بات کریں گے ؟ |
| Vaccine Hesitancy Due to Lack of Community Experience | 6.Would you wait and see how the HPV vaccine affects others before deciding to get it for your daughter? | Before going to have that vaccine, I would personally like to do some research. I would like to see what are the results of the vaccines that have been used in other countries, even in Pakistan. If we get some stats, it will be helpful for me to make a decision. (P2) | This item assesses reliance on others' experience before making vaccination decisions. It reflects the role of community influence in HPV vaccine acceptance. | کیا آپ اپنی بیٹی کو  ویکسین HPV لگوانے سے پہلے انتظار کریں گے تاکہ دیکھ سکیں کہ دوسروں پر اس کا کیا اثر ہوتا ہے ؟ |
| Preference for healthcare provider for communication | 7.Do you want that a female doctor should be the primary person to explain about the HPV vaccine? | We can discuss this matter with our female gynecologist, so that before we go on to having that vaccine, we can make sure that there are no side effects. (P2) | This item measures trust and comfort in receiving vaccine-related information from a female physician. It highlights preferences in people who would prefer to communicate information around sensitive topics. | کیا آپ چاہتے ہیں کہ  ویکسین HPV کے بارے میں معلومات فراہم کرنے والی بنیادی شخصیت خاتون ڈاکٹر ہو ؟ |
| Perceived influence of marital status on vaccine acceptance | 8.Do you think there will be a difference of opinion regarding HPV vaccine among married and unmarried women? | We’ve managed to motivate married women to complete up to three doses, but unmarried girls still don’t get vaccinated. They believe it’s only necessary during pregnancy. (FGD1)  The first thing parents will likely say is that their daughter is unmarried at present, and God knows what exactly is being injected into her. They'll worry it could cause problems for her in the future. (FGD1) | This item assesses how marital status shapes the need for vaccination. It explores social norms and beliefs about vaccine relevance based on life stage. | کیا آپ سمجھتے ہیں کہ شادی شدہ اور غیر شادی شدہ خواتین کی راۓ  ویکسین HPV کے بارے میں مختلف ہو سکتی ہے ؟ |
| Influence of religious beliefs on vaccine decision-making | How do religious beliefs in your community affect decisions about HPV vaccination? | In our village, if religious figures like peers or fakirs, or respected elders in the community, recommend the vaccine, then people are more likely to be convinced. Their word holds a lot of influence here. (P7) | This item explores how religious interpretations and values influence HPV vaccine acceptance or resistance. It captures both positive and negative faith-based social norms. | آپ کی کمیونٹی میں مذہبی عقائد HPV ویکسین سے متعلق فیصلوں پر کس حد تک اثر انداز کرتے ہیں؟ |
| Trusted sources of vaccine information | Who do you trust most to give accurate information about HPV vaccine? | If a doctor I know and trust, especially someone in my family, supports the vaccine, I would consider it. But I wouldn't just rely on general advice from strangers or less familiar medical professionals. (A4) | This item identifies which community figures are most trusted for conveying accurate vaccine-related information. | آپ  HPV ویکسین کے بارے میں درست معلومات کے لئے سب سے زیادہ کس پر بھروسہ کرتے ہیں؟ |
| Willingness to advocate for vaccination | How likely are you to recommend HPV vaccine to others if you choose it for your daughter? | If this vaccine has passed clinical trials and has shown effectiveness in reducing the risk of cervical cancer, then I believe it should be welcomed. Protecting girls within the 9 to 16 age group is a priority. As a society, we must embrace scientifically backed medical interventions, especially when they can safeguard lives. (T2) | This item explores whether personal vaccine acceptance leads to proactive recommendation to others, capturing spillover influence and social diffusion. | اگر آپ اپنی بیٹی کو HPV ویکسین لگواتے ہیں تو کیا آپ دوسرے لوگوں کو بھی یہ ویکسین لگوانے کا مشورہ دیں گے؟ |
| Trust in digital sources within community | Would people in your community trust information about HPV vaccine they come across through social media? | I observed that students were not only willing to get vaccinated, but also proudly shared their vaccination status on social media. This had a ripple effect and helped motivate their peers to do the same. (T4) | This item captures perceived trustworthiness of social media information at the community level, helping assess potential for misinformation or digital health promotion. | کیا آپ کی کمیونٹی کے لوگ سوشل میڈیا پر HPV ویکسین کے بارے میں ملنے والی معلومات پر بھروسہ  کریں  گے؟ |
| Exposure to community-level misinformation | How often do you hear HPV vaccine-related rumors or myths in your community? | Most likely, resistance will come from **mothers**, especially if they’re not fully informed. They may have concerns about how the HPV vaccine could affect their daughter’s health or future, including issues like fertility. (T4) | This item measures how frequently individuals are exposed to rumors or myths, offering insight into the information environment surrounding HPV vaccination. | آپ کو اپنی کمیونٹی میں HPV ویکسین سے متعلق افواہیں یا غلط فہمیاں کتنی بار سُنے کو ملتی ہیں؟ |
| Influence of trusted community recommendations | How likely are you to get your daughter vaccinated if someone you trust in your community recommends it? | Community behavior does play a role. If we observe that others around us are getting their daughters vaccinated, we may also feel encouraged. On the other hand, if no one is doing it, or if there’s little awareness, people tend to assume it's not important. Visibility and public acceptance are key. (T1) | This item assesses the effect of interpersonal trust on motivation to vaccinate. | اگر آپ کی کمیونٹی کا کوئی ایسا شخص جس پر آپ بھروسہ کرتے ہیں HPV ویکسین کی سفارش کرے تو کیا آپ اپنی بیٹی کو یہ ویکسین لگوانے پر راضی ہوجایں؟ |
| Sources of vaccine-related information | Which of the following sources have provided you with information about HPV vaccine? (Tick all that apply) | I believe **s**preading information through social media is far more impactful today. Most people have smartphones and are constantly using platforms like Facebook, WhatsApp, or YouTube. A strong campaign on these platforms would reach a wider audience and could be more effective in delivering accurate information.  (P6) | This item maps out the diversity of information channels (formal and informal) influencing public understanding of HPV vaccine. | آپ کو  HPV ویکسین کے بارے میں معلومات کن ذرائع سے ملتی ہیں؟ |
