# Supplementary material for: From global to local: Developing a context-specific BeSD-HPV tool through cultural and linguistic adaptation in Pakistan
Source: PLoS One. 2026 Jun 15;21(6):e0350162. doi: 10.1371/journal.pone.0350162 (PMC13268181; doi:10.1371/journal.pone.0350162)
Supplement: S4 Table — (DOCX) [file pone.0350162.s008.docx]

| **BeSD domain(TPB construct) :Practical Issues(perceived behaviour control)** | | | | |
| --- | --- | --- | --- | --- |
| **Construct** | **Survey item** | **Verbatim** | **Rationale** | **Urdu Question** |
| Trust in vaccination service quality at health facilities | Would you choose to get your daughter vaccinated if the hospital staff provides limited information about HPV vaccination? | They talk to you in a very good way. Even if there is overcrowding, there are 3 to 4 people who manage the patients well. I have a very good experience with them. (P4)  …hamaray pass itna time nhn hota kay hum un say (people who come to get vaccinated) detail main baat kar sakain. (HCW10) | This item assesses how limited communication of information regarding the HPV vaccine by staff may affect trust in vaccination at public hospitals. | اگر ہسپتال کا عملہ  ویکسین HPV  کے بارے  میں محدود معلومات فراہم کرے تو کیا آپ اپنی بیٹی کو ویکسین لگوانے کا انتخاب کریں گے ؟ |
| Trust in outreach vaccination service safety | Do you trust outreach vaccination services to administer your daughter the HPV vaccine? | People trust us a lot. Whenever a new vaccine comes, they ask us about its safety. The women tell us that their husbands insist to ask us first. Then we tell them that this vaccine is good for them. (FGD1)  Injection safety is a must for injection so, we don't go to every house. We sit at a point. We have social mobilizers. They knock on the house and move the target population towards the center and there is vaccination there. This is your outreach. (PM2) | This item explores trust in the safety of vaccines delivered via outreach teams. | کیا آپ اپنی بیٹی کو  ویکسین HPV لگوانے کے لیے آؤٹریچ ویکسینیشن سروسز پر بھروسہ کرتے ہیں ؟ |
| Choice of vaccine facility | Where would you prefer your daughter to receive the HPV vaccine?"  1.Public health facility  2.Private health facility  3.During an outreach visit  4.At school  5.Other (please specify) | As a pediatrician, I personally prefer government vaccines because I trust their source and reliability. I would choose them for my own children as well. (HCW5)  We prefer government hospitals for vaccinations although people say that there is a lot of inconvenience in government hospitals. There is overcrowding there, and they have to facilitate everyone, so it is understandable but in government hospitals, doctors are experts. (P5) | This item identifies preferred vaccine delivery settings (public, private, outreach, school) to ensure appropriate planning for HPV vaccine implementation. | آپ اپنی بیٹی کو ویکسین HPV کہاں لگوانا پسند کریں گے: سرکاری ہسپتال ؛ نجّی ہسپتال ؛ اؤٹریچ ویکسینیشن ؛ اسکول ؛ دیگر |
| Incentivization of HPV vaccination program | Would people be more willing to accept the HPV vaccine if the government offered monetary benefits? | In Sindh, for instance, people are getting money for vaccinating their children. So some ask us, “Baji, can we get money too?” Once people see such things online or in newspapers, they bring it up with us. (FGD1)  There seems to be a perception that vaccines are linked to financial incentives. People come to us saying they’ve completed their vaccinations under the Aghosh program and are now asking when they will receive the promised money. (HCW10)  Although girls are not usually sent to school consistently, parents make the effort when they know such incentives are provided—for instance, receiving 2–3 liters of cooking oil after a month of attendance. Similarly, if people are offered incentives and awareness programs are actively implemented, I believe people would become more receptive. (HCW8) | This item assesses how offering incentives might influence vaccine acceptance. | اگر حکومت ویکسین HPV لگوانے پر مادی فوائد دے تو کیا لوگ ویکسین لگوانے کے لیے زیادہ تیار ہوں گے ؟ |
| Safety concerns about vaccines administered in educational facilities | Do you trust that HPV vaccine will be given safely in schools? | I think people will cooperate less because when my children were young, they used to come for polio or for the vaccine for diarrhea, most of the time parents refused because they were mostly concerned that they didn't know if vaccine had reached there safely or not. Or who is administering it. So, I don't think this will be very effective. I mean, maybe you can say it is good to give awareness there, but people will not get vaccinated like this. (P9) | This item captures parental confidence in the safety of school-based vaccination programs. | کیا آپ کو اعتماد ہے کہ  ویکسین HPV اسکولوں میں محفوظ طریقے سے دی جائے گی ؟ |
| Creating awareness through educational institutions | Do you think that girls should be given awareness about the HPV vaccine in schools? | It’s more effective to conduct sessions in schools and colleges. When children hear something and understand it, they talk to their mothers about it. That’s how awareness spreads and families become convinced. (FGD1)  There is a need to counsel school-going girls and raise their awareness about health through dedicated health education programs. This is essential to achieve meaningful impact. (HCW2)  This point of contact is very good like we have girls’ colleges, women universities, they have females and their societies so seminars can be conducted, and girls can be counselled. (P2) | This item assesses support for engaging schools as a setting to raise awareness about the HPV vaccine. | کیا آپ سمجھتے ہیں کہ لڑکیوں کو اسکولوں میں ویکسین HPV کے بارے میں آگاہی دی جانی چاہیے ؟ |
| Concern about the brand of vaccine | To what extent do you agree that people are concerned about the brand of the vaccine being offered? | Some claimed that the Pfizer vaccine wasn’t good and advised others not to take it. On the other hand, others said their mother had received it and had no issues, so they chose the same brand. (FGD1) | This item addresses how perceptions of specific vaccine brands influence trust and decision-making. | کیا آپ اس بات سے متفق ہیں کہ لوگ دی جانے والی ویکسین کے برانڈ کے بارے میں فکرمند ہوتے ہیں ؟ |
| Cooperation by educational institutions | To what extent do you think school administration would be willing to cooperate with the vaccination team in implementing the HPV vaccine program? | When there is a campaign, we get letters from our office which we have to give in advance, that we have a campaign and we have to come to the school and vaccinate the children…. If some schools refuse, then our area in charge of the person goes and talks to them that you cannot refuse. (FGD1) | This item highlights potential support or resistance at the institutional level. | کیا آپ سمجھتے ہیں کہ اسکول انتظامیہ  ویکسین HPV پروگرام کو فعال بنانے میں تعاون کرے گی ؟ |
| Attitude difference between public vs. private schools | To what extent do you agree that public and private schools differ in their attitude toward vaccination campaigns? | We go to all schools, Government, private but Private schools bother us a lot. (FGD1) | This item investigates perceived differences in institutional openness to vaccination efforts. It can inform tailored engagement strategies for different school types. | کیا آپ اس بات سے متفق ہیں کہ سرکاری اور نجی اسکول ویکسینیشن مہمات کے بارے میں مختلف رویّہ رکھتےہیں ؟ |
| Targeted teachers’ trainings | Do you think the teachers should have separate trainings on how to create awareness about HPV vaccine among adolescents? | They didn't have a separate training program for teachers (during COVID). (FGD1)  Teachers play a key role. They have clarity, and when parents ask questions, teachers can explain things to the girls. Students tend to listen to their teachers, so involving them is very important. (FGD1)  Teachers need to be fully informed, so they feel confident about what they’re sharing. (P5) | This item highlights the perceived need for teacher-focused capacity-building. | کیا آپ سمجھتے ہیں کہ اساتذہ كو نوجوان لڑکیوں میں  ویکسین HPV کی آگاہی پیدا کرنے کے لیے الگ تربیت دی جانی چاہیے ؟ |
| Concerns about vaccine safety | Would you get your daughter, the HPV vaccine seeing that it has limited prior use in Pakistan? | There are no studies. People have not reported that they have been vaccinated for 10 or 15 years and there is no problem. No one has reported what will happen after being vaccinated in the long term or even in the short term. (P10) | This item explores concerns about long-term safety due to the vaccine's limited use in the country and would help identify the role of uncertainty and fear of unknown side effects in influencing vaccine uptake. | کیا آپ اپنی بیٹی کو  HPV ویکسین لگوائیں گے اگرچہ یہ پاکستان میں ابھی تک محدود پیمانے پر استعمال ہوئی ہے |
| Structural barriers to vaccine access | What challenges might prevent someone in your community from accessing the HPV vaccine?  **Options:** Long distance, lack of information, work/family duties, unavailability, transport, clinic hours) | Accessibility can be a major issue. Even from my own personal experience, I’ve noticed that my family tends to be quite reluctant when it comes to visiting hospitals. This reluctance — whether it’s due to distance, lack of transportation, or just general hesitation about medical visits — can prevent people from seeking vaccination. (A3)  Even if people want to get vaccinated, they might not be able to afford it. There are also hidden costs—travel expenses, time off from work, etc.(T6) | This item identifies logistical barriers that reduce access to HPV vaccination and would help reveal context-specific service delivery gaps that may hinder HPV vaccination uptake. | آپ کے خیال میں آپ کی کمیونٹی میں ویکسین تک رسائی میں کون سی رکاوٹیں ہو سکتی ہیں ؟ |
| Perceived vaccination staff cooperation | Is the vaccination staff cooperative during the vaccination process? | We’ve often noticed that some nurses behave quite rudely. When a vaccination camp is set up and there’s a large crowd, the environment becomes stressful. To avoid that discomfort and the harsh attitude of the staff, some people simply choose to leave without getting vaccinated. (P3)  There was no proper guidance from the hospital staff, and they weren't very cooperative. No one briefed us or explained the process clearly. (P7) | This item assesses interpersonal aspects of vaccine delivery. It reflects how respectful, cooperative behavior by staff influences vaccine experience and acceptability. | کیا ویکسینیشن کا عملہ دورانِ ویکسینیشن آپ کے ساتھ تاون کرتا ہے ؟ |
| Perceived waiting time for **vaccination** services | How long do you typically have to wait at the health center for vaccination services? | On busy days, there were long queues, sometimes a wait of 20 to 30 minutes or more. So, it could be tiring, especially with young children. (P2)  One of the main problems was the long waiting hours. They often had to stand in line for hours just to be seen. (P7) | This item captures respondents’ experience with waiting times at vaccination centers. Understanding perceived or actual delays helps identify service-related factors that may affect vaccine-seeking behavior and user satisfaction. | عام طور پر آپ کو ویکسینیشن کی سہولت کے لیے ہیلتھ سینٹر میں کتنی دیر اِنتظار کرنا پڑتا ہے؟ |
| Effectiveness of reminders in supporting timely vaccination | How helpful would reminders (e.g., mobile messages, school announcements) be in ensuring timely HPV vaccination? | If similar, frequent advertisements are aired about the benefits and safety of the HPV vaccine—on TV, in dramas, through celebrities or influencers, and across social media—it will really help us.  It will not only normalize the idea of the HPV vaccine in people's minds, but it will also reduce resistance and hesitation. Repetition builds familiarity, and familiarity leads to acceptance. If the public keeps seeing positive, informative content about this vaccine, especially content that is easy to understand and culturally relevant, it will make our jobs much easier as field workers. It would sensitize the entire community and increase trust in the vaccine. (FGD1) | This item explores the perceived usefulness of reminders. It helps assess how communication strategies may improve on-time vaccine uptake. | آپ کے خیال میں بروقت ویکسینیشن کو یقینی بنانے کے لیے یاد دہانی( جیسےموبائل پیغام، سکول میں اعلان) کتنی مددگار ہوگی؟ |
| Preferred digital platforms for vaccine related information | Which social media platform would you trust for receiving information about HPV vaccination? | Until we create a culture of **c**ommunity-level conversation and education, and leverage platforms like social media to raise awareness, I don’t think people will easily accept this initiative. For instance, I follow a YouTube channel called *Physics Girl*, which uses science communication effectively — we need similar content creators to explain medical topics like this in a relatable way. (T4) | This item identifies trusted sources of digital information to map the audience engagement across platforms. | HPV آپ ویکسین سے متعلق معلومات حاصل کرنے کے لیے کن سوشل میڈیا پلاٹف فارم پر سب سے زیادہ بھروسہ کرتے ہیں؟ جتنے مناسب ہوں، منتخب کریں |
| Exposure to vaccine-related messaging | How frequently do you receive vaccine-related information through TV, radio, or mobile phones? | Our government has an active social media team and news channels that reach wide audiences. Until they broadcast informative programs or advertisements explaining this disease clearly, both males and females will remain unaware.  Both men and women use social media and watch TV — in fact, men tend to watch a lot of TV. If the government targets these platforms to inform the public, men will better understand the vaccine’s importance and take positive steps to support it.  However, if information is directed only toward females, men may remain uninformed.  (T4) | This item measures how often respondents are exposed to HPV vaccine messages through different channels. It provides insight into information reach. | آپ کو ٹی**-**وی، ریڈیو یا موبائل فون کے ذریعے ویکسین سے متعلق معلومات کتنی بار موصول ہوتی ہیں؟ |
