# Supplementary material for: From global to local: Developing a context-specific BeSD-HPV tool through cultural and linguistic adaptation in Pakistan
Source: PLoS One. 2026 Jun 15;21(6):e0350162. doi: 10.1371/journal.pone.0350162 (PMC13268181; doi:10.1371/journal.pone.0350162)
Supplement: S5 Table — (DOCX) [file pone.0350162.s009.docx]

| **Cultural Integration(Cross cutting Domain)** | | | | |
| --- | --- | --- | --- | --- |
| **Construct** | **Survey item** | **Verbatim** | **Rationale** | **Urdu question** |
| Inclusion of men in vaccine awareness efforts | Do you think males should receive information about the HPV vaccine through awareness campaigns? | A significant barrier is the influence of men in decision-making, especially regarding family planning and vaccination, which further hinders data accuracy and effective healthcare delivery. (PM1)  Men should be targeted by their families or their doctors. Because men here don't talk much about these things. So, it is possible that they may not talk so openly in their homes. (P9) | This item explores impact of involving men in vaccine awareness campaigns and reflects the influence of male decision-making on uptake of HPV vaccine by their daughters. | کیا آپ سمجھتے ہیں کہ مردوں کو ویکسین HPV کے بارے میں آگاہی مہمات کے ذریعے معلومات دی جانی چاہئیں ؟ |
| Role of men in uptake of HPV vaccine | Do you think fathers would be willing to have their daughters vaccinated against HPV?  Decision is in hand of father | This point is the most difficult in this whole scenario, you have to convince a father for something he has never suffered and will not even suffer it ever in the future. In addition, he has never even had such an experience. (P2)  He (father) mentioned that a previous vaccination had led to fever and diarrhea in the child, which required a visit to the doctor and incurred significant expenses. As a result, he was hesitant to have the child vaccinated again. (P7)  if I give an opinion, then yes. If I don't get permission from home, then I can't do anything. (P3) | This item assesses paternal willingness to accept HPV vaccination for daughters. It provides insight into male influence on health-related decisions. | کیا آپ سمجھتے ہیں کہ والد حضرات اپنی بیٹیوں کو ویکسین HPV لگوانے پر رضامند ہوں گے ؟ |
| Cultural Acceptability of Reproductive Health Discussions | How comfortable do you feel talking about reproductive health during family discussions? | In our society, however, such topics are often avoided even when addressing children in religious contexts. Hence, it is important to recognize that this silence is a product of social norms rather than religious teachings and discussing these topics openly from an Islamic standpoint can be a positive step forward. (HCW5)  They do not discuss such things (symptoms of cervical cancer) with anyone. They do not trust anyone or share their reproductive health issues. (HCW6)  Hum aisy gandi baatain nhn krtay. (Informal interview with a father)  In our society we don’t discuss these matters with children. Maybe mothers maybe doing it with daughters, so I don’t know but as far as fathers are concerned, they don’t talk about these things with 15 or 16 years old. | This item evaluates openness to discussing sensitive reproductive health topics within families and how it may influence HPV vaccine awareness and acceptance. | کیا آپ خاندانی گفتگو کے دوران تولیدی صحت کے موضوع پر بات کرنے میں آرامدہ محسوس کرتے ہیں ؟  mutmain |
| Perceived ethnic variation in vaccine hesitancy | Do you think people from different ethnic backgrounds (such as Pathans or Afghans) may be hesitant about getting HPV vaccine for their daughters? | Pathan families here strictly refuse vaccination so then we try to persuade their men. (FGD1)  I think they (Pathan community) don't even want childhood vaccination. You know, they don't even let you get a polio vaccine at home. (P3) | This item highlights perceptions of vaccine hesitancy across ethnic groups. | کیا آپ سمجھتے ہیں کہ مختلف نسلی پس منظر رکھنے والے لوگ (جیسے پٹھان یا افغان) اپنی بیٹیوں کو  ویکسین HPV لگوانے میں ہچکچاہٹ محسوس کریں گے ؟ |
| Intra-household decision-making authority | Who in your family has the most influence on health-related decisions? | In our home, decisions are typically made by both my father and mother together. They consult each other and, in many cases, include us children in the discussion too. It’s usually a cooperative process where everyone’s opinions are valued before a final decision is made. (A1)  My father usually makes those decisions.  (A2) | This item identifies the key decision-maker within families for health-related actions. | آپ کے گھر میں صحت کے فیصلے اکثر کس کے کہنے پر کیئے جاتے ہیں؟ |
| Cultural barriers to vaccine acceptance | Do you agree that community members will be hesitant to administer HPV vaccine based on cultural reasons? | In our community, honestly, not many people go for vaccination. The general thinking is that whatever is destined to happen will happen. There isn’t much emphasis on prevention. (A2)  Culturally, people tend to hesitate about new things, especially things that haven’t been part of family traditions. For example, no one in our families has ever received this vaccine before, so people may question, “Where did this come from? Why now?” (T2) | This item captures perceived resistance to HPV vaccination based on cultural sensitivities, norms, or taboos. | کیا آپ اس بات سے متفق ہیں کہ کمیونٹی کے افراد ثقافتی وجوہات کی بِنا پر  HPV ویکسین لگوانے سے ہچکچاتے ہیں؟ |
| Perceived gender equity in vaccine policy | Do you think it is fair that only girls are being targeted for the HPV vaccine? | When I hear it’s being given only to females aged 9 to 16, especially unmarried girls, it raises concerns. That approach alone doesn’t reassure me. if the disease stems from a sexually transmitted infection, logically, both males and females could be carriers or affected. That’s what made me skeptical. (A4)  While general immunization programs are accepted to a certain extent, a vaccine like HPV, which is administered only to girls initially, raises more suspicion. People start to wonder why it's being given only to girls and not boys. This kind of gender-specific roll-out makes people more cautious and doubtful, especially in more traditional households.  (P3) | This item captures attitudes about the fairness of gender-specific vaccination, reflecting perceptions of equity and inclusiveness in public health. | کیا آپ سمجھتے ہیں کہ صرف لڑکیوں کو  HPV ویکسین کے لئے چُنّا مناسب ہے؟ |
| Intrinsic Motivation to vaccinate despite family resistance | Would you get your daughter HPV vaccination even if your family opposed it? | She is the only child. I am vaccinated, but her father is very scared for her. Even in Corona, he was told to get her vaccinated, but he refused to get her vaccinated. (P3) | This item assesses the respondent’s intrinsic motivation to vaccinate despite social or family resistance and reflects personal prioritization of health decisions. | کیا آپ خاندان کی مخالفت کی صورت میں اپنی بیٹی کو  ویکسین HPV لگوانے کا انتخاب کریں گے ؟ |
| Influence of peer behavior on vaccination uptake | If people you know got their daughters vaccinated for HPV, would you be more likely to do the same? | When people realized the importance of covid vaccine and saw that no one dies, nothing happens and there is benefit for them so then some people came to us. (HCW3) | This item underlines how observing peers' decisions impacts on personal willingness to vaccinate. | اگر آپ کے جاننے والے لوگ اپنی بیٹیوں کو ویکسین لگوائیں تو کیا آپ بھی ایسا کرنے کے لیے زیادہ تیار ہوں گے ؟ |
| Motivation by role modelling | Would you feel more confident about vaccinating your daughter if you saw health care providers vaccinating their own daughters with HPV vaccine? | We (doctors) are the first to accept and receive the vaccination. For example, during the polio campaigns, when parents were hesitant, I would bring my own children to show them that the vaccine is safe saying, “I’ll vaccinate my child first, and then you can vaccinate yours.” (HCW3) | This item highlights the impact of trusted role models (healthcare providers). It explores how seeing trusted figures act first increases public confidence and uptake of HPV vaccine. | اگر آپ یہ دیکھیں کہ ہیلتھ کیئر سے تعلق رکھنے والے لوگ اپنی بیٹیوں کو ویکسین HPV لگوا رہے ہیں تو کیا آپ اپنی بیٹی کو ویکسین لگوانے میں زیادہ اعتماد محسوس کریں گے ؟ |
| Influence of patient stories on vaccine uptake | Would listening to real-life experiences of cervical cancer patients help build parents’ trust in the HPV vaccine? | If you put forward in front of them case studies and real-life examples e.g. these many cases have been reported in your area and the main cause being that they were not vaccinated and they are made to visit such people meaning if they don’t see the real problem they would not opt for vaccination. (P2) | This item explores how personal stories shape vaccine perceptions and inturn effect the HPV vaccine uptake. | کیا آپ سمجھتے ہیں کہ سروائیکل کینسر کے مریضوں کے حقیقی واقعات سن کر والدین کا ویکسین HPV پر اعتماد بڑھے گا ؟ |
| Consent taking | Would you be willing to get your daughter HPV vaccination in schools without parental consent? | Schools have a system where they circulate the consent form If parents allow it, they will get permission and then they will perform it. (P6)  We will give information to the parents that on this date your child will be vaccinated, when we go towards consent taking 50% refuse and the school doesn’t let our vaccinators enter the school premises even so we will just give information. (PM2) | This item evaluates willingness to allow school-based vaccination without parental consent. It reflects parental trust in vaccine campaigns carried out in schools. | کیا آپ اس بات پر رضامند ہوں گے کہ آپ کی بیٹی کو اسکول میں والدین کی اجازت کے بغیر  ویکسین HPV دی جائے ؟ |
| Parental decision-making when uncertain | How would you respond to the following situation: "If your daughter wanted to get HPV vaccine but you are unsure about it.” | My parents are strict, and if they say no, then that’s the final word. I wouldn’t go against their decision, no matter how convinced I am. I would do my best to talk to them, share what I’ve learned, explain the pros and cons — try to change their mind. But if they remain firm in their decision, I will have to respect that. (A1) | This item explores vaccine decision-making in situations of parental uncertainty versus adolescent desire. It reflects motivation, autonomy, and intergenerational trust. | اگر آپ کی بیٹی ویکسین HPV لگوانا چاہے لیکن آپ کو اس بارے میں اندیشہ ہو، تو آپ کا ردعمل کیا ہوگا؟ |
| Gender sensitive vaccine delivery | Do you agree that a female vaccinator should be available to administer the HPV vaccine to girls? | Some parents object that we will get vaccinated from a female. (FGD1)  Since the HPV vaccine involves a particularly sensitive age group, we are prioritizing the involvement of female health workers… nurses, wherever possible. (PM2)  No, it won’t be an issue… because we have done a lot of vaccinations for measles, COVID in schools as well. So it won’t be an issue for them. If a girl is shy, we tell her to get vaccinated from a lady. (HCW 3) | This item captures the importance of female vaccinators for adolescent girls, addressing cultural preferences. It underscores gender-sensitive vaccine delivery strategies that may affect acceptability. | کیا آپ اس بات سے متفق ہیں کہ لڑکیوں کو ویکسین لگانے کے لیے خاتون ویکسنیٹر ہونی چاہیئے ؟ |
