# Supplementary material for: From global to local: Developing a context-specific BeSD-HPV tool through cultural and linguistic adaptation in Pakistan
Source: PLoS One. 2026 Jun 15;21(6):e0350162. doi: 10.1371/journal.pone.0350162 (PMC13268181; doi:10.1371/journal.pone.0350162)
Supplement: Attachment — Submitted filename: Review notes.pdf [file pone.0350162.s010.pdf]

Thank you for submitting your manuscript “From Global to Local: Developing a Context-Specific BeSD-HPV Tool through Cultural and Linguistic Adaptation in Pakistan” to PLOS One.

The study offers insights into the cultural, social, and gendered factors influencing HPV vaccine hesitancy in Pakistan, while important methodological and conceptual clarifications are needed to fully support the proposed adaptation of the BeSD framework.

Some revisions are suggested as follows:

Introduction: Please strengthen the Introduction by clearly defining the BeSD-HPV framework, explicitly justifying why Pakistan is the appropriate context, and explaining why the WHO Behavioral and Social Drivers of Vaccination (BeSD) framework is the most suitable approach for addressing HPV vaccination in this setting.

The Introduction does not adequately describe how behavioral, social, and cultural factors specific to Pakistan influence HPV vaccination decisions, nor does it report current HPV vaccination coverage, clarify the target population (female, male, or both, given that HPV vaccination is not limited to cervical cancer prevention), or present sex-specific HPV disease burden and vaccination patterns.

Key terms such as “low-resource environments” are also insufficiently described, and prior HPV prevention or vaccination approaches in Pakistan, along with their limitations related to behavioral and cultural factors, are not clearly described. Addressing these gaps is essential to establish a coherent rationale for the proposed framework development

The figure Methodological Framework for a Culturally Adapted HPV Vaccine Acceptance Study in Pakistan partially reflects the WHO BeSD framework; however, several core BeSD components are incompletely specified, and conceptual boundaries between domains are unclear. In particular, the addition of a “Cultural Integration” domain appears to overlap with existing BeSD constructs without sufficient justification. Please clarify the conceptual rationale for this domain and explicitly describe how it differs from, extends, or integrates with existing BeSD domains.

Please report participants’ HPV vaccination status and indicate whether vaccination experience was considered in sampling or analysis, as the absence of this information limits interpretation of the findings.

Although 4 qualitative interview guides are mentioned, the Methods do not clearly describe how these guides differ in content, what domains each covers, or how they were aligned with the WHO BeSD framework. Please clarify these distinctions and provide sufficient detail to assess how the interview guides operationalized the framework.

Please clarify the composition of all 4 focus group discussions, including the participant group for the fourth session and the approximate number of participants per group.

Please provide greater analytic detail by clarifying how Braun and Clarke's six-step thematic analysis was applied, how coding consistency was ensured across multiple coders, how transcript translation was conducted and verified, and what analytic criteria justified the creation of the additional "Cultural Integration" domain rather than integration within existing BeSD domains.

It seems that the Methods and Results do not sufficiently demonstrate how qualitative findings were systematically synthesized into a distinct, culturally adapted BeSD-HPV framework. In particular, the process for preserving the conceptual integrity of the original BeSD domains and the analytic justification for introducing an additional domain are not clearly articulated, limiting confidence that the stated framework development objective was achieved.

The Discussion partially addresses the Results and the stated study purpose. While it interprets key cultural and social drivers of HPV vaccine hesitancy and reiterates the rationale for adapting the WHO BeSD framework, it lacks a clear, concise summary of the main findings and does not consistently link interpretations back to the reported Results. Comparison with prior BeSD-based studies is limited, and the conceptual contribution of the added "Cultural Integration" domain remains insufficiently articulated. It could be helpful to strengthen the Discussion by improving alignment with the Results, adopting a more cautious framing of the implications, and explicitly addressing future validation and application of the adapted framework.

Please spell out the full term at first mention and use the abbreviation thereafter.

Please clearly identify all supplementary materials and provide complete legends for all figures.

Please review and revise the manuscript to ensure that all components comply with the journal's submission guidelines.
