# Supplementary material for: From global to local: Developing a context-specific BeSD-HPV tool through cultural and linguistic adaptation in Pakistan
Source: PLoS One. 2026 Jun 15;21(6):e0350162. doi: 10.1371/journal.pone.0350162 (PMC13268181; doi:10.1371/journal.pone.0350162)
Supplement: Attachment — Submitted filename: Rebuttal Letter Updated.docx [file pone.0350162.s011.docx]

From Global to Local: Developing a Context-Specific BeSD-

HPV Tool through Cultural and Linguistic Adaptation in

Pakistan

[PONE-D-25-55196] - [EMID:5f87472bf045244e]

PLOS ONE

Dear Editor,

We sincerely thank the reviewers for their thoughtful and constructive comments. We have carefully addressed the points raised and incorporated most of the suggestions in the revised manuscript. We believe that these revisions have substantially improved the clarity, rigor, and overall quality of the paper.

We remain fully open to further revisions and would be pleased to address any additional comments or recommendations.

Please find the modified manuscript. We have incorporated all suggestions recommended by the reviewers. Following is the point-wise reply to the reviewers' comments.

With best regards,

Dr Noor

| Journal Requirements | | |
| --- | --- | --- |
| Comments | Response | Reference |
| 1. Please ensure that your manuscript meets PLOS ONE's style requirements, including those for file naming. The PLOS ONE style templates can be found at  <https://journals.plos.org/plosone/s/file?id=wjVg/PLOSOne_formatting_sample_main_body.pdf> and [https://journals.plos.org/plosone/s/file?id=ba62/PLOSOne_formatting_sample_title_authors_affiliations.pdf](https://track.editorialmanager.com/CL0/https:%2F%2Fjournals.plos.org%2Fplosone%2Fs%2Ffile%3Fid=ba62%2FPLOSOne_formatting_sample_title_authors_affiliations.pdf/1/010f019bc0fc4981-5238811e-0c45-4e32-84b4-627b25579b22-000000/b_eCvNaH30-iu2r8yATSZh4ZvWIkRS8hCBEzoBgP0wI=246) | We have revised the manuscript to fully comply with PLOS ONE formatting guidelines, including file naming, title page formatting, structured sections, and reference style. | Whole document |
| 2. We note that your Data Availability Statement is currently as follows: [All relevant data are within the manuscript and its Supporting Information files]  Please confirm at this time whether or not your submission contains all raw data required to replicate the results of your study. Authors must share the “minimal data set” for their submission. PLOS defines the minimal data set to consist of the data required to replicate all study findings reported in the article, as well as related metadata and methods (<https://journals.plos.org/plosone/s/data-availability#loc-minimal-data-set-definition>).  For example, authors should submit the following data:  - The values behind the means, standard deviations and other measures reported; - The values used to build graphs; - The points extracted from images for analysis.  Authors do not need to submit their entire data set if only a portion of the data was used in the reported study.  If your submission does not contain these data, please either upload them as Supporting Information files or deposit them to a stable, public repository and provide us with the relevant URLs, DOIs, or accession numbers. For a list of recommended repositories, please see <https://journals.plos.org/plosone/s/recommended-repositories>.  If there are ethical or legal restrictions on sharing a de-identified data set, please explain them in detail (e.g., data contain potentially sensitive information, data are owned by a third-party organization, etc.) and who has imposed them (e.g., an ethics committee). Please also provide contact information for a data access committee, ethics committee, or other institutional body to which data requests may be sent. If data are owned by a third party, please indicate how others may request data access. | We confirm that the minimal de-identified dataset required to replicate the findings (coding Tree and Interview Transcripts) has been uploaded as Supporting Information. All identifying information has been removed. All relevant data are within the manuscript and its Supporting Information files. | Refer to Supplementary File 1 and 2 |
| 3. Please upload a new copy of Figure 2 as the detail is not clear. Please follow the link for more information: [https://journals.plos.org/plosone/s/figures](https://track.editorialmanager.com/CL0/https:%2F%2Fjournals.plos.org%2Fplosone%2Fs%2Ffigures/1/010f019bc0fc4981-5238811e-0c45-4e32-84b4-627b25579b22-000000/qVgjzTJPq4wtSRxU_stkV9JcfbfHg0tL7vOG4JCW2PA=246) | We have replaced Figure 2 with a higher-resolution version in TIFF format as per PLOS figure guidelines. | Refer to Figure 2 |
| 4. We note that Figure 3 in your submission contains copyrighted images. All PLOS content is published under the Creative Commons Attribution License (CC BY 4.0), which means that the manuscript, images, and Supporting Information files will be freely available online, and any third party is permitted to access, download, copy, distribute, and use these materials in any way, even commercially, with proper attribution. For more information, see our copyright guidelines: <http://journals.plos.org/plosone/s/licenses-and-copyright>.  We require you to either (1) present written permission from the copyright holder to publish these figures specifically under the CC BY 4.0 license, or (2) remove the figures from your submission:  1. You may seek permission from the original copyright holder of Figure 3 to publish the content specifically under the CC BY 4.0 license.  We recommend that you contact the original copyright holder with the Content Permission Form (<http://journals.plos.org/plosone/s/file?id=7c09/content-permission-form.pdf>) and the following text:  “I request permission for the open-access journal PLOS ONE to publish XXX under the Creative Commons Attribution License (CCAL) CC BY 4.0 (<http://creativecommons.org/licenses/by/4.0/>). Please be aware that this license allows unrestricted use and distribution, even commercially, by third parties. Please reply and provide explicit written permission to publish XXX under a CC BY license and complete the attached form.”  Please upload the completed Content Permission Form or other proof of granted permissions as an "Other" file with your submission.  In the figure caption of the copyrighted figure, please include the following text: “Reprinted from [ref] under a CC BY license, with permission from [name of publisher], original copyright [original copyright year].”  2. If you are unable to obtain permission from the original copyright holder to publish these figures under the CC BY 4.0 license or if the copyright holder’s requirements are incompatible with the CC BY 4.0 license, please either i) remove the figure or ii) supply a replacement figure that complies with the CC BY 4.0 license. Please check copyright information on all replacement figures and update the figure caption with source information. If applicable, please specify in the figure caption text when a figure is similar but not identical to the original image and is therefore for illustrative purposes only. | We have removed the copyrighted image and replaced it with an original figure created by the authors that conveys the same conceptual information while complying with CC BY 4.0 licensing requirements. | Refer to Figure 3 |
| 5. We notice that your supplementary tables are included in the manuscript file. Please remove them and upload them with the file type 'Supporting Information'. Please ensure that each Supporting Information file has a legend listed in the manuscript after the references list. | Supplementary tables have been removed from the manuscript and are being uploaded as a 'Supporting Information', All Supporting Information files are clearly labeled and referenced after the References section. | Supplementary Tables S1, S2, S3, S4, S5 |
| 6. We note that this data set consists of interview transcripts. Can you please confirm that all participants gave consent for interview transcript to be published?  If they DID provide consent for these transcripts to be published, please also confirm that the transcripts do not contain any potentially identifying information (or let us know if the participants consented to having their personal details published and made publicly available). We consider the following details to be identifying information:  - Names, nicknames, and initials - Age more specific than round numbers - GPS coordinates, physical addresses, IP addresses, email addresses - Information in small sample sizes (e.g. 40 students from X class in X year at X university) - Specific dates (e.g. visit dates, interview dates) - ID numbers  Or, if the participants DID NOT provide consent for these transcripts to be published:  - Provide a de-identified version of the data or excerpts of interview responses - Provide information regarding how these transcripts can be accessed by researchers who meet the criteria for access to confidential data, including:  a) the grounds for restriction b) the name of the ethics committee, Institutional Review Board, or third-party organization that is imposing sharing restrictions on the data c) a non-author, institutional point of contact that is able to field data access queries, in the interest of maintaining long-term data accessibility. d) Any relevant data set names, URLs, DOIs, etc. that an independent researcher would need in order to request your minimal data set.  For further information on sharing data that contains sensitive participant information, please see: <https://journals.plos.org/plosone/s/data-availability#loc-human-research-participant-data-and-other-sensitive-data>  If there are ethical, legal, or third-party restrictions upon your dataset, you must provide all of the following details (<https://journals.plos.org/plosone/s/data-availability#loc-acceptable-data-access-restrictions>):  1. A complete description of the dataset 2. The nature of the restrictions upon the data (ethical, legal, or owned by a third party) and the reasoning behind them 3. The full name of the body imposing the restrictions upon your dataset (ethics committee, institution, data access committee, etc) 4. If the data are owned by a third party, confirmation of whether the authors received any special privileges in accessing the data that other researchers would not have 5. Direct, non-author contact information (preferably email) for the body imposing the restrictions upon the data, to which data access requests can be sent  If the reviewer comments include a recommendation to cite specific previously published works, please review and evaluate these publications to determine whether they are relevant and should be cited. There is no requirement to cite these works unless the editor has indicated otherwise. | All participants provided written informed consent for anonymized excerpts to be published. Transcripts were de-identified and do not contain names, specific dates, or identifiable details | Supplementary file no. 2 |
| 1. Conceptual Clarity and Justification  • The Introduction does not sufficiently justify why Pakistan is a distinct context requiring a unique adaptation of the BeSD framework. The sociocultural, religious, and gendered nuances must be explicitly detailed with supporting literature. | Thank you for pointing this out. We have added a new paragraph about Pakistan’s distinct context requiring a unique adaptation of the BeSD framework. The sociocultural, religious, and gendered nuances have been discussed in detail with supporting literature | **Page no. 04**  **Line no.** 77–86 |
| • The added domain “Cultural Integration” lacks clear conceptual distinction from the existing BeSD domains (e.g., Social Processes, Thinking & Feeling). You must provide a rigorous theoretical and empirical justification for why this domain is necessary rather than integrating these constructs into existing domains. Overlap must be explicitly addressed. | Thank you for pointing this out. We have added a conceptual framework and new paragraph explaining it. | Page no. 07 Line no.144–149; 188–197 |
| • The target population (adolescent girls) and the rationale for excluding boys from HPV vaccination discussions need clarification, especially given global recommendations for gender-neutral HPV vaccination. | We appreciate the reviewer’s comment and agree that clarification is needed. We have added a new paragraph in the introduction section. | Page no. 06, Line no.121–129 |
| 2. Methodological Rigor and Transparency  • Participant characteristics: Vaccination status of participants is missing. This is critical for interpreting perspectives on hesitancy vs. acceptance. | We thank the reviewer for highlighting this important point. We agree that participants’ vaccination status is critical for interpreting perspectives on hesitancy versus acceptance.  However, during the data collection period, the HPV vaccination is yet to be included in the national immunization program in Pakistan, and hence none of the study participants (adolescent girls, parents, and community members) had been vaccinated against HPV. Thus, the views of the participants are based on expected acceptance, conditional acceptance, or reluctance toward a new vaccine, instead of the experience related to the previous uptake.  For other routine childhood vaccines, participants represented a **mix of generally vaccine-accepting and vaccine-hesitant backgrounds**, as inferred from their narratives and prior experiences (e.g., polio and COVID-19 vaccination), which are explicitly discussed in the results and discussion sections. | Page no. 11, Line no. 238–242 |
| • Interview guides: The four qualitative guides are mentioned but not described. You must detail how each guide was tailored to different stakeholder groups and how they operationalized the BeSD domains. | We thank the reviewer for this comment and agree that further clarification was needed. In the revised manuscript, we have now explicitly described the four qualitative interview guides and how each was tailored to specific stakeholder groups while operationalizing the BeSD domains.  Adapted Interview guides are also attached as supplementary file no 1 | Page no. 12, Line no.246–258 |
| • Focus Group Discussions: The composition of the fourth FGD is unspecified. Provide participant numbers and group demographics for all FGDs. | We have revised the Methods section to provide detailed information on the composition of all four focus group discussions | Page no. 14, Line no. 289–298 |
| • Analytical process: While Braun & Clarke’s thematic analysis is cited, the manuscript lacks detail on coder agreement, resolution of discrepancies, and translation verification processes. | Thank you for pointing this out.  We thank the reviewer for highlighting this point. We have now expanded the Analysis section to clarify the analytical rigor. **These explanations have been included in order to enhance transparency and credibility of the analysis procedure.** | **Page no. 15, Line no.** 311–321 |
| • Sampling justification: Purposive sampling is noted, but the rationale for selecting specific districts and stakeholders needs elaboration regarding representativeness and transferability. | We thank the reviewer for this comment. We have now clarified the rationale for both **district selection and stakeholder inclusion** to address issues of representativeness and transferability in the method section in the revised version. | **Page no. 11, Line no.** 223–237 |
| 3. Structural and Presentation Issues  • Tables are excessively large and disrupt narrative flow. Consider moving detailed tables (e.g., Tables I-V) to supplementary materials and retaining summarized versions in the main text. | Thank you for pointing this out. We have moved detailed tables (Tables I-V) to supplementary materials by retaining important findings in the result section. | Refer to Supplementary files |
| • Figure clarity: Figures (e.g., methodological framework) require improved labeling and integration with the described | Figure 1, 2 and 3 has been revised for improved clarity, with all domains and steps clearly labeled. The legend has been rewritten to be self-contained, describing all processes. | Refer to Figure 1, 2, 3 |
| • Language and flow: Several sections contain repetitive phrases, grammatical errors, and unclear syntax. A thorough language edit by a native English speaker or professional editing service is strongly recommended. | Thank you for pointing this out. The whole manuscript has been checked for spelling and grammar. General editing has been done throughout the manuscript. Additionally, a thorough language edit has been performed by a professional editing service. | Whole document  Thorough language edit has been performed by a professional editing service. |
| 4. Discussion and Interpretation  • The Discussion does not sufficiently synthesize key findings or explicitly link back to the Results. A concise summary of main themes is needed.  • Comparative analysis with other BeSD adaptation studies (e.g., Nigeria, Bangladesh) is superficial. Strengthen this to highlight the unique contributions of your work. | We appreciate this constructive feedback. We have revised the discussion to include a **concise synthesis of the key findings**, explicitly linking them back to the results. | Page 26, Line no. 521-552  Page 28, Line no 595-610 |
| • The implications of the “Cultural Integration” domain for policy and practice are overstated without validation. Frame conclusions more cautiously and emphasize the need for future quantitative validation of the adapted tool. | Thank you for pointing this out.  We thank the reviewer for this important observation. We agree that the implications of the cultural integration domain should be framed more cautiously at this stage. **In the revised manuscript, we have toned down the policy and practice claims. We have rephrased the text and also added few sentences.** | Page no 29- 30, Line no.624-634 |
| 5. Compliance with Journal Guidelines  • Ensure all supplementary materials are clearly labeled and referenced.  • Abbreviations (e.g., BeSD, LMICs) must be defined at first use.  • Ethical approval details are included but should be clearly stated in the Methods section as per PLOS ONE requirements. | We have checked all supplementary materials are clearly labeled and referenced.  We have checked that abbreviations are mentioned on first use  Thank you for pointing this out. We have included Ethical approval details in the method section as per PLOS ONE requirements. | • Ethical approval Line no.202-204 |
| 1. Restructure the Introduction to clearly articulate the gap in cultural adaptation of BeSD in Pakistan, supported by local data on HPV burden, vaccination coverage, and sociocultural barriers. | Thank you for this suggestion. We have restructured the Introduction as suggested | Page no. 5-6, line no 105-118. |
| 2. Clarify methodological steps in a dedicated subsection, detailing adaptation procedures, coder consistency, translation approach, and participant recruitment. | Thank you for this suggestion. We have clarified methodological steps in a dedicated subsection “data collection tool” as suggested. | Page no 16, line no. 329-338. |
| 3. Refine the conceptual framework to explicitly distinguish the new “Cultural Integration” domain and justify its addition with qualitative evidence. | Thank you for this suggestion. We have refined the conceptual framework to explicitly distinguish the new “Cultural Integration” domain and justified its addition with qualitative evidence as suggested.  **Data analysis** and heading **Thematic analysis** and under **Thematic analysis and Mapping of qualitative findings to BeSD domains in alignment with theory of planned behaviour** | Page no. 14-22  Line no. 307-470 |
| 4. Condense and reorganize results: Use summary tables in the main text and provide extensive tables as supplementary files. | Thank you for this suggestion. We have moved the tables to the supplementary files and briefly summarized the study’s results in the results section, as suggested |  |
| 5. Strengthen the Discussion with a clear summary of findings, comparative context, limitations, and future research directions, particularly tool validation. | Thank you for this suggestion. We have strengthened the discussion as suggested by adding following paragraphs. | Page no. 28-30  Line no. 595-634 |
| 6. Undergo thorough language editing to improve clarity, coherence, and academic tone. | Thank you for pointing this out. The whole manuscript has been checked for spelling and grammar. General editing has been done throughout the manuscript. Additionally, a thorough language edit has been performed by a professional editing service | Whole document |
| Reviewer’s comments | | |
| 1. Is the manuscript technically sound, and do the data support the conclusions?  The manuscript must describe a technically sound piece of scientific research with data that supports the conclusions. Experiments must have been conducted rigorously, with appropriate controls, replication, and sample sizes. The conclusions must be drawn appropriately based on the data presented.  Reviewer #1: Yes  Reviewer #2: Partly | Thank you for your comments. We have further improved the manuscript by incorporating all suggestions. |  |
| 2. Has the statistical analysis been performed appropriately and rigorously?  Reviewer #1: Yes  Reviewer #2: N/A | Thank you for your comments. We have further improved the statistical analysis by incorporating all suggestions. |  |
| 3. Have the authors made all data underlying the findings in their manuscript fully available?  The [PLOS Data policy](http://track.editorialmanager.com/CL0/http:%2F%2Fwww.plosone.org%2Fstatic%2Fpolicies.action%23sharing/1/010f019bc0fc4981-5238811e-0c45-4e32-84b4-627b25579b22-000000/qbQZWf64WhTP4gWYjvVIONTYM95ow07Znqe6yCNLiS0=246) requires authors to make all data underlying the findings described in their manuscript fully available without restriction, with rare exception (please refer to the Data Availability Statement in the manuscript PDF file). The data should be provided as part of the manuscript or its supporting information, or deposited to a public repository. For example, in addition to summary statistics, the data points behind means, medians and variance measures should be available. If there are restrictions on publicly sharing data—e.g. participant privacy or use of data from a third party—those must be specified.  Reviewer #1: Yes  Reviewer #2: No | Thank you for your comments. We have included a sentence about the availability of the data. |  |
| 4. Is the manuscript presented in an intelligible fashion and written in standard English?  PLOS ONE does not copyedit accepted manuscripts, so the language in submitted articles must be clear, correct, and unambiguous. Any typographical or grammatical errors should be corrected at revision, so please note any specific errors here.  Reviewer #1: Yes  Reviewer #2: No | The whole manuscript has been checked for spelling and grammar. General editing has been done throughout the manuscript. Additionally, a thorough language edit has been performed by a professional editing service. |  |
| 5. Review Comments to the Author  Please use the space provided to explain your answers to the questions above. You may also include additional comments for the author, including concerns about dual publication, research ethics, or publication ethics. (Please upload your review as an attachment if it exceeds 20,000 characters)  Reviewer #1: Thank you for submitting this important qualitative study focused on the cultural and linguistic adaptation of the WHO BeSD-HPV tool for Pakistan. The manuscript addresses a clear public health gap, is methodologically sound, and provides a well-articulated adaptation process grounded in rigorous qualitative inquiry.  The study employs a descriptive qualitative design that is appropriate for cultural adaptation work.  Use of Braun & Clarke’s six-step thematic analysis is well described and correctly implemented.  The data (verbatim quotes, themes, emergent domain) clearly support the conclusions.  Addition of a new domain, Cultural Integration, is strongly grounded in participant narratives.  Transparency in coding, construct development, and translation strengthens credibility.  Clear use of both deductive and inductive coding. | Thank you for your comments. |  |
| Trustworthiness is addressed comprehensively using Lincoln and Guba’s criteria (credibility, dependability, confirmability, transferability). | Thank you for your comments. |  |
| Reflexivity is described adequately. | Thank you for your comments. |  |
| Triangulation through IDIs and FGDs strengthens internal validity. | Thank you for your comments. |  |
| Minor Suggestion:  Indicate coder agreement or how discrepancies were resolved (e.g., consensus meetings, intercoder reliability). | Thank you for your suggestions. We have included details on the coder agreement and methods for resolving discrepancies in the revised version of the manuscript. | **Page no 15,**  **Line no 311-321** |
| Clarity and English Language Quality  The manuscript is clear, intelligible, and written in acceptable standard English, but minor language and formatting issues should be addressed.  Examples of recommended improvements:  Remove repeated words (e.g., “that that”, “includees”). | Thank you for this suggestion. The whole manuscript has been checked for spelling and grammar. General editing has been done throughout the manuscript. Additionally, a thorough language edit has been performed by a professional editing service. | **Whole document** |
| Areas for Strengthening Before Publication  Include a brief paragraph on the limitations, such as:  geographic focus on Punjab,  potential social desirability bias,  reliance on purposive sampling. | Thank you for this suggestion. We have rephrased and modified the limitation section by incorporating . are incorporating geographic focus on Punjab, potential social desirability bias, reliance on purposive sampling. | Page no. 30-31  Line no. 645-659 |
| In the Discussion, more explicitly compare findings to other adaptation studies of BeSD tools globally. | Thank you for this suggestion. We have modified the discussion as suggested. | **Page no. 28-29**  **Line no. 595-623** |
| Improve the clarity of tables some are extremely large for the main text. | Thank you for this suggestion. We have moved the large tables as supplementary files. | **Refer to Suplementary Tables S1, S2, S3, S4 and S5** |
| Reviewer #2: Thank you for submitting your manuscript “From Global to Local: Developing a Context-Specific BeSD-HPV Tool through Cultural and Linguistic Adaptation in Pakistan” to PLOS One.  The study offers insights into the cultural, social, and gendered factors influencing HPV vaccine hesitancy in Pakistan, while important methodological and conceptual clarifications are needed to fully support the proposed adaptation of the BeSD framework. | Thank you for this comments. We have modified the manuscript thoroughly. |  |
|  |  |  |
| Some revisions are suggested as follows:   Please strengthen the Introduction by clearly defining the BeSD-HPV framework, explicitly justifying why Pakistan is the appropriate context, and explaining why the WHO Behavioral and Social Drivers of Vaccination (BeSD) framework is the most suitable approach for addressing HPV vaccination in this setting. | Thank you for this suggestion. We have modified the introduction as suggested. |  |
| Key terms such as “low-resource environments” are also insufficiently described, and prior HPV prevention or vaccination approaches in Pakistan, along with their limitations related to behavioral and cultural factors, are not clearly described. Addressing these gaps is essential to establish a coherent rationale for the proposed framework development. | Thank you for this suggestion. We have included the required information in the introduction as suggested. | Page no.4-5  Line no. 77-104 |
| The Introduction does not adequately describe how behavioral, social, and cultural factors specific to Pakistan influence HPV vaccination decisions, nor does it report current HPV vaccination coverage, clarify the target population (female, male, or both, given that HPV vaccination is not limited to cervical cancer prevention), or present sex-specific HPV disease burden and vaccination patterns. | Thank you for this suggestion. We have included the required information in the introduction as suggested. | **Page no. 4,**  **Line no. 77-86** |
|  |  |  |
| The figure Methodological Framework for a Culturally Adapted HPV Vaccine Acceptance Study in Pakistan partially reflects the WHO BeSD framework; however, several core BeSD components are incompletely specified, and conceptual boundaries between domains are unclear. In particular, the addition of a “Cultural Integration” domain appears to overlap with existing BeSD constructs without sufficient justification. Please clarify the conceptual rationale for this domain and explicitly describe how it differs from, extends, or integrates with existing BeSD domains. | Thank you for your comments. We have clarified the conceptual rationale for this domain and other points as suggested. | Page no. 18-22  Line no. 382-470 |
| Please report participants’ HPV vaccination status and indicate whether vaccination experience was considered in sampling or analysis, as the absence of this information limits interpretation of the findings. | Thank you for your comments. Actually, our study participants' HPV vaccination status is not available, but other studies from the same are indicated that -----------------.  HPV vaccination status was not considered in sampling or analysis because, at the start of the study, the Pakistani government did not include the vaccination in its vaccination schedule. | Page no. 11.  Line no. 238-242 |
| Although 4 qualitative interview guides are mentioned, the Methods do not clearly describe how these guides differ in content, what domains each covers, or how they were aligned with the WHO BeSD framework. Please clarify these distinctions and provide sufficient detail to assess how the interview guides operationalized the framework. | Thank you for your comments. We have clarified these queries in the revised version of the manuscript. | Refer to supplementary file 01 |
| Please clarify the composition of all 4 focus group discussions, including the participant group for the fourth session and the approximate number of participants per group. | Thank you for your comments. We have clarified the composition of all 4 focus group discussions in the revised version of the manuscript. |  |
| Please provide greater analytic detail by clarifying how Braun and Clarke’s six-step thematic analysis was applied, how coding consistency was ensured across multiple coders, how transcript translation was conducted and verified, and what analytic criteria justified the creation of the additional “Cultural Integration” domain rather than integration within existing BeSD domains. | Thank you for your comments. We have clarified Braun and Clarke’s six-step thematic analysis and other queries in the revised version of the manuscript. | Page no. 15  Line no. 308-325 |

| It seems that the Methods and Results do not sufficiently demonstrate how qualitative findings were systematically synthesized into a distinct, culturally adapted BeSD-HPV framework. In particular, the process for preserving the conceptual integrity of the original BeSD domains and the analytic justification for introducing an additional domain are not clearly articulated, limiting confidence that the stated framework development objective was achieved. | Thank you for your comments. We have clarified how qualitative findings were systematically synthesized into a distinct, culturally adapted BeSD-HPV framework in the revised version of the manuscript. | Page no. 22, 16- 21  Line no. 466-470, 329-448 |
| --- | --- | --- |
| The Discussion partially addresses the Results and the stated study purpose. While it interprets key cultural and social drivers of HPV vaccine hesitancy and reiterates the rationale for adapting the WHO BeSD framework, it lacks a clear, concise summary of the main findings and does not consistently link interpretations back to the reported Results. | We appreciate this constructive feedback. We have revised the discussion to include a **concise synthesis of the key findings**, explicitly linking them back to the results. | Page no. 25-29  Line no. 521-623 |
| Comparison with prior BeSD-based studies is limited, and the conceptual contribution of the added “Cultural Integration” domain remains insufficiently articulated. It could be helpful to strengthen the Discussion by improving alignment with the Results, adopting a more cautious framing of the implications, and explicitly addressing future validation and application of the adapted framework. | Thank you for your comments. We have modified the discussion section and compared our findings with prior BeSD-based studies, and the conceptual contribution of the added “Cultural Integration” domain. | Page no. 28-31  Line no 595-659 |
| Please spell out the full term at first mention and use the abbreviation thereafter. | Thank you for pointing this out. We have checked the entire manuscript and ensured that, on first mention, the full term is used and the abbreviation thereafter. | Refer to first paragraph of Intro BeSD and LMIC full form mentioned |
| Please clearly identify all supplementary materials and provide complete legends for all figures. | Thank you for pointing this out. We have provided the identification of all supplementary materials and provided complete legends for all figures. | Figures; Page no. 10,22,23  Line no .209-210, 257, 488  Supplementary file  Page no. 12,21,22,23  Line no. 247,449,459,479 |
| Please review and revise the manuscript to ensure that all components comply with the journal’s submission guidelines. | We have checked, modified, and ensured that our manuscript follows PLOS ONE's style requirements. | Whole document |
