# Supplementary material for: From global to local: Developing a context-specific BeSD-HPV tool through cultural and linguistic adaptation in Pakistan
Source: PLoS One. 2026 Jun 15;21(6):e0350162. doi: 10.1371/journal.pone.0350162 (PMC13268181; doi:10.1371/journal.pone.0350162)
Supplement: Attachment — Submitted filename: 12 APRIL Rebuttal letter dated 6 april.docx [file pone.0350162.s012.docx]

PONE-D-25-55196R1

From Global to Local: Developing a Context-Specific BeSD-HPV Tool through Cultural and Linguistic Adaptation in Pakistan

PLOS One

PLOS ONE

Dear Editor,

We sincerely thank the reviewers for their thoughtful and constructive comments. We have carefully addressed the points raised and incorporated most of the suggestions in the revised manuscript. We believe that these revisions have substantially improved the clarity, rigor, and overall quality of the paper.

We remain fully open to further revisions and would be pleased to address any additional comments or recommendations.

Please find the modified manuscript. We have incorporated all suggestions recommended by the reviewers. Following is the point-wise reply to the reviewers' comments.

With best regards,

Dr Noor

| **Reviewer Comment** | **Author Response** | **Location in Manuscript** |
| --- | --- | --- |
| *1. Inconsistent Terminology and Redundancy (Throughout)Despite the language edit, several redundant or inconsistent terms persist, suggesting incomplete proofreading. Example (Abstract, Line 29-31): “Table cell colors are indicative of the domain thinking and feeling, social processes, motivation, practical issues, and emergent new theme cultural Integration as per colors aligned with the WHO BeSD Framework.” This sentence is convoluted. Consider: “Table cell colors correspond to the WHO BeSD domains (Thinking and Feeling, Social Processes, Motivation, Practical Issues) and the emergent theme, Cultural Integration.” Example (Page 13, Line 279-281): “Deeply committed constructs of BeSD” – “Deeply committed” is unclear. Did you mean “Core constructs” or “Original constructs”?* Example (Page 15, Line 311-313): The phrase “The transcripts were coded using the six steps of thematic analysis, as developed by Braun and Clarke, by researchers KN, SM, and MN, who also coded them independently” is awkwardly structured. Simplify. Action: Please conduct a final, line-by-line review to eliminate redundancy, correct unclear phrasing, and standardize terminology (e.g., consistently use “Thinking and Feeling” not “thinking and feeling” without capitalization when referring to the domain). | The manuscript has been checked and revised for inconsistent terminologies and redundancies. | **Page 4, Lines 29-30** (Abstract revised); **Page 16, Line 260** ("Core constructs" used); **Page 18, Lines 312-314** (Coding sentence simplified); **Throughout manuscript** (e.g., Page 28, Line 484 for standardized capitalization).  Few sentences have been deleted. |
| **2. Figure Quality and Self-Contained Legends** You have replaced copyrighted images and improved figure resolution. However, the legends for Figures 1, 2, and 3 are not fully self-contained. Figure 1 (Methodological Framework): The legend currently (Page 10, Line 209-210) is minimal. A reader should understand the figure without referring to the main text. Please add a brief legend that explains each step (e.g., “Step 1: Study Design & Setting; Step 2: Sampling & Recruitment; Step 3: Data Collection; Step 4: Analysis; Step 5: Final Outcome”). Figure 3 (Cultural Adaptation Process Aligned with TPB): The legend (Page 23, Line 488) simply states the title. Add 1-2 sentences explaining how TPB constructs map to BeSD domains (e.g., -“Attitudes → Thinking & Feeling; Subjective Norms → Social Processes; Perceived Behavioral Control → Practical Issues; Intention → Motivation; with Cultural Integration as a cross-cutting - domain”).Action: Revise all figure legends to be fully descriptive and self-contained. Ensure each figure file is -high-resolution (TIFF, ≥300 dpi) as previously requested. - | Make Figures 1, 2, and 3 legends self-contained (2-3 sentences each). | **Page 14, Lines 209-211** (Fig 1 legend); **Page 29, Lines 463-467** (Fig 2 legend); **Page 29-30, Lines 498-502** (Fig 3 legend). |
| **3. Missing Supplementary File Legend for S1 and Inconsistent Referencing** In the main text (Page 12, Line 247), you state that “Adapted Interview guides are also attached as supplementary file no 1.” However, in the “Supporting Information” section at the end of the manuscript (Page 53, Line 703-708), you list “S1: Four qualitative interview guides” without a descriptive legend. PLOS ONE requires a brief legend for each supporting file.Action: Immediately after the References section, provide a clear, numbered legend for each supplementary file. For example: S1. Qualitative interview guides for caregivers, healthcare providers, community influencers, and program managers (adapted from WHO BeSD). S2. De-identified interview transcripts. S3. Coding tree from thematic analysis. S4. Final 70-item BeSD-HPV tool (English and Urdu versions). Table S1. Thinking and Feeling domain – item mapping, quotes, and rationale. (Continue for S2–S5 and all supplementary tables)Also, ensure that every supplementary file is cited in the main text at least once. | Add descriptive legends for all supplementary files (S1–S5, Tables S1–S5). | **Page 39, Lines 712-721** (Supporting information section). Cited in main text at: **Page 16, Line 248** (S1); **Page 19, Line 329** (S2, S3); **Page 25, Line 455** (S3); **Page 29, Line 469** (S4); **Page 30, Line 489** (S1-S5, Tables S1-S5). |
| **4. Minor Ethical and Data Availability Clarifications** Ethics Statement (Page 52, Line 698-702): You state the study adhered to the Declaration of Helsinki. Please add the date or version of the Declaration (e.g., “as revised in 2013”) for precision. Data Availability (Page 17, Line 13 of the editorial section): You state “All relevant data are within the manuscript and its Supporting Information files.” However, the coding tree (S3) and transcripts (S2) are provided as supplementary files. Confirm explicitly that no additional raw data (e.g., NVivo project files) are required for replication, and that the de-identified transcripts contain no direct identifiers (names, specific dates, locations below district level). A one-sentence confirmation in the cover letter during resubmission will suffice. | Include version/date of Declaration of Helsinki. Confirm no additional raw data needed and transcripts are fully de-identified. | **Page 14, Lines 206-207** and **Page 39, Line 707** (Ethics statement updated to include "as revised in 2013"). Confirmation of data de-identification provided in cover letter. |
| 5. Overstated Claim in the Conclusion (Still Present) You have toned down implications, but the conclusion (Page 52, Line 672-678) still states: “This study provides evidence for the cultural adaptation of BeSD framework to develop a context-specific and linguistically appropriate tool… to support the successful launch of the HPV vaccine in Pakistan.” The phrase “to support the successful launch” implies predictive effectiveness. Given that the tool has not been quantitatively validated, revise to: “to inform the development of strategies for the planned introduction of the HPV vaccine in Pakistan” or “to provide a foundation for future implementation research.” Action: Minor rewording to align with the cautious framing you correctly adopted in the Discussion (Page 50, Line 632-634). | Replace “to support the successful launch” with more cautious wording. | **Page 38, Lines 681-685** (Conclusion revised to "to inform the development of strategies for the planned introduction... and to provide a foundation for future implementation research"). |
