# Supplementary material for: From global to local: Developing a context-specific BeSD-HPV tool through cultural and linguistic adaptation in Pakistan
Source: PLoS One. 2026 Jun 15;21(6):e0350162. doi: 10.1371/journal.pone.0350162 (PMC13268181; doi:10.1371/journal.pone.0350162)
Supplement: Attachment — Submitted filename: Rebuttal letter dated 21 April 2026 updated.docx [file pone.0350162.s013.docx]

PONE-D-25-55196R2-- [EMID:5a516c6c2efae7dd]

From Global to Local: Developing a Context-Specific BeSD-HPV Tool through Cultural and Linguistic Adaptation in Pakistan

PLOS One

Dear Editor,

We sincerely thank the reviewers for their thoughtful and constructive comments. We have

carefully addressed the points raised and incorporated most of the suggestions in the revised

manuscript. We believe that these revisions have substantially improved the clarity, rigor, and

overall quality of the paper.

We remain fully open to further revisions and would be pleased to address any additional

comments or recommendations.

Please find the modified manuscript. We have incorporated all suggestions recommended by the reviewers. Following is the point-wise reply to the reviewers; comments.

With best regards,

Dr Noor

| Journal Requirements | | |
| --- | --- | --- |
| Comments | Response | Reference |
| 1. Incomplete Correction of Abstract Wording (Page 4, Lines 29-30) In your response letter, you revised the abstract sentence to: “Table cell colors correspond to the WHO BeSD domains (Thinking and Feeling, Social Processes, Motivation, Practical Issues) and the emergent theme, Cultural Integration.” However, the manuscript file (page 4, lines 29-30) still contains the original convoluted wording. The revised version appears in the response letter but was not carried over to the manuscript file. Action: Replace the current abstract sentence with the corrected version as shown in your response letter. | Text updated in the abstract. | Page 2, Lines 29-30 |
| 2. Redundant Sentence in Introduction (Page 6, Lines 121-129 and Lines 130-134) You have two nearly identical paragraphs describing the target population (adolescent girls) and the rationale for excluding boys. The first paragraph (lines 121-129) is sufficient. The second paragraph (lines 130-134) begins "To provide a theoretically grounded basis..." and repeats TPB framing already covered elsewhere. Action: Delete the second redundant paragraph (lines 130-134). Keep the first paragraph as the sole justification. | Redundant paragraph removed. | Page 6, Lines 130-134 |
| 3. Typographical Error in Methods (Page 11, Line 226) The text reads: “Islamabad is the Capital Territory, and Rawalpindi comprises towns and urban areas with greater higher level of exposure...” Action: Change “greater higher level” to “greater level” or “higher level” (not both). | Phrase changed to "higher level". | Page 10, Line 222,223 |
| 4. Incomplete Sentence in Methods (Page 13, Line 274) The text reads: "This process helped in the following phenomenon to identify topics that require modification..." Action: This sentence is incomplete. Revise to: “This process helped to identify topics requiring modification in terms of rephrasing and contextual probing to enhance cultural relevance.” | Sentence revised for completeness and clarity. | Page 13, Line 272-278 |
| 5. Duplicate Phrase in Thematic Analysis Section (Page 17, Lines 346-348) The text reads: “In the first coding stage, all transcripts were initially looked at deductively to find out whether the narratives by the participants could be meaningfully and conceptually placed within the already established BeSD constructs and in- alignment with the corresponding TPB constructs. The Coding decisions in coding were determined by the theoretical purpose of each domain...” Action: Change “The Coding decisions in coding” to “Coding decisions” (remove redundancy). | Phrasing simplified to "Coding decisions". | Page 15-16, Lines 329-330 |
| 6. Missing Space in Figure 3 Reference (Page 24, Line 490) The text reads: “Figure 03 showillustrates the theoretical basis...” Action: Change to “Figure 3 illustrates the theoretical basis...” | Reference formatted correctly. | Page 23, Line 489-490 |
| 7. Inconsistent Capitalization of “Cultural Integration” Throughout the manuscript, “Cultural Integration” is sometimes capitalized as a proper domain name (correct) and sometimes written in lowercase. Examples: • Page 4, line 30: “emergent theme, Cultural Integration” (correct) • Page 26, line 540: “emergent domain 'cultural integration'” (incorrect, should be capitalized) Action: Standardize to “Cultural Integration” (capitalized) throughout the manuscript. | Terminology standardized to title case. | Page 26, Line 552,558-560  Page 5, Line 112 |
| 8. Missing Supporting File Citations in Main Text You have added descriptive legends for supplementary files at the end of the manuscript. However, several supplementary tables (S1-S5) are not cited in the main text. The journal requires that every supplementary file be cited at least once in the body of the manuscript. Action: Add brief citations for Tables S1-S5 in the Results or Methods section (e.g., "Detailed item mapping for each domain is provided in Supplementary Tables S1-S5"). | Citations for S1-S5 added to the main text. | Page 23, Line no.483-484 |
| 9. Minor Language Improvements (Optional but Recommended) Location Current Text Suggested Revision Page 5, line 88 “family setups is are mainly largely male-dominated” “family structures are predominantly male-dominated” Page 8, line 157 “BeSD domains eorrespond- closely align withe TPB's constructs” “BeSD domains align closely with TPB's constructs” Page 26, line 528 “participants' perceptions about of HPV vaccinations is are not just limited” “participants' perceptions of HPV vaccination are not limited” Page 29, line 613 “a reasonably opportunity” “a reasonable opportunity” | Suggested language revisions applied. | Pages 4, Line no 80-82  Page 7, Line 153  Page 25, Line 534-536  Page 26, Line 558-560 |
