# Supplementary material for: From global to local: Developing a context-specific BeSD-HPV tool through cultural and linguistic adaptation in Pakistan
Source: PLoS One. 2026 Jun 15;21(6):e0350162. doi: 10.1371/journal.pone.0350162 (PMC13268181; doi:10.1371/journal.pone.0350162)
Supplement: Peer review history

# Peer review: Decision Letter 0

**DOI:** 10.1371/journal.pone.0350162.r001

**Authors:** Morufu Raimi

15 Jan 2026

PONE-D-25-55196

From Global to Local: Developing a Context-Specific BeSD-HPV Tool through Cultural and Linguistic Adaptation in Pakistan

PLOS One

Dear Dr. Noor,

Thank you for submitting your manuscript to PLOS ONE. After careful consideration, we feel that it has merit but does not fully meet PLOS ONE’s publication criteria as it currently stands. Therefore, we invite you to submit a revised version of the manuscript that addresses the points raised during the review process.

Please submit your revised manuscript by Feb 28 2026 11:59PM. If you will need more time than this to complete your revisions, please reply to this message or contact the journal office at plosone@plos.org. When you're ready to submit your revision, log on to https://www.editorialmanager.com/pone/ and select the 'Submissions Needing Revision' folder to locate your manuscript file.

Please include the following items when submitting your revised manuscript:

- A letter that responds to each point raised by the academic editor and reviewer(s). You should upload this letter as a separate file labeled 'Response to Reviewers'.
- A marked-up copy of your manuscript that highlights changes made to the original version. You should upload this as a separate file labeled 'Revised Manuscript with Track Changes'.
- An unmarked version of your revised paper without tracked changes. You should upload this as a separate file labeled 'Manuscript'.

If you would like to make changes to your financial disclosure, please include your updated statement in your cover letter. Guidelines for resubmitting your figure files are available below the reviewer comments at the end of this letter.

If applicable, we recommend that you deposit your laboratory protocols in protocols.io to enhance the reproducibility of your results. Protocols.io assigns your protocol its own identifier (DOI) so that it can be cited independently in the future. For instructions see: https://journals.plos.org/plosone/s/submission-guidelines#loc-laboratory-protocols. Additionally, PLOS ONE offers an option for publishing peer-reviewed Lab Protocol articles, which describe protocols hosted on protocols.io. Read more information on sharing protocols at https://plos.org/protocols?utm_medium=editorial-email&utm_source=authorletters&utm_campaign=protocols.

We look forward to receiving your revised manuscript.

Kind regards,

Dr. Morufu Olalekan Raimi

Academic Editor

PLOS One

Journal Requirements:

When submitting your revision, we need you to address these additional requirements.

1. Please ensure that your manuscript meets PLOS ONE's style requirements, including those for file naming. The PLOS ONE style templates can be found at

https://journals.plos.org/plosone/s/file?id=wjVg/PLOSOne_formatting_sample_main_body.pdf and

https://journals.plos.org/plosone/s/file?id=ba62/PLOSOne_formatting_sample_title_authors_affiliations.pdf

2. We note that your Data Availability Statement is currently as follows: [All relevant data are within the manuscript and its Supporting Information files]

Please confirm at this time whether or not your submission contains all raw data required to replicate the results of your study. Authors must share the “minimal data set” for their submission. PLOS defines the minimal data set to consist of the data required to replicate all study findings reported in the article, as well as related metadata and methods (https://journals.plos.org/plosone/s/data-availability#loc-minimal-data-set-definition).

For example, authors should submit the following data:

- The values behind the means, standard deviations and other measures reported;

- The values used to build graphs;

- The points extracted from images for analysis.

Authors do not need to submit their entire data set if only a portion of the data was used in the reported study.

If your submission does not contain these data, please either upload them as Supporting Information files or deposit them to a stable, public repository and provide us with the relevant URLs, DOIs, or accession numbers. For a list of recommended repositories, please see https://journals.plos.org/plosone/s/recommended-repositories.

If there are ethical or legal restrictions on sharing a de-identified data set, please explain them in detail (e.g., data contain potentially sensitive information, data are owned by a third-party organization, etc.) and who has imposed them (e.g., an ethics committee). Please also provide contact information for a data access committee, ethics committee, or other institutional body to which data requests may be sent. If data are owned by a third party, please indicate how others may request data access.

3. Please upload a new copy of Figure 2 as the detail is not clear. Please follow the link for more information:  https://journals.plos.org/plosone/s/figures

4. We note that Figure 3 in your submission contains copyrighted images. All PLOS content is published under the Creative Commons Attribution License (CC BY 4.0), which means that the manuscript, images, and Supporting Information files will be freely available online, and any third party is permitted to access, download, copy, distribute, and use these materials in any way, even commercially, with proper attribution. For more information, see our copyright guidelines: http://journals.plos.org/plosone/s/licenses-and-copyright.

We require you to either (1) present written permission from the copyright holder to publish these figures specifically under the CC BY 4.0 license, or (2) remove the figures from your submission:

1. You may seek permission from the original copyright holder of Figure 3 to publish the content specifically under the CC BY 4.0 license.

We recommend that you contact the original copyright holder with the Content Permission Form (http://journals.plos.org/plosone/s/file?id=7c09/content-permission-form.pdf) and the following text:

“I request permission for the open-access journal PLOS ONE to publish XXX under the Creative Commons Attribution License (CCAL) CC BY 4.0 (http://creativecommons.org/licenses/by/4.0/). Please be aware that this license allows unrestricted use and distribution, even commercially, by third parties. Please reply and provide explicit written permission to publish XXX under a CC BY license and complete the attached form.”

Please upload the completed Content Permission Form or other proof of granted permissions as an "Other" file with your submission.

In the figure caption of the copyrighted figure, please include the following text: “Reprinted from [ref] under a CC BY license, with permission from [name of publisher], original copyright [original copyright year].”

2. If you are unable to obtain permission from the original copyright holder to publish these figures under the CC BY 4.0 license or if the copyright holder’s requirements are incompatible with the CC BY 4.0 license, please either i) remove the figure or ii) supply a replacement figure that complies with the CC BY 4.0 license. Please check copyright information on all replacement figures and update the figure caption with source information. If applicable, please specify in the figure caption text when a figure is similar but not identical to the original image and is therefore for illustrative purposes only.

5. We notice that your supplementary tables are included in the manuscript file. Please remove them and upload them with the file type 'Supporting Information'. Please ensure that each Supporting Information file has a legend listed in the manuscript after the references list.

6. We note that this data set consists of interview transcripts. Can you please confirm that all participants gave consent for interview transcript to be published?

If they DID provide consent for these transcripts to be published, please also confirm that the transcripts do not contain any potentially identifying information (or let us know if the participants consented to having their personal details published and made publicly available). We consider the following details to be identifying information:

- Names, nicknames, and initials

- Age more specific than round numbers

- GPS coordinates, physical addresses, IP addresses, email addresses

- Information in small sample sizes (e.g. 40 students from X class in X year at X university)

- Specific dates (e.g. visit dates, interview dates)

- ID numbers

Or, if the participants DID NOT provide consent for these transcripts to be published:

- Provide a de-identified version of the data or excerpts of interview responses

- Provide information regarding how these transcripts can be accessed by researchers who meet the criteria for access to confidential data, including:

a) the grounds for restriction

b) the name of the ethics committee, Institutional Review Board, or third-party organization that is imposing sharing restrictions on the data

c) a non-author, institutional point of contact that is able to field data access queries, in the interest of maintaining long-term data accessibility.

d) Any relevant data set names, URLs, DOIs, etc. that an independent researcher would need in order to request your minimal data set.

For further information on sharing data that contains sensitive participant information, please see: https://journals.plos.org/plosone/s/data-availability#loc-human-research-participant-data-and-other-sensitive-data

If there are ethical, legal, or third-party restrictions upon your dataset, you must provide all of the following details (https://journals.plos.org/plosone/s/data-availability#loc-acceptable-data-access-restrictions):

1. A complete description of the dataset

2. The nature of the restrictions upon the data (ethical, legal, or owned by a third party) and the reasoning behind them

3. The full name of the body imposing the restrictions upon your dataset (ethics committee, institution, data access committee, etc)

4. If the data are owned by a third party, confirmation of whether the authors received any special privileges in accessing the data that other researchers would not have

5. Direct, non-author contact information (preferably email) for the body imposing the restrictions upon the data, to which data access requests can be sent

If the reviewer comments include a recommendation to cite specific previously published works, please review and evaluate these publications to determine whether they are relevant and should be cited. There is no requirement to cite these works unless the editor has indicated otherwise.

Additional Editor Comments:

PLOS ONE Editorial Decision

Manuscript Number: PONE-D-25-55196

Title: From Global to Local: Developing a Context-Specific BeSD-HPV Tool through Cultural and Linguistic Adaptation in Pakistan

Corresponding Author: Dr. Mehreen Noor

Decision: Major Revision Required

Dear Dr. Noor and Co-authors,

Thank you for submitting your manuscript to PLOS ONE. We have now received comments from two reviewers, both of whom recognize the importance and relevance of your work in addressing HPV vaccine hesitancy in Pakistan through cultural adaptation of the WHO BeSD framework. While the reviewers find the study promising, they have raised substantial methodological, conceptual, and presentational concerns that must be adequately addressed before the manuscript can be considered for publication. As the handling editor, I have conducted a detailed assessment of the manuscript and concur with the reviewers’ critiques. The study has strong potential to contribute to the literature on culturally informed vaccine implementation, but several critical issues undermine its current scholarly rigor and clarity.

Below, I outline the core concerns that must be addressed in a revised submission:

Major Concerns Requiring Substantive Revision

1. Conceptual Clarity and Justification

• The Introduction does not sufficiently justify why Pakistan is a distinct context requiring a unique adaptation of the BeSD framework. The sociocultural, religious, and gendered nuances must be explicitly detailed with supporting literature.

• The added domain “Cultural Integration” lacks clear conceptual distinction from the existing BeSD domains (e.g., Social Processes, Thinking & Feeling). You must provide a rigorous theoretical and empirical justification for why this domain is necessary rather than integrating these constructs into existing domains. Overlap must be explicitly addressed.

• The target population (adolescent girls) and the rationale for excluding boys from HPV vaccination discussions need clarification, especially given global recommendations for gender-neutral HPV vaccination.

2. Methodological Rigor and Transparency

• Participant characteristics: Vaccination status of participants is missing. This is critical for interpreting perspectives on hesitancy vs. acceptance.

• Interview guides: The four qualitative guides are mentioned but not described. You must detail how each guide was tailored to different stakeholder groups and how they operationalized the BeSD domains.

• Focus Group Discussions: The composition of the fourth FGD is unspecified. Provide participant numbers and group demographics for all FGDs.

• Analytical process: While Braun & Clarke’s thematic analysis is cited, the manuscript lacks detail on coder agreement, resolution of discrepancies, and translation verification processes.

• Sampling justification: Purposive sampling is noted, but the rationale for selecting specific districts and stakeholders needs elaboration regarding representativeness and transferability.

3. Structural and Presentation Issues

• Tables are excessively large and disrupt narrative flow. Consider moving detailed tables (e.g., Tables I-V) to supplementary materials and retaining summarized versions in the main text.

• Figure clarity: Figures (e.g., methodological framework) require improved labeling and integration with the described process. Legends must be self-contained and clearly referenced.

• Language and flow: Several sections contain repetitive phrases, grammatical errors, and unclear syntax. A thorough language edit by a native English speaker or professional editing service is strongly recommended.

4. Discussion and Interpretation

• The Discussion does not sufficiently synthesize key findings or explicitly link back to the Results. A concise summary of main themes is needed.

• Comparative analysis with other BeSD adaptation studies (e.g., Nigeria, Bangladesh) is superficial. Strengthen this to highlight the unique contributions of your work.

• The implications of the “Cultural Integration” domain for policy and practice are overstated without validation. Frame conclusions more cautiously and emphasize the need for future quantitative validation of the adapted tool.

5. Compliance with Journal Guidelines

• Ensure all supplementary materials are clearly labeled and referenced.

• Abbreviations (e.g., BeSD, LMICs) must be defined at first use.

• Ethical approval details are included but should be clearly stated in the Methods section as per PLOS ONE requirements.

Recommendations for Revision

1. Restructure the Introduction to clearly articulate the gap in cultural adaptation of BeSD in Pakistan, supported by local data on HPV burden, vaccination coverage, and sociocultural barriers.

2. Clarify methodological steps in a dedicated subsection, detailing adaptation procedures, coder consistency, translation approach, and participant recruitment.

3. Refine the conceptual framework to explicitly distinguish the new “Cultural Integration” domain and justify its addition with qualitative evidence.

4. Condense and reorganize results: Use summary tables in the main text and provide extensive tables as supplementary files.

5. Strengthen the Discussion with a clear summary of findings, comparative context, limitations, and future research directions, particularly tool validation.

6. Undergo thorough language editing to improve clarity, coherence, and academic tone.

Revision Deadline: We expect to receive your revised manuscript within 90 days. Please submit a detailed point-by-point response to all reviewer and editor comments, noting changes made in the manuscript.

If you are unable to address these concerns within this timeframe, we will consider the manuscript withdrawn.

We appreciate your contribution to PLOS ONE and look forward to receiving a revised manuscript that meets the journal’s standards for publication.

Sincerely,

Dr. Morufu Olalekan Raimi

Editor, PLOS ONE

Deputy Director, Niger Delta Institute for Emerging and Re-emerging Infectious Diseases, Federal University Otuoke.

[Note: HTML markup is below. Please do not edit.]

Reviewers' comments:

Reviewer's Responses to Questions

**Comments to the Author**

1. Is the manuscript technically sound, and do the data support the conclusions?

The manuscript must describe a technically sound piece of scientific research with data that supports the conclusions. Experiments must have been conducted rigorously, with appropriate controls, replication, and sample sizes. The conclusions must be drawn appropriately based on the data presented.

Reviewer #1: Yes

Reviewer #2: Partly

**********

2. Has the statistical analysis been performed appropriately and rigorously?

Reviewer #1: Yes

Reviewer #2: N/A

**********

3. Have the authors made all data underlying the findings in their manuscript fully available?

The PLOS Data policy requires authors to make all data underlying the findings described in their manuscript fully available without restriction, with rare exception (please refer to the Data Availability Statement in the manuscript PDF file). The data should be provided as part of the manuscript or its supporting information, or deposited to a public repository. For example, in addition to summary statistics, the data points behind means, medians and variance measures should be available. If there are restrictions on publicly sharing data—e.g. participant privacy or use of data from a third party—those must be specified.

Reviewer #1: Yes

Reviewer #2: No

**********

4. Is the manuscript presented in an intelligible fashion and written in standard English?

PLOS ONE does not copyedit accepted manuscripts, so the language in submitted articles must be clear, correct, and unambiguous. Any typographical or grammatical errors should be corrected at revision, so please note any specific errors here.

Reviewer #1: Yes

Reviewer #2: No

**********

5. Review Comments to the Author

Please use the space provided to explain your answers to the questions above. You may also include additional comments for the author, including concerns about dual publication, research ethics, or publication ethics. (Please upload your review as an attachment if it exceeds 20,000 characters)

Reviewer #1: Thank you for submitting this important qualitative study focused on the cultural and linguistic adaptation of the WHO BeSD-HPV tool for Pakistan. The manuscript addresses a clear public health gap, is methodologically sound, and provides a well-articulated adaptation process grounded in rigorous qualitative inquiry.

The study employs a descriptive qualitative design that is appropriate for cultural adaptation work.

Use of Braun & Clarke’s six-step thematic analysis is well described and correctly implemented.

The data (verbatim quotes, themes, emergent domain) clearly support the conclusions.

Addition of a new domain, Cultural Integration, is strongly grounded in participant narratives.

Transparency in coding, construct development, and translation strengthens credibility.

Clear use of both deductive and inductive coding.

Trustworthiness is addressed comprehensively using Lincoln and Guba’s criteria (credibility, dependability, confirmability, transferability).

Reflexivity is described adequately.

Triangulation through IDIs and FGDs strengthens internal validity.

Minor Suggestion:

Indicate coder agreement or how discrepancies were resolved (e.g., consensus meetings, intercoder reliability).

Clarity and English Language Quality

The manuscript is clear, intelligible, and written in acceptable standard English, but minor language and formatting issues should be addressed.

Examples of recommended improvements:

Remove repeated words (e.g., “that that”, “includees”).

Areas for Strengthening Before Publication

Include a brief paragraph on the limitations, such as:

geographic focus on Punjab,

potential social desirability bias,

reliance on purposive sampling.

In the Discussion, more explicitly compare findings to other adaptation studies of BeSD tools globally.

Improve the clarity of tables some are extremely large for the main text.

Reviewer #2: Thank you for submitting your manuscript “From Global to Local: Developing a Context-Specific BeSD-HPV Tool through Cultural and Linguistic Adaptation in Pakistan” to PLOS One.

The study offers insights into the cultural, social, and gendered factors influencing HPV vaccine hesitancy in Pakistan, while important methodological and conceptual clarifications are needed to fully support the proposed adaptation of the BeSD framework.

Some revisions are suggested as follows:

Introduction: Please strengthen the Introduction by clearly defining the BeSD-HPV framework, explicitly justifying why Pakistan is the appropriate context, and explaining why the WHO Behavioral and Social Drivers of Vaccination (BeSD) framework is the most suitable approach for addressing HPV vaccination in this setting.

The Introduction does not adequately describe how behavioral, social, and cultural factors specific to Pakistan influence HPV vaccination decisions, nor does it report current HPV vaccination coverage, clarify the target population (female, male, or both, given that HPV vaccination is not limited to cervical cancer prevention), or present sex-specific HPV disease burden and vaccination patterns.

Key terms such as “low-resource environments” are also insufficiently described, and prior HPV prevention or vaccination approaches in Pakistan, along with their limitations related to behavioral and cultural factors, are not clearly described. Addressing these gaps is essential to establish a coherent rationale for the proposed framework development.

The figure Methodological Framework for a Culturally Adapted HPV Vaccine Acceptance Study in Pakistan partially reflects the WHO BeSD framework; however, several core BeSD components are incompletely specified, and conceptual boundaries between domains are unclear. In particular, the addition of a “Cultural Integration” domain appears to overlap with existing BeSD constructs without sufficient justification. Please clarify the conceptual rationale for this domain and explicitly describe how it differs from, extends, or integrates with existing BeSD domains.

Please report participants’ HPV vaccination status and indicate whether vaccination experience was considered in sampling or analysis, as the absence of this information limits interpretation of the findings.

Although 4 qualitative interview guides are mentioned, the Methods do not clearly describe how these guides differ in content, what domains each covers, or how they were aligned with the WHO BeSD framework. Please clarify these distinctions and provide sufficient detail to assess how the interview guides operationalized the framework.

Please clarify the composition of all 4 focus group discussions, including the participant group for the fourth session and the approximate number of participants per group.

Please provide greater analytic detail by clarifying how Braun and Clarke’s six-step thematic analysis was applied, how coding consistency was ensured across multiple coders, how transcript translation was conducted and verified, and what analytic criteria justified the creation of the additional “Cultural Integration” domain rather than integration within existing BeSD domains.

It seems that the Methods and Results do not sufficiently demonstrate how qualitative findings were systematically synthesized into a distinct, culturally adapted BeSD-HPV framework. In particular, the process for preserving the conceptual integrity of the original BeSD domains and the analytic justification for introducing an additional domain are not clearly articulated, limiting confidence that the stated framework development objective was achieved.

The Discussion partially addresses the Results and the stated study purpose. While it interprets key cultural and social drivers of HPV vaccine hesitancy and reiterates the rationale for adapting the WHO BeSD framework, it lacks a clear, concise summary of the main findings and does not consistently link interpretations back to the reported Results. Comparison with prior BeSD-based studies is limited, and the conceptual contribution of the added “Cultural Integration” domain remains insufficiently articulated. It could be helpful to strengthen the Discussion by improving alignment with the Results, adopting a more cautious framing of the implications, and explicitly addressing future validation and application of the adapted framework.

Please spell out the full term at first mention and use the abbreviation thereafter.

Please clearly identify all supplementary materials and provide complete legends for all figures.

Please review and revise the manuscript to ensure that all components comply with the journal’s submission guidelines.

**********

6. PLOS authors have the option to publish the peer review history of their article (what does this mean?). If published, this will include your full peer review and any attached files.

If you choose “no”, your identity will remain anonymous but your review may still be made public.

**Do you want your identity to be public for this peer review?**  For information about this choice, including consent withdrawal, please see our Privacy Policy.

Reviewer #1: No

Reviewer #2: No

**********

[NOTE: If reviewer comments were submitted as an attachment file, they will be attached to this email and accessible via the submission site. Please log into your account, locate the manuscript record, and check for the action link "View Attachments". If this link does not appear, there are no attachment files.]

To ensure your figures meet our technical requirements, please review our figure guidelines: https://journals.plos.org/plosone/s/figures

You may also use PLOS’s free figure tool, NAAS, to help you prepare publication quality figures: https://journals.plos.org/plosone/s/figures#loc-tools-for-figure-preparation

NAAS will assess whether your figures meet our technical requirements by comparing each figure against our figure specifications.

Attachment Submitted filename: Review notes.pdf

# Author comment: Author response to Decision Letter 1

**DOI:** 10.1371/journal.pone.0350162.r002

9 Mar 2026

From Global to Local: Developing a Context-Specific BeSD-

HPV Tool through Cultural and Linguistic Adaptation in

Pakistan

[PONE-D-25-55196] - [EMID:5f87472bf045244e]

PLOS ONE

Dear Editor,

We sincerely thank the reviewers for their thoughtful and constructive comments. We have carefully addressed the points raised and incorporated most of the suggestions in the revised manuscript. We believe that these revisions have substantially improved the clarity, rigor, and overall quality of the paper.

We remain fully open to further revisions and would be pleased to address any additional comments or recommendations.

Please find the modified manuscript. We have incorporated all suggestions recommended by the reviewers. Following is the point-wise reply to the reviewers' comments.

With best regards,

Dr Noor

Journal Requirements

Comments Response Reference

1. Please ensure that your manuscript meets PLOS ONE's style requirements, including those for file naming. The PLOS ONE style templates can be found at

https://journals.plos.org/plosone/s/file?id=wjVg/PLOSOne_formatting_sample_main_body.pdf and

https://journals.plos.org/plosone/s/file?id=ba62/PLOSOne_formatting_sample_title_authors_affiliations.pdf

We have revised the manuscript to fully comply with PLOS ONE formatting guidelines, including file naming, title page formatting, structured sections, and reference style.

Whole document

2. We note that your Data Availability Statement is currently as follows: [All relevant data are within the manuscript and its Supporting Information files]

Please confirm at this time whether or not your submission contains all raw data required to replicate the results of your study. Authors must share the “minimal data set” for their submission. PLOS defines the minimal data set to consist of the data required to replicate all study findings reported in the article, as well as related metadata and methods (https://journals.plos.org/plosone/s/data-availability#loc-minimal-data-set-definition).

For example, authors should submit the following data:

- The values behind the means, standard deviations and other measures reported;

- The values used to build graphs;

- The points extracted from images for analysis.

Authors do not need to submit their entire data set if only a portion of the data was used in the reported study.

If your submission does not contain these data, please either upload them as Supporting Information files or deposit them to a stable, public repository and provide us with the relevant URLs, DOIs, or accession numbers. For a list of recommended repositories, please see https://journals.plos.org/plosone/s/recommended-repositories.

If there are ethical or legal restrictions on sharing a de-identified data set, please explain them in detail (e.g., data contain potentially sensitive information, data are owned by a third-party organization, etc.) and who has imposed them (e.g., an ethics committee). Please also provide contact information for a data access committee, ethics committee, or other institutional body to which data requests may be sent. If data are owned by a third party, please indicate how others may request data access.

We confirm that the minimal de-identified dataset required to replicate the findings (coding Tree and Interview Transcripts) has been uploaded as Supporting Information. All identifying information has been removed. All relevant data are within the manuscript and its Supporting Information files. Refer to Supplementary File 1 and 2

3. Please upload a new copy of Figure 2 as the detail is not clear. Please follow the link for more information: https://journals.plos.org/plosone/s/figures

We have replaced Figure 2 with a higher-resolution version in TIFF format as per PLOS figure guidelines. Refer to Figure 2

4. We note that Figure 3 in your submission contains copyrighted images. All PLOS content is published under the Creative Commons Attribution License (CC BY 4.0), which means that the manuscript, images, and Supporting Information files will be freely available online, and any third party is permitted to access, download, copy, distribute, and use these materials in any way, even commercially, with proper attribution. For more information, see our copyright guidelines: http://journals.plos.org/plosone/s/licenses-and-copyright.

We require you to either (1) present written permission from the copyright holder to publish these figures specifically under the CC BY 4.0 license, or (2) remove the figures from your submission:

1. You may seek permission from the original copyright holder of Figure 3 to publish the content specifically under the CC BY 4.0 license.

We recommend that you contact the original copyright holder with the Content Permission Form (http://journals.plos.org/plosone/s/file?id=7c09/content-permission-form.pdf) and the following text:

“I request permission for the open-access journal PLOS ONE to publish XXX under the Creative Commons Attribution License (CCAL) CC BY 4.0 (http://creativecommons.org/licenses/by/4.0/). Please be aware that this license allows unrestricted use and distribution, even commercially, by third parties. Please reply and provide explicit written permission to publish XXX under a CC BY license and complete the attached form.”

Please upload the completed Content Permission Form or other proof of granted permissions as an "Other" file with your submission.

In the figure caption of the copyrighted figure, please include the following text: “Reprinted from [ref] under a CC BY license, with permission from [name of publisher], original copyright [original copyright year].”

2. If you are unable to obtain permission from the original copyright holder to publish these figures under the CC BY 4.0 license or if the copyright holder’s requirements are incompatible with the CC BY 4.0 license, please either i) remove the figure or ii) supply a replacement figure that complies with the CC BY 4.0 license. Please check copyright information on all replacement figures and update the figure caption with source information. If applicable, please specify in the figure caption text when a figure is similar but not identical to the original image and is therefore for illustrative purposes only. We have removed the copyrighted image and replaced it with an original figure created by the authors that conveys the same conceptual information while complying with CC BY 4.0 licensing requirements. Refer to Figure 3

5. We notice that your supplementary tables are included in the manuscript file. Please remove them and upload them with the file type 'Supporting Information'. Please ensure that each Supporting Information file has a legend listed in the manuscript after the references list. Supplementary tables have been removed from the manuscript and are being uploaded as a 'Supporting Information', All Supporting Information files are clearly labeled and referenced after the References section. Supplementary Tables S1, S2, S3, S4, S5

6. We note that this data set consists of interview transcripts. Can you please confirm that all participants gave consent for interview transcript to be published?

If they DID provide consent for these transcripts to be published, please also confirm that the transcripts do not contain any potentially identifying information (or let us know if the participants consented to having their personal details published and made publicly available). We consider the following details to be identifying information:

- Names, nicknames, and initials

- Age more specific than round numbers

- GPS coordinates, physical addresses, IP addresses, email addresses

- Information in small sample sizes (e.g. 40 students from X class in X year at X university)

- Specific dates (e.g. visit dates, interview dates)

- ID numbers

Or, if the participants DID NOT provide consent for these transcripts to be published:

- Provide a de-identified version of the data or excerpts of interview responses

- Provide information regarding how these transcripts can be accessed by researchers who meet the criteria for access to confidential data, including:

a) the grounds for restriction

b) the name of the ethics committee, Institutional Review Board, or third-party organization that is imposing sharing restrictions on the data

c) a non-author, institutional point of contact that is able to field data access queries, in the interest of maintaining long-term data accessibility.

d) Any relevant data set names, URLs, DOIs, etc. that an independent researcher would need in order to request your minimal data set.

For further information on sharing data that contains sensitive participant information, please see: https://journals.plos.org/plosone/s/data-availability#loc-human-research-participant-data-and-other-sensitive-data

If there are ethical, legal, or third-party restrictions upon your dataset, you must provide all of the following details (https://journals.plos.org/plosone/s/data-availability#loc-acceptable-data-access-restrictions):

1. A complete description of the dataset

2. The nature of the restrictions upon the data (ethical, legal, or owned by a third party) and the reasoning behind them

3. The full name of the body imposing the restrictions upon your dataset (ethics committee, institution, data access committee, etc)

4. If the data are owned by a third party, confirmation of whether the authors received any special privileges in accessing the data that other researchers would not have

5. Direct, non-author contact information (preferably email) for the body imposing the restrictions upon the data, to which data access requests can be sent

If the reviewer comments include a recommendation to cite specific previously published works, please review and evaluate these publications to determine whether they are relevant and should be cited. There is no requirement to cite these works unless the editor has indicated otherwise. All participants provided written informed consent for anonymized excerpts to be published. Transcripts were de-identified and do not contain names, specific dates, or identifiable details Supplementary file no. 2

1. Conceptual Clarity and Justification

• The Introduction does not sufficiently justify why Pakistan is a distinct context requiring a unique adaptation of the BeSD framework. The sociocultural, religious, and gendered nuances must be explicitly detailed with supporting literature. Thank you for pointing this out. We have added a new paragraph about Pakistan’s distinct context requiring a unique adaptation of the BeSD framework. The sociocultural, religious, and gendered nuances have been discussed in detail with supporting literature Page no. 04

Line no. 77–86

• The added domain “Cultural Integration” lacks clear conceptual distinction from the existing BeSD domains (e.g., Social Processes, Thinking & Feeling). You must provide a rigorous theoretical and empirical justification for why this domain is necessary rather than integrating these constructs into existing domains. Overlap must be explicitly addressed.

Thank you for pointing this out. We have added a conceptual framework and new paragraph explaining it.

Page no. 07 Line no.144–149; 188–197

• The target population (adolescent girls) and the rationale for excluding boys from HPV vaccination discussions need clarification, especially given global recommendations for gender-neutral HPV vaccination. We appreciate the reviewer’s comment and agree that clarification is needed. We have added a new paragraph in the introduction section. Page no. 06, Line no.121–129

2. Methodological Rigor and Transparency

• Participant characteristics: Vaccination status of participants is missing. This is critical for interpreting perspectives on hesitancy vs. acceptance.

We thank the reviewer for highlighting this important point. We agree that participants’ vaccination status is critical for interpreting perspectives on hesitancy versus acceptance.

However, during the data collection period, the HPV vaccination is yet to be included in the national immunization program in Pakistan, and hence none of the study participants (adolescent girls, parents, and community members) had been vaccinated against HPV. Thus, the views of the participants are based on expected acceptance, conditional acceptance, or reluctance toward a new vaccine, instead of the experience related to the previous uptake.

For other routine childhood vaccines, participants represented a mix of generally vaccine-accepting and vaccine-hesitant backgrounds, as inferred from their narratives and prior experiences (e.g., polio and COVID-19 vaccination), which are explicitly discussed in the results and discussion sections.

Page no. 11, Line no. 238–242

• Interview guides: The four qualitative guides are mentioned but not described. You must detail how each guide was tailored to different stakeholder groups and how they operationalized the BeSD domains.

We thank the reviewer for this comment and agree that further clarification was needed. In the revised manuscript, we have now explicitly described the four qualitative interview guides and how each was tailored to specific stakeholder groups while operationalizing the BeSD domains.

Adapted Interview guides are also attached as supplementary file no 1

Page no. 12, Line no.246–258

• Focus Group Discussions: The composition of the fourth FGD is unspecified. Provide participant numbers and group demographics for all FGDs.

We have revised the Methods section to provide detailed information on the composition of all four focus group discussions

Page no. 14, Line no. 289–298

• Analytical process: While Braun & Clarke’s thematic analysis is cited, the manuscript lacks detail on coder agreement, resolution of discrepancies, and translation verification processes.

Thank you for pointing this out.

We thank the reviewer for highlighting this point. We have now expanded the Analysis section to clarify the analytical rigor. These explanations have been included in order to enhance transparency and credibility of the analysis procedure.

Page no. 15, Line no. 311–321

• Sampling justification: Purposive sampling is noted, but the rationale for selecting specific districts and stakeholders needs elaboration regarding representativeness and transferability. We thank the reviewer for this comment. We have now clarified the rationale for both district selection and stakeholder inclusion to address issues of representativeness and transferability in the method section in the revised version. Page no. 11, Line no. 223–237

3. Structural and Presentation Issues

• Tables are excessively large and disrupt narrative flow. Consider moving detailed tables (e.g., Tables I-V) to supplementary materials and retaining summarized versions in the main text. Thank you for pointing this out. We have moved detailed tables (Tables I-V) to supplementary materials by retaining important findings in the result section.

Refer to Supplementary files

• Figure clarity: Figures (e.g., methodological framework) require improved labeling and integration with the described Figure 1, 2 and 3 has been revised for improved clarity, with all domains and steps clearly labeled. The legend has been rewritten to be self-contained, describing all processes. Refer to Figure 1, 2, 3

• Language and flow: Several sections contain repetitive phrases, grammatical errors, and unclear syntax. A thorough language edit by a native English speaker or professional editing service is strongly recommended. Thank you for pointing this out. The whole manuscript has been checked for spelling and grammar. General editing has been done throughout the manuscript. Additionally, a thorough language edit has been performed by a professional editing service. Whole document

Thorough language edit has been performed by a professional editing service.

4. Discussion and Interpretation

• The Discussion does not sufficiently synthesize key findings or explicitly link back to the Results. A concise summary of main themes is needed.

• Comparative analysis with other BeSD adaptation studies (e.g., Nigeria, Bangladesh) is superficial. Strengthen this

Attachment Submitted filename: Rebuttal Letter Updated.docx

# Editor report: Decision Letter 1

**DOI:** 10.1371/journal.pone.0350162.r003

**Authors:** Morufu Raimi

31 Mar 2026

PONE-D-25-55196R1

From Global to Local: Developing a Context-Specific BeSD-HPV Tool through Cultural and Linguistic Adaptation in Pakistan

PLOS One

Dear Dr.  Noor,

Thank you for submitting your manuscript to PLOS ONE. After careful consideration, we feel that it has merit but does not fully meet PLOS ONE’s publication criteria as it currently stands. Therefore, we invite you to submit a revised version of the manuscript that addresses the points raised during the review process.

Please submit your revised manuscript by May 15 2026 11:59PM. If you will need more time than this to complete your revisions, please reply to this message or contact the journal office at plosone@plos.org. When you're ready to submit your revision, log on to https://www.editorialmanager.com/pone/ and select the 'Submissions Needing Revision' folder to locate your manuscript file.

Please include the following items when submitting your revised manuscript:

- A letter that responds to each point raised by the academic editor and reviewer(s). You should upload this letter as a separate file labeled 'Response to Reviewers'.
- A marked-up copy of your manuscript that highlights changes made to the original version. You should upload this as a separate file labeled 'Revised Manuscript with Track Changes'.
- An unmarked version of your revised paper without tracked changes. You should upload this as a separate file labeled 'Manuscript'.

If you would like to make changes to your financial disclosure, please include your updated statement in your cover letter. Guidelines for resubmitting your figure files are available below the reviewer comments at the end of this letter.

If applicable, we recommend that you deposit your laboratory protocols in protocols.io to enhance the reproducibility of your results. Protocols.io assigns your protocol its own identifier (DOI) so that it can be cited independently in the future. For instructions see: https://journals.plos.org/plosone/s/submission-guidelines#loc-laboratory-protocols. Additionally, PLOS ONE offers an option for publishing peer-reviewed Lab Protocol articles, which describe protocols hosted on protocols.io. Read more information on sharing protocols at https://plos.org/protocols?utm_medium=editorial-email&utm_source=authorletters&utm_campaign=protocols.

As the corresponding author, your ORCID iD is verified in the submission system and will appear in the published article. PLOS supports the use of ORCID, and we encourage all coauthors to register for an ORCID iD and use it as well. Please encourage your coauthors to verify their ORCID iD within the submission system before final acceptance, as unverified ORCID iDs will not appear in the published article. *Only*  the individual author can complete the verification step; PLOS staff *cannot*  verify ORCID iDs on behalf of authors.

We look forward to receiving your revised manuscript.

Kind regards,

Morufu Olalekan Raimi, Ph.D

Academic Editor

PLOS One

Journal Requirements:

1. If the reviewer comments include a recommendation to cite specific previously published works, please review and evaluate these publications to determine whether they are relevant and should be cited. There is no requirement to cite these works unless the editor has indicated otherwise.

2. Please review your reference list to ensure that it is complete and correct. If you have cited papers that have been retracted, please include the rationale for doing so in the manuscript text, or remove these references and replace them with relevant current references. Any changes to the reference list should be mentioned in the rebuttal letter that accompanies your revised manuscript. If you need to cite a retracted article, indicate the article’s retracted status in the References list and also include a citation and full reference for the retraction notice.

Additional Editor Comments:

PLOS ONE Editorial Decision

Manuscript Number: PONE-D-25-55196R1

Title: From Global to Local: Developing a Context-Specific BeSD-HPV Tool through Cultural and Linguistic Adaptation in Pakistan

Corresponding Author: Dr. Mehreen Noor

Editor: Dr. Morufu Olalekan Raimi

Decision: Minor Revision Required

Date: March 31, 2026

Dear Dr. Noor and co-authors,

Thank you for submitting your revised manuscript and for the detailed, point-by-point response to the previous round of reviews. I have carefully evaluated the revised manuscript, the original reviewer comments, and your responses. You have made substantial efforts to address the major conceptual, methodological, and presentation concerns raised by the reviewers and myself as the handling editor. The manuscript has significantly improved. The introduction now more clearly justifies Pakistan’s distinct sociocultural context, the methods section provides greater transparency regarding coder agreement, FGD composition, and interview guide operationalization, and the discussion offers a more cautious framing of the “Cultural Integration” domain while calling for future quantitative validation. The relocation of extensive tables to supplementary files and the professional language editing have also enhanced readability. However, despite these improvements, a careful re-reading of the revised manuscript (including the clean and marked-up versions) reveals several residual issues that prevent me from accepting the manuscript in its current form. These are not as fundamental as the previous major concerns, but they require attention to meet PLOS ONE’s standards for clarity, consistency, and scientific rigor.

I therefore invite you to submit a minor revision of your manuscript, addressing the following points:

1. Inconsistent Terminology and Redundancy (Throughout)

Despite the language edit, several redundant or inconsistent terms persist, suggesting incomplete proofreading.

• Example (Abstract, Line 29-31): “Table cell colors are indicative of the domain thinking and feeling, social processes, motivation, practical issues, and emergent new theme cultural Integration as per colors aligned with the WHO BeSD Framework.” This sentence is convoluted. Consider: “Table cell colors correspond to the WHO BeSD domains (Thinking and Feeling, Social Processes, Motivation, Practical Issues) and the emergent theme, Cultural Integration.”

• Example (Page 13, Line 279-281): “Deeply committed constructs of BeSD” – “Deeply committed” is unclear. Did you mean “Core constructs” or “Original constructs”?

• Example (Page 15, Line 311-313): The phrase “The transcripts were coded using the six steps of thematic analysis, as developed by Braun and Clarke, by researchers KN, SM, and MN, who also coded them independently” is awkwardly structured. Simplify.

Action: Please conduct a final, line-by-line review to eliminate redundancy, correct unclear phrasing, and standardize terminology (e.g., consistently use “Thinking and Feeling” not “thinking and feeling” without capitalization when referring to the domain).

2. Figure Quality and Self-Contained Legends

You have replaced copyrighted images and improved figure resolution. However, the legends for Figures 1, 2, and 3 are not fully self-contained.

• Figure 1 (Methodological Framework): The legend currently (Page 10, Line 209-210) is minimal. A reader should understand the figure without referring to the main text. Please add a brief legend that explains each step (e.g., “Step 1: Study Design & Setting; Step 2: Sampling & Recruitment; Step 3: Data Collection; Step 4: Analysis; Step 5: Final Outcome”).

• Figure 3 (Cultural Adaptation Process Aligned with TPB): The legend (Page 23, Line 488) simply states the title. Add 1-2 sentences explaining how TPB constructs map to BeSD domains (e.g., “Attitudes → Thinking & Feeling; Subjective Norms → Social Processes; Perceived Behavioral Control → Practical Issues; Intention → Motivation; with Cultural Integration as a cross-cutting domain”).

Action: Revise all figure legends to be fully descriptive and self-contained. Ensure each figure file is high-resolution (TIFF, ≥300 dpi) as previously requested.

3. Missing Supplementary File Legend for S1 and Inconsistent Referencing

In the main text (Page 12, Line 247), you state that “Adapted Interview guides are also attached as supplementary file no 1.” However, in the “Supporting Information” section at the end of the manuscript (Page 53, Line 703-708), you list “S1: Four qualitative interview guides” without a descriptive legend. PLOS ONE requires a brief legend for each supporting file.

Action: Immediately after the References section, provide a clear, numbered legend for each supplementary file. For example:

• S1. Qualitative interview guides for caregivers, healthcare providers, community influencers, and program managers (adapted from WHO BeSD).

• S2. De-identified interview transcripts.

• S3. Coding tree from thematic analysis.

• S4. Final 70-item BeSD-HPV tool (English and Urdu versions).

• Table S1. Thinking and Feeling domain – item mapping, quotes, and rationale.

• (Continue for S2–S5 and all supplementary tables)

Also, ensure that every supplementary file is cited in the main text at least once.

4. Minor Ethical and Data Availability Clarifications

• Ethics Statement (Page 52, Line 698-702): You state the study adhered to the Declaration of Helsinki. Please add the date or version of the Declaration (e.g., “as revised in 2013”) for precision.

• Data Availability (Page 17, Line 13 of the editorial section): You state “All relevant data are within the manuscript and its Supporting Information files.” However, the coding tree (S3) and transcripts (S2) are provided as supplementary files. Confirm explicitly that no additional raw data (e.g., NVivo project files) are required for replication, and that the de-identified transcripts contain no direct identifiers (names, specific dates, locations below district level). A one-sentence confirmation in the cover letter during resubmission will suffice.

5. Overstated Claim in the Conclusion (Still Present)

You have toned down implications, but the conclusion (Page 52, Line 672-678) still states: “This study provides evidence for the cultural adaptation of BeSD framework to develop a context-specific and linguistically appropriate tool… to support the successful launch of the HPV vaccine in Pakistan.”

The phrase “to support the successful launch” implies predictive effectiveness. Given that the tool has not been quantitatively validated, revise to: “to inform the development of strategies for the planned introduction of the HPV vaccine in Pakistan” or “to provide a foundation for future implementation research.”

Action: Minor rewording to align with the cautious framing you correctly adopted in the Discussion (Page 50, Line 632-634).

________________________________________

Summary of Required Revisions (Minor)

Category Specific Action

Language & consistency Eliminate redundancy, clarify awkward phrasing, standardize domain capitalization.

Figure legends Make Figures 1, 2, and 3 legends self-contained (2-3 sentences each).

Supporting information Add descriptive legends for all supplementary files (S1–S5, Tables S1–S5).

Ethics statement Include version/date of Declaration of Helsinki.

Data availability Confirm no additional raw data needed and transcripts are fully de-identified.

Conclusion Replace “to support the successful launch” with more cautious wording.

________________________________________

Final Editorial Comment

This work addresses a critical gap in HPV vaccine implementation science for culturally complex, low-resource settings. The theoretical integration of TPB with the WHO BeSD framework and the empirically grounded “Cultural Integration” domain are potentially valuable contributions. However, the manuscript’s current clarity and presentation must meet PLOS ONE’s publication standards. I am confident that the remaining issues are minor and can be resolved within 14 days. Please submit your revised manuscript along with a point-by-point response to this decision letter, clearly indicating where each change has been made (page and line numbers).

I look forward to receiving your prompt revision.

Sincerely,

Dr. Morufu Olalekan Raimi

Editor, PLOS ONE

Deputy Director, Niger Delta Institute for Emerging and Re-emerging Infectious Diseases, Federal University Otuoke, Nigeria

# Author comment: Author response to Decision Letter 2

**DOI:** 10.1371/journal.pone.0350162.r004

15 Apr 2026

PONE-D-25-55196R1

From Global to Local: Developing a Context-Specific BeSD-HPV Tool through Cultural and

Linguistic Adaptation in Pakistan

PLOS One

PLOS ONE

Dear Editor,

We sincerely thank the reviewers for their thoughtful and constructive comments. We have

carefully addressed the points raised and incorporated most of the suggestions in the revised

manuscript. We believe that these revisions have substantially improved the clarity, rigor, and

overall quality of the paper.

We remain fully open to further revisions and would be pleased to address any additional

comments or recommendations.

Please find the modified manuscript. We have incorporated all suggestions recommended by the

reviewers. Following is the point-wise reply to the reviewers' comments.

With best regards,

Dr Noor

Reviewer Comment Author Response Location in Manuscript

1. Inconsistent Terminology and

Redundancy (Throughout)Despite the

language edit, several redundant or

inconsistent terms persist, suggesting

incomplete proofreading. Example

(Abstract, Line 29-31): “Table cell

colors are indicative of the domain

thinking and feeling, social processes,

motivation, practical issues, and

emergent new theme cultural Integration

as per colors aligned with the WHO

BeSD Framework.” This sentence is

convoluted. Consider: “Table cell colors

correspond to the WHO BeSD domains

(Thinking and Feeling, Social Processes,

Motivation, Practical Issues) and the

emergent theme, Cultural Integration.”

The manuscript has

been checked and

revised for

inconsistent

terminologies and

redundancies.

Page 4, Lines 29-30

(Abstract revised); Page 16,

Line 260 ("Core constructs"

used); Page 18, Lines 312-

314 (Coding sentence

simplified); Throughout

manuscript (e.g., Page 28,

Line 484 for standardized

capitalization).

Few sentences have been

deleted.

Example (Page 13, Line 279-281):

“Deeply committed constructs of BeSD”

– “Deeply committed” is unclear. Did

you mean “Core constructs” or

“Original constructs”? Example (Page

15, Line 311-313): The phrase “The

transcripts were coded using the six

steps of thematic analysis, as developed

by Braun and Clarke, by researchers KN,

SM, and MN, who also coded them

independently” is awkwardly structured.

Simplify. Action: Please conduct a final,

line-by-line review to eliminate

redundancy, correct unclear phrasing,

and standardize terminology (e.g.,

consistently use “Thinking and Feeling”

not “thinking and feeling” without

capitalization when referring to the

domain).

2. Figure Quality and Self-Contained

Legends You have replaced copyrighted

images and improved figure resolution.

However, the legends for Figures 1, 2,

and 3 are not fully self-contained.

Figure 1 (Methodological Framework):

The legend currently (Page 10, Line 209-

210) is minimal. A reader should

understand the figure without referring

to the main text. Please add a brief

legend that explains each step (e.g.,

“Step 1: Study Design & Setting; Step 2:

Sampling & Recruitment; Step 3: Data

Collection; Step 4: Analysis; Step 5:

Final Outcome”). Figure 3 (Cultural

Adaptation Process Aligned with TPB):

The legend (Page 23, Line 488) simply

states the title. Add 1-2 sentences

explaining how TPB constructs map to

BeSD domains (e.g., -“Attitudes →

Thinking & Feeling; Subjective Norms

→ Social Processes; Perceived

Behavioral Control → Practical Issues;

Intention → Motivation; with Cultural

Integration as a cross-cutting -

domain”).Action: Revise all figure

legends to be fully descriptive and self-

Make Figures 1, 2,

and 3 legends self-

contained (2-3

sentences each).

Page 14, Lines 209-211 (Fig

1 legend); Page 29, Lines

463-467 (Fig 2 legend); Page

29-30, Lines 498-502 (Fig 3

legend).

contained. Ensure each figure file is -

high-resolution (TIFF, ≥300 dpi) as

previously requested. -

3. Missing Supplementary File Legend

for S1 and Inconsistent Referencing In

the main text (Page 12, Line 247), you

state that “Adapted Interview guides are

also attached as supplementary file no

1.” However, in the “Supporting

Information” section at the end of the

manuscript (Page 53, Line 703-708), you

list “S1: Four qualitative interview

guides” without a descriptive legend.

PLOS ONE requires a brief legend for

each supporting file.Action: Immediately

after the References section, provide a

clear, numbered legend for each

supplementary file. For example: S1.

Qualitative interview guides for

caregivers, healthcare providers,

community influencers, and program

managers (adapted from WHO BeSD).

S2. De-identified interview transcripts.

S3. Coding tree from thematic analysis.

S4. Final 70-item BeSD-HPV tool

(English and Urdu versions). Table S1.

Thinking and Feeling domain – item

mapping, quotes, and rationale.

(Continue for S2–S5 and all

supplementary tables)Also, ensure that

every supplementary file is cited in the

main text at least once.

Add descriptive

legends for all

supplementary files

(S1–S5, Tables

S1–S5).

Page 39, Lines 712-721

(Supporting information

section). Cited in main text

at: Page 16, Line 248 (S1);

Page 19, Line 329 (S2, S3);

Page 25, Line 455 (S3);

Page 29, Line 469 (S4);

Page 30, Line 489 (S1-S5,

Tables S1-S5).

4. Minor Ethical and Data Availability

Clarifications Ethics Statement (Page

52, Line 698-702): You state the study

adhered to the Declaration of Helsinki.

Please add the date or version of the

Declaration (e.g., “as revised in 2013”)

for precision. Data Availability (Page

17, Line 13 of the editorial section): You

state “All relevant data are within the

manuscript and its Supporting

Information files.” However, the coding

tree (S3) and transcripts (S2) are

provided as supplementary files.

Confirm explicitly that no additional raw

Include version/date

of Declaration of

Helsinki. Confirm no

additional raw data

needed and

transcripts are fully

de-identified.

Page 14, Lines 206-207 and

Page 39, Line 707 (Ethics

statement updated to include

"as revised in 2013").

Confirmation of data de-

identification provided in

cover letter.

data (e.g., NVivo project files) are

required for replication, and that the de-

identified transcripts contain no direct

identifiers (names, specific dates,

locations below district level). A one-

sentence confirmation in the cover letter

during resubmission will suffice.

5. Overstated Claim in the Conclusion

(Still Present) You have toned down

implications, but the conclusion (Page

52, Line 672-678) still states: “This

study provides evidence for the cultural

adaptation of BeSD framework to

develop a context-specific and

linguistically appropriate tool… to

support the successful launch of the

HPV vaccine in Pakistan.” The phrase

“to support the successful launch”

implies predictive effectiveness. Given

that the tool has not been quantitatively

validated, revise to: “to inform the

development of strategies for the

planned introduction of the HPV vaccine

in Pakistan” or “to provide a foundation

for future implementation research.”

Action: Minor rewording to align with

the cautious framing you correctly

adopted in the Discussion (Page 50, Line

632-634).

Replace “to support

the successful

launch” with more

cautious wording.

Page 38, Lines 681-685

(Conclusion revised to "to

inform the development of

strategies for the planned

introduction... and to provide

a foundation for future

implementation research").

Attachment Submitted filename: 12 APRIL Rebuttal letter dated 6 april.docx

# Editor report: Decision Letter 2

**DOI:** 10.1371/journal.pone.0350162.r005

**Authors:** Morufu Raimi

20 Apr 2026

PONE-D-25-55196R2

From Global to Local: Developing a Context-Specific BeSD-HPV Tool through Cultural and Linguistic Adaptation in Pakistan

PLOS One

Dear Dr. Noor,

Thank you for submitting your manuscript to PLOS ONE. After careful consideration, we feel that it has merit but does not fully meet PLOS ONE’s publication criteria as it currently stands. Therefore, we invite you to submit a revised version of the manuscript that addresses the points raised during the review process.

Please submit your revised manuscript by Jun 04 2026 11:59PM. If you will need more time than this to complete your revisions, please reply to this message or contact the journal office at plosone@plos.org. When you're ready to submit your revision, log on to https://www.editorialmanager.com/pone/ and select the 'Submissions Needing Revision' folder to locate your manuscript file.

Please include the following items when submitting your revised manuscript:

- A letter that responds to each point raised by the academic editor and reviewer(s). You should upload this letter as a separate file labeled 'Response to Reviewers'.
- A marked-up copy of your manuscript that highlights changes made to the original version. You should upload this as a separate file labeled 'Revised Manuscript with Track Changes'.
- An unmarked version of your revised paper without tracked changes. You should upload this as a separate file labeled 'Manuscript'.

If you would like to make changes to your financial disclosure, please include your updated statement in your cover letter. Guidelines for resubmitting your figure files are available below the reviewer comments at the end of this letter.

If applicable, we recommend that you deposit your laboratory protocols in protocols.io to enhance the reproducibility of your results. Protocols.io assigns your protocol its own identifier (DOI) so that it can be cited independently in the future. For instructions see: https://journals.plos.org/plosone/s/submission-guidelines#loc-laboratory-protocols. Additionally, PLOS ONE offers an option for publishing peer-reviewed Lab Protocol articles, which describe protocols hosted on protocols.io. Read more information on sharing protocols at https://plos.org/protocols?utm_medium=editorial-email&utm_source=authorletters&utm_campaign=protocols.

As the corresponding author, your ORCID iD is verified in the submission system and will appear in the published article. PLOS supports the use of ORCID, and we encourage all coauthors to register for an ORCID iD and use it as well. Please encourage your coauthors to verify their ORCID iD within the submission system before final acceptance, as unverified ORCID iDs will not appear in the published article. *Only*  the individual author can complete the verification step; PLOS staff *cannot*  verify ORCID iDs on behalf of authors.

We look forward to receiving your revised manuscript.

Kind regards,

Morufu Olalekan Raimi, Ph.D

Academic Editor

PLOS One

Journal Requirements:

If the reviewer comments include a recommendation to cite specific previously published works, please review and evaluate these publications to determine whether they are relevant and should be cited. There is no requirement to cite these works unless the editor has indicated otherwise.

Please review your reference list to ensure that it is complete and correct. If you have cited papers that have been retracted, please include the rationale for doing so in the manuscript text, or remove these references and replace them with relevant current references. Any changes to the reference list should be mentioned in the rebuttal letter that accompanies your revised manuscript. If you need to cite a retracted article, indicate the article’s retracted status in the References list and also include a citation and full reference for the retraction notice.

Additional Editor Comments:

REVIEWER'S REPORT

Title: From Global to Local: Developing a Context-Specific BeSD-HPV Tool through Cultural and Linguistic Adaptation in Pakistan

Recommendation: Minor Revision

Editor: Dr. Morufu Olalekan Raimi, PLOS ONE Academic Editor

Review Date: April 17, 2026

EDITORIAL DECISION

Dear Authors,

Thank you for submitting your revised manuscript and for the detailed, point-by-point response to the previous reviews. You have addressed the major conceptual, methodological, and structural concerns raised in the prior round substantively and professionally. The manuscript is now considerably stronger: the introduction better justifies Pakistan's distinct context, the "Cultural Integration" domain is more clearly distinguished from existing BeSD constructs, the methodological transparency has improved, and the discussion is appropriately cautious about the need for future quantitative validation. However, after a thorough review of the revised manuscript and your response letter, several minor but necessary issues remain. These do not require major re-analysis or additional data collection, but they must be corrected before the manuscript is ready for publication.

REQUIRED MINOR REVISIONS

1. Incomplete Correction of Abstract Wording (Page 4, Lines 29-30)

In your response letter, you revised the abstract sentence to: “Table cell colors correspond to the WHO BeSD domains (Thinking and Feeling, Social Processes, Motivation, Practical Issues) and the emergent theme, Cultural Integration.” However, the manuscript file (page 4, lines 29-30) still contains the original convoluted wording. The revised version appears in the response letter but was not carried over to the manuscript file.

Action: Replace the current abstract sentence with the corrected version as shown in your response letter.

2. Redundant Sentence in Introduction (Page 6, Lines 121-129 and Lines 130-134)

You have two nearly identical paragraphs describing the target population (adolescent girls) and the rationale for excluding boys. The first paragraph (lines 121-129) is sufficient. The second paragraph (lines 130-134) begins "To provide a theoretically grounded basis..." and repeats TPB framing already covered elsewhere.

Action: Delete the second redundant paragraph (lines 130-134). Keep the first paragraph as the sole justification.

3. Typographical Error in Methods (Page 11, Line 226)

The text reads: “Islamabad is the Capital Territory, and Rawalpindi comprises towns and urban areas with greater higher level of exposure...”

Action: Change “greater higher level” to “greater level” or “higher level” (not both).

4. Incomplete Sentence in Methods (Page 13, Line 274)

The text reads: "This process helped in the following phenomenon to identify topics that require modification..."

Action: This sentence is incomplete. Revise to: “This process helped to identify topics requiring modification in terms of rephrasing and contextual probing to enhance cultural relevance.”

5. Duplicate Phrase in Thematic Analysis Section (Page 17, Lines 346-348)

The text reads: “In the first coding stage, all transcripts were initially looked at deductively to find out whether the narratives by the participants could be meaningfully and conceptually placed within the already established BeSD constructs and in- alignment with the corresponding TPB constructs. The Coding decisions in coding were determined by the theoretical purpose of each domain...”

Action: Change “The Coding decisions in coding” to “Coding decisions” (remove redundancy).

6. Missing Space in Figure 3 Reference (Page 24, Line 490)

The text reads: “Figure 03 showillustrates the theoretical basis...”

Action: Change to “Figure 3 illustrates the theoretical basis...”

7. Inconsistent Capitalization of “Cultural Integration”

Throughout the manuscript, “Cultural Integration” is sometimes capitalized as a proper domain name (correct) and sometimes written in lowercase. Examples:

• Page 4, line 30: “emergent theme, Cultural Integration” (correct)

• Page 26, line 540: “emergent domain 'cultural integration'” (incorrect, should be capitalized)

Action: Standardize to “Cultural Integration” (capitalized) throughout the manuscript.

8. Missing Supporting File Citations in Main Text

You have added descriptive legends for supplementary files at the end of the manuscript. However, several supplementary tables (S1-S5) are not cited in the main text. The journal requires that every supplementary file be cited at least once in the body of the manuscript.

Action: Add brief citations for Tables S1-S5 in the Results or Methods section (e.g., "Detailed item mapping for each domain is provided in Supplementary Tables S1-S5").

9. Minor Language Improvements (Optional but Recommended)

Location Current Text Suggested Revision

Page 5, line 88 “family setups is are mainly largely male-dominated” “family structures are predominantly male-dominated”

Page 8, line 157 “BeSD domains eorrespond- closely align withe TPB's constructs” “BeSD domains align closely with TPB's constructs”

Page 26, line 528 “participants' perceptions about of HPV vaccinations is are not just limited” “participants' perceptions of HPV vaccination are not limited”

Page 29, line 613 “a reasonably opportunity” “a reasonable opportunity”

STRENGTHS OF THE REVISED MANUSCRIPT

The authors deserve credit for several substantive improvements:

1. Clearer justification for Pakistan as a distinct context - The introduction now explicitly details sociocultural, religious, and gendered barriers with supporting literature.

2. Improved conceptual distinction of “Cultural Integration” - The revised framework clarifies how this domain differs from (and interacts with) existing BeSD constructs, particularly through the TPB alignment.

3. Enhanced methodological transparency - The addition of coder agreement procedures, translation verification, and detailed FGD composition strengthens credibility.

4. Appropriately cautious conclusions - The discussion now emphasizes the need for future quantitative validation and tones down overstatements about policy impact.

5. Responsive to all reviewer concerns - The point-by-point response is thorough and professional.

RECOMMENDATION

Minor Revision

The manuscript is now methodologically sound and makes a meaningful contribution to the cultural adaptation of global health frameworks. The remaining issues are minor and can be corrected without additional review. Once the 9 items listed above are addressed, the manuscript will be suitable for publication.

SUBMISSION INSTRUCTIONS

Please prepare a revised manuscript addressing the required corrections. In your cover letter, provide a brief point-by-point response to each of the 9 items, citing page and line numbers. Use tracked changes or a colored font to highlight revisions.

Respectfully,

Dr. Morufu Olalekan Raimi

Academic Editor, PLOS ONE

[Note: HTML markup is below. Please do not edit.]

To ensure your figures meet our technical requirements, please review our figure guidelines: https://journals.plos.org/plosone/s/figures

You may also use PLOS’s free figure tool, NAAS, to help you prepare publication quality figures: https://journals.plos.org/plosone/s/figures#loc-tools-for-figure-preparation.

NAAS will assess whether your figures meet our technical requirements by comparing each figure against our figure specifications.

# Author comment: Author response to Decision Letter 3

**DOI:** 10.1371/journal.pone.0350162.r006

28 Apr 2026

PONE-D-25-55196R2-- [EMID:5a516c6c2efae7dd]

From Global to Local: Developing a Context-Specific BeSD-HPV Tool through Cultural and Linguistic Adaptation in Pakistan

PLOS One

Dear Editor,

We sincerely thank the reviewers for their thoughtful and constructive comments. We have

carefully addressed the points raised and incorporated most of the suggestions in the revised

manuscript. We believe that these revisions have substantially improved the clarity, rigor, and

overall quality of the paper.

We remain fully open to further revisions and would be pleased to address any additional

comments or recommendations.

Please find the modified manuscript. We have incorporated all suggestions recommended by the reviewers. Following is the point-wise reply to the reviewers; comments.

With best regards,

Dr Noor

Journal Requirements

Comments Response Reference

1. Incomplete Correction of Abstract Wording (Page 4, Lines 29-30)

In your response letter, you revised the abstract sentence to: “Table cell colors correspond to the WHO BeSD domains (Thinking and Feeling, Social Processes, Motivation, Practical Issues) and the emergent theme, Cultural Integration.” However, the manuscript file (page 4, lines 29-30) still contains the original convoluted wording. The revised version appears in the response letter but was not carried over to the manuscript file.

Action: Replace the current abstract sentence with the corrected version as shown in your response letter. Text updated in the abstract. Page 2, Lines 29-30

2. Redundant Sentence in Introduction (Page 6, Lines 121-129 and Lines 130-134)

You have two nearly identical paragraphs describing the target population (adolescent girls) and the rationale for excluding boys. The first paragraph (lines 121-129) is sufficient. The second paragraph (lines 130-134) begins "To provide a theoretically grounded basis..." and repeats TPB framing already covered elsewhere.

Action: Delete the second redundant paragraph (lines 130-134). Keep the first paragraph as the sole justification. Redundant paragraph removed. Page 6, Lines 130-134

3. Typographical Error in Methods (Page 11, Line 226)

The text reads: “Islamabad is the Capital Territory, and Rawalpindi comprises towns and urban areas with greater higher level of exposure...”

Action: Change “greater higher level” to “greater level” or “higher level” (not both). Phrase changed to "higher level". Page 10, Line 222,223

4. Incomplete Sentence in Methods (Page 13, Line 274)

The text reads: "This process helped in the following phenomenon to identify topics that require modification..."

Action: This sentence is incomplete. Revise to: “This process helped to identify topics requiring modification in terms of rephrasing and contextual probing to enhance cultural relevance.” Sentence revised for completeness and clarity. Page 13, Line 272-278

5. Duplicate Phrase in Thematic Analysis Section (Page 17, Lines 346-348)

The text reads: “In the first coding stage, all transcripts were initially looked at deductively to find out whether the narratives by the participants could be meaningfully and conceptually placed within the already established BeSD constructs and in- alignment with the corresponding TPB constructs. The Coding decisions in coding were determined by the theoretical purpose of each domain...”

Action: Change “The Coding decisions in coding” to “Coding decisions” (remove redundancy). Phrasing simplified to "Coding decisions". Page 15-16, Lines 329-330

6. Missing Space in Figure 3 Reference (Page 24, Line 490)

The text reads: “Figure 03 showillustrates the theoretical basis...”

Action: Change to “Figure 3 illustrates the theoretical basis...” Reference formatted correctly. Page 23, Line 489-490

7. Inconsistent Capitalization of “Cultural Integration”

Throughout the manuscript, “Cultural Integration” is sometimes capitalized as a proper domain name (correct) and sometimes written in lowercase. Examples:

• Page 4, line 30: “emergent theme, Cultural Integration” (correct)

• Page 26, line 540: “emergent domain 'cultural integration'” (incorrect, should be capitalized)

Action: Standardize to “Cultural Integration” (capitalized) throughout the manuscript. Terminology standardized to title case. Page 26, Line 552,558-560

Page 5, Line 112

8. Missing Supporting File Citations in Main Text

You have added descriptive legends for supplementary files at the end of the manuscript. However, several supplementary tables (S1-S5) are not cited in the main text. The journal requires that every supplementary file be cited at least once in the body of the manuscript.

Action: Add brief citations for Tables S1-S5 in the Results or Methods section (e.g., "Detailed item mapping for each domain is provided in Supplementary Tables S1-S5"). Citations for S1-S5 added to the main text. Page 23, Line no.483-484

9. Minor Language Improvements (Optional but Recommended)

Location Current Text Suggested Revision

Page 5, line 88 “family setups is are mainly largely male-dominated” “family structures are predominantly male-dominated”

Page 8, line 157 “BeSD domains eorrespond- closely align withe TPB's constructs” “BeSD domains align closely with TPB's constructs”

Page 26, line 528 “participants' perceptions about of HPV vaccinations is are not just limited” “participants' perceptions of HPV vaccination are not limited”

Page 29, line 613 “a reasonably opportunity” “a reasonable opportunity” Suggested language revisions applied. Pages 4, Line no 80-82

Page 7, Line 153

Page 25, Line 534-536

Page 26, Line 558-560

Attachment Submitted filename: Rebuttal letter dated 21 April 2026 updated.docx

# Editor report: Decision Letter 3

**DOI:** 10.1371/journal.pone.0350162.r007

**Authors:** Morufu Raimi

10 May 2026

From Global to Local: Developing a Context-Specific BeSD-HPV Tool through Cultural and Linguistic Adaptation in Pakistan

PONE-D-25-55196R3

Dear Authors,

We’re pleased to inform you that your manuscript has been judged scientifically suitable for publication and will be formally accepted for publication once it meets all outstanding technical requirements.

Within one week, you’ll receive an e-mail detailing the required amendments. When these have been addressed, you’ll receive a formal acceptance letter and your manuscript will be scheduled for publication.

An invoice will be generated when your article is formally accepted. Please note, if your institution has a publishing partnership with PLOS and your article meets the relevant criteria, all or part of your publication costs will be covered. Please make sure your user information is up-to-date by logging into Editorial Manager at Editorial Manager® and clicking the ‘Update My Information' link at the top of the page. For questions related to billing, please contact billing support.

If your institution or institutions have a press office, please notify them about your upcoming paper to help maximize its impact. If they’ll be preparing press materials, please inform our press team as soon as possible -- no later than 48 hours after receiving the formal acceptance. Your manuscript will remain under strict press embargo until 2 pm Eastern Time on the date of publication. For more information, please contact onepress@plos.org.

Kind regards,

Morufu Olalekan Raimi, Ph.D

Academic Editor

PLOS One

Additional Editor Comments (optional):

PLOS ONE Editorial Decision

Manuscript Number: PONE-D-25-55196R3

Title: From Global to Local: Developing a Context-Specific BeSD-HPV Tool through Cultural and Linguistic Adaptation in Pakistan

Authors: Khola Noreen, Mehreen Noor, Saba Maryam, Mukhtiar Baig

Academic Editor: Dr. Morufu Olalekan Raimi

Decision: Accept

Summary of Evaluation

The authors have thoroughly and systematically addressed all required minor revisions from the previous round. Each of the nine specific issues raised in my prior decision (abstract wording, redundant paragraph, typographical error, incomplete sentence, duplicate phrase, missing space, inconsistent capitalization of “Cultural Integration,” missing supplementary file citations, and minor language improvements) has been corrected in the manuscript. The revised text is now clear, consistent, and compliant with journal standards.

Key Confirmations

1. Abstract – Corrected wording now appears on page 2, lines 29-30 as specified.

2. Introduction – Redundant paragraph removed; justification for adolescent girls as the target population is now concise and non-repetitive.

3. Methods – Typographical error (“greater higher level” → “higher level”) and incomplete sentence have been fixed; supporting file citations (S1–S5) are now included in the main text (page 23, lines 483-484).

4. Thematic Analysis – Duplicate phrasing (“Coding decisions in coding” → “Coding decisions”) resolved.

5. Capitalization – “Cultural Integration” is now consistently capitalized throughout as a proper domain name.

6. Figure reference – Corrected to “Figure 3 illustrates…”

7. Language – Minor grammatical improvements incorporated as recommended.

No further revisions are required.

The manuscript meets PLOS ONE’s criteria for publication: it presents methodologically sound qualitative research, offers a meaningful cultural adaptation of a global health framework, and is reported with sufficient transparency (COREQ guidelines, trustworthiness criteria, supplementary materials). The added “Cultural Integration” domain is conceptually justified, and the authors appropriately acknowledge the need for future quantitative validation.

Final Recommendation

Accepted in current form.

I congratulate the authors on a well-executed study and a responsive revision process.

Sincerely,

Dr. Morufu Olalekan Raimi

Academic Editor, PLOS ONE

Reviewers' comments:

# Editor report: Acceptance letter

**DOI:** 10.1371/journal.pone.0350162.r008

**Authors:** Morufu Raimi

PONE-D-25-55196R3

PLOS One

Dear Dr. Noor,

I'm pleased to inform you that your manuscript has been deemed suitable for publication in PLOS One. Congratulations! Your manuscript is now being handed over to our production team.

At this stage, our production department will prepare your paper for publication. This includes ensuring the following:

* All references, tables, and figures are properly cited

* All relevant supporting information is included in the manuscript submission,

* There are no issues that prevent the paper from being properly typeset

You will receive further instructions from the production team, including instructions on how to review your proof when it is ready. Please keep in mind that we are working through a large volume of accepted articles, so please give us a few days to review your paper and let you know the next and final steps.

Lastly, if your institution or institutions have a press office, please let them know about your upcoming paper now to help maximize its impact. If they'll be preparing press materials, please inform our press team within the next 48 hours. Your manuscript will remain under strict press embargo until 2 pm Eastern Time on the date of publication. For more information, please contact onepress@plos.org.

You will receive an invoice from PLOS for your publication fee after your manuscript has reached the completed accept phase. If you receive an email requesting payment before acceptance or for any other service, this may be a phishing scheme. Learn how to identify phishing emails and protect your accounts at https://explore.plos.org/phishing.

If we can help with anything else, please email us at customercare@plos.org.

Thank you for submitting your work to PLOS ONE and supporting open access.

Kind regards,

PLOS ONE Editorial Office Staff

on behalf of

Prof Morufu Olalekan Raimi

Academic Editor

PLOS One
